# Compatibility of state constraints and dynamics for multiagent control systems

**DOI:** 10.1007/s00028-021-00724-z

**Published:** 2021-06-14

**Authors:** Giulia Cavagnari, Antonio Marigonda, Marc Quincampoix

**Affiliations:** 1 Dipartimento di Matematica “F. Brioschi”, Politecnico di Milano, Piazza Leonardo da Vinci 32, 20133 Milan, Italy; 2 Department of Computer Science, University of Verona, Strada Le Grazie 15, 37134 Verona, Italy; 3 Laboratoire de Mathématiques de Bretagne Atlantique, UMR CNRS 6205, Univ Brest, 6, Avenue Victor Le Gorgeu, 29200 Brest, France

**Keywords:** Optimal control, Optimal transport, Hamilton–Jacobi–Bellman equation, Multi-agent, 49J15, 49J52, 49L25, 49K27, 49K21, 49Q20, 34A60, 93C15

## Abstract

This study concerns the problem of compatibility of state constraints with a multiagent control system. Such a system deals with a number of agents so large that only a statistical description is available. For this reason, the state variable is described by a probability measure on ${\mathbb {R}}^d$ representing the density of the agents and evolving according to the so-called continuity equation which is an equation stated in the Wasserstein space of probability measures. The aim of the paper is to provide a necessary and sufficient condition for a given constraint (a closed subset of the Wasserstein space) to be compatible with the controlled continuity equation. This new condition is characterized in a viscosity sense as follows: the distance function to the constraint set is a viscosity supersolution of a suitable Hamilton–Jacobi–Bellman equation stated on the Wasserstein space. As a byproduct and key ingredient of our approach, we obtain a new comparison theorem for evolutionary Hamilton–Jacobi equations in the Wasserstein space.

## Introduction

In classical control theory, a single agent controls a dynamics (here represented by a differential inclusion)

1.1 $$\begin{aligned} \dot{x}(t) \in F(x(t)),&x(0)=x_0 \, , \end{aligned}$$

where $F:{\mathbb {R}}^d \rightrightarrows {\mathbb {R}}^d$ is a set valued map, associating with each $x\in {\mathbb {R}}^d$ the subset *F*(*x*) of ${\mathbb {R}}^d$ of the admissible velocities from *x*. A *multiagent system* involves a large number of agents having all a dynamics of the form (1.1). In this model, the number of agents is so large that at each time only a *statistical (macroscopic) description* of the state is available. We thus describe the configuration of the system at time *t* by a Borel measure $\mu _t$ on ${\mathbb {R}}^d$, where for every Borel set $A \subseteq {\mathbb {R}}^d$ the quotient $\dfrac{\mu _t(A)}{\mu _t({\mathbb {R}}^d)}$ represents the fraction of the total amount of agents that are present in *A* at the time *t*. Since the total amount of agents is supposed to be fixed in time, $\mu _t({\mathbb {R}}^d)$ is constant, and thus, we choose to normalize the measure $\mu _t$ assuming $\mu _t({\mathbb {R}}^d)=1$, i.e., $\mu _t\in {\mathscr {P}}({\mathbb {R}}^d)$, the set of Borel probability measures on ${\mathbb {R}}^d$.

Hence, the evolution of the controlled multi-agent system can be represented by the following two-scale dynamics

- *Microscopic dynamics:* each agent’s position at time *t* is given by *x*(*t*), which evolves according to the dynamical system
  1.2 $$\begin{aligned} \dot{x}(t) \in F(\mu _t,x(t)),&\text{ for } \text{ a.e. } t>0\, , \end{aligned}$$
  where *F* is a set-valued map. It is worth pointing out that each agent’s dynamics is nonlocal since it depends also on the instantaneous configuration $\mu _t$ of the crowd of agents at time *t*, described by a probability measure on ${\mathbb {R}}^d$.
- *Macroscopic dynamics:* the configuration of the crowd of agents at time *t* is given by a time-depending measure $\mu _t\in {\mathscr {P}}({\mathbb {R}}^d)$ whose evolution satisfies the following *continuity equation* (to be understood in the sense of distributions)
  1.3 $$\begin{aligned} \partial _t \mu _t+\mathrm {div}(v_t \mu _t)=0,&t>0, \end{aligned}$$
  coupled with the control constraint
  1.4 $$\begin{aligned} v_t(x) \in F(\mu _t, x) \text{ for } \mu _t\text{-a.e. } x \in {\mathbb {R}}^d \text{ and } \text{ for } \text{ a.e. } t \ge 0. \end{aligned}$$
  which represents the possible (Eulerian) velocity $v_t(x)$ chosen by an external planner for an agent at time *t* and at the position *x*.

The investigation of (deterministic) optimal control problems in the space of measures is attracting an increasing interest by the mathematical community in the last years, due to the potential applications in the study of complex systems, or multi-agent systems (see, e.g., [16, 18, 19]). Indeed, in the framework of *mean field approximation* of multi agent system, starting from a control problem for a large number of the (discrete) agents, the problem is recasted in the framework of probability measures (see the recent [15] or the preprint [12] for $\Gamma$-convergence results for optimal control problems with nonlocal dynamics). This procedure reduces the dimensionality and the number of equations, possibly leading to a simpler and treatable problem from the point of view of numerics. The reader can find a comprehensive overview of the literature about such formulations and applications, together with some insights on research perspective, in the recent survey [1], and references therein. We refer to [7] for further results on mean field control problems.

The problem we address in this paper is the compatibility of the above dynamical system (1.3)–(1.4) with a given closed constraint ${\mathscr {K}}\subseteq {\mathscr {P}}_2({\mathbb {R}}^d)$. Here, ${\mathscr {P}}_2({\mathbb {R}}^d)$ is the set of Borel probability measures with finite second moment; this set is equipped with the 2-Wasserstein distance (see Sect. 2). This compatibility property could be understood in two ways

- ${\mathscr {K}}$ is *viable* for the dynamics *F* if and only if for any $\mu \in {\mathscr {K}}$ *there exists* a solution $t \mapsto \mu _t$ of the controlled continuity Eqs. (1.3)–(1.4) with $\mu _0 =\mu$ such that $\mu _t \in {\mathscr {K}}$ for all $t \ge 0$;
- ${\mathscr {K}}$ is *invariant* for the dynamics *F* if and only if for any $\mu \in {\mathscr {K}}$ and * for any * solution $t \mapsto \mu _t$ of the controlled continuity Eqs. (1.3)–(1.4) with $\mu _0 =\mu$ we have $\mu _t \in {\mathscr {K}}$ for all $t \ge 0$.

Inspired by a characterization of the viability property via supersolution of Hamilton–Jacobi–Bellman equations, which was first obtained in [9] in the framework of stochastic control, we develop an approach for the present multiagent control problem with deterministic dynamics (1.3)–(1.4).

The main result of our paper (Theorems 6.6 and 6.7) can be roughly summarized as follows

### Theorem 1.1

Let ${\mathscr {K}}\subseteq {\mathscr {P}}_2({\mathbb {R}}^d)$ be a closed set and $d_{{\mathscr {K}}}$ the associated distance function. Assume that the set valued map *F* is *L*-Lipschitz.

- ${\mathscr {K}}$ is *viable* iff the function $\mu \mapsto d_{{\mathscr {K}}}(\mu )$ is a viscosity supersolution of
  $$\begin{aligned} (L+2) u(\mu ) +{\mathscr {H}}_F^{\mathrm {viab}}(\mu ,D_\mu u(\mu ))=0, \end{aligned}$$
  where, for all $\mu \in {\mathscr {P}}_2({\mathbb {R}}^d)$, $p\in L^2_\mu ({\mathbb {R}}^d;{\mathbb {R}}^d)$,
  $$\begin{aligned} {\mathscr {H}}_F^{\mathrm {viab}}(\mu ,p):=-d_{{\mathscr {K}}}(\mu )-\mathop {{{\,\mathrm{inf}\,}}}\limits _{\begin{array}{c} v(\cdot )\in L^2_\mu ({\mathbb {R}}^d)\\ v(x)\in F(\mu ,x)\mu -\text { a.e. }x \end{array}}\int _{{\mathbb {R}}^\mathrm{d}}\langle v(x),p(x)\rangle \,\mathrm{d}\mu (x). \end{aligned}$$
- ${\mathscr {K}}$ is *invariant* iff the function $\mu \mapsto d_{{\mathscr {K}}}(\mu )$ is a viscosity supersolution of
  $$\begin{aligned} (L+2) u(\mu ) +{\mathscr {H}}_F^{\mathrm {inv}}(\mu ,D_\mu u(\mu ))=0, \end{aligned}$$
  where, for all $\mu \in {\mathscr {P}}_2({\mathbb {R}}^d)$, $p\in L^2_\mu ({\mathbb {R}}^d;{\mathbb {R}}^d)$,
  $$\begin{aligned}{\mathscr {H}}_F^{\mathrm {inv}}(\mu ,p):=-d_{{\mathscr {K}}}(\mu )-\sup _{\begin{array}{c} v(\cdot )\in L^2_\mu ({\mathbb {R}}^d)\\ v(x)\in F(\mu ,x)\,\mu -\text {a.e.}\,x \end{array}}\int _{{\mathbb {R}}^\mathrm{d}}\langle v(x),p(x)\rangle \,\mathrm{d}\mu (x).\end{aligned}$$

For a completely different approach to the viability problem, we refer to [5], where the author provides a characterization of the viability property for a closed set ${\mathscr {K}}\subseteq {\mathscr {P}}_1({\mathbb {T}}^d)$ by mean of a condition involving a suitable notion of tangent cone to ${\mathscr {K}}$ in the Wasserstein space ${\mathscr {P}}_1({\mathbb {T}}^d)$, where ${\mathbb {T}}^d$ denotes the d-dimensional torus.

The paper is organized as follows: in Sect. 2, we fix the notations and provide some background results; Sect. 3 is devoted to the properties of the set of solutions of the controlled continuity Eqs. (1.3)–(1.4); Sect. 4 establishes the link between the viability/invariance problem with the value function of a suitable control problem in Wasserstein space; Sect. 5 introduces the viscosity solutions of Hamilton–Jacobi–Bellman equations in the Wasserstein space, together with a uniqueness result; in Sect. 6, we apply the results of Sect. 5 to the problem outlined in Sect. 4 deriving our main characterization of viability/invariance. Finally, in Sect. 7 we provide an example illustrating the main results. Some proofs of technical results are postponed to “Appendix.”

## Notations

Throughout the paper, we will use the following notation and we address to [2] as a relevant resource for preliminaries on measure theory.

*B*(*x*, *r*): the open ball of radius *r* of a metric space $(X,d_X)$, i.e., $B(x,r):=\{y\in X:\,d_X(y,x)<r\}$;
$${\overline{K}}$$
the closure of a subset *K* of a topological space *X*;
$$d_K(\cdot )$$
the distance function from a subset *K* of a metric space (*X*, *d*), i.e., $d_K(x):={{\,\mathrm{inf}\,}}\{d(x,y):\,y\in K\}$;
$$C^0_b(X;Y)$$
the set of continuous bounded functions from a Banach space *X* to *Y*, endowed with $\Vert f\Vert _{\infty }=\displaystyle \sup _{x\in X}|f(x)|$ (if $Y={\mathbb {R}}$, *Y* will be omitted);
$$C^0_c(X;Y)$$
the set of compactly supported functions of $C^0_b(X;Y)$, with the topology induced by $C^0_b(X;Y)$;
$$BUC(X;{\mathbb {R}})$$
the space of bounded real-valued uniformly continuous functions defined on *X*
$$\Gamma _I$$
the set of continuous curves from a real interval *I* to ${\mathbb {R}}^d$;
$$\Gamma _T$$
the set of continuous curves from [0, *T*] to ${\mathbb {R}}^d$;
$$e_t$$
the evaluation operator $e_t:{\mathbb {R}}^d\times \Gamma _I\rightarrow {\mathbb {R}}^d$ defined by $e_t(x,\gamma )=\gamma (t)$ for all $t\in I$;
$${\mathscr {P}}(X)$$
the set of Borel probability measures on a Banach space *X*, endowed with the $\hbox {weak}^*$ topology induced from $C^0_b(X)$;
$${\mathscr {M}}({\mathbb {R}}^d;{\mathbb {R}}^d)$$
the set of vector-valued Borel measures on ${\mathbb {R}}^d$ with values in ${\mathbb {R}}^d$, endowed with the $\hbox {weak}^*$ topology induced from $C^0_c({\mathbb {R}}^d;{\mathbb {R}}^d)$;
$$|\nu |$$
the total variation of a measure $\nu \in {\mathscr {M}}({\mathbb {R}}^d;{\mathbb {R}}^d)$;
$$\ll $$
the absolutely continuity relation between measures defined on the same $\sigma$-algebra;
$$\mathrm {m}_2(\mu )$$
the second moment of a probability measure $\mu \in {\mathscr {P}}(X)$;
$$r\sharp \mu $$
the push-forward of the measure $\mu$ by the Borel map *r*;
$$\mu \otimes \pi _x$$
the product measure of $\mu \in {\mathscr {P}}(X)$ with the Borel family of measures $\{\pi _x\}_{x\in X}\subseteq {\mathscr {P}}(Y)$ (see Theorem 2.1);
$$\mathrm {pr}_i$$
the *i*-th projection map $\mathrm {pr}_i(x_1,\dots ,x_N)=x_i$;
$$\Pi (\mu ,\nu )$$
the set of admissible transport plans from $\mu$ to $\nu$;
$$\Pi _o(\mu ,\nu )$$
the set of optimal transport plans from $\mu$ to $\nu$;
$$W_2(\mu ,\nu )$$
the 2-Wasserstein distance between $\mu$ and $\nu$;
$${\mathscr {P}}_2(X)$$
the subset of the elements ${\mathscr {P}}(X)$ with finite second moment, endowed with the 2-Wasserstein distance;
$$\dfrac{\nu }{\mu }$$
the Radon–Nikodym derivative of the measure $\nu$ w.r.t. the measure $\mu$;
$$\mathrm {Lip}(f)$$
the Lipschitz constant of a function *f*;
$$(f)^+$$
the positive part of a real valued function *f*, i.e., $(f)^+=\max \{0,f\}$.

Given Banach spaces *X*, *Y*, we denote by ${\mathscr {P}}(X)$ the set of Borel probability measures on *X* endowed with the $\hbox {weak}^*$ topology induced by the duality with the Banach space $C^0_b(X)$ of the real-valued continuous bounded functions on *X* with the uniform convergence norm. The second moment of $\mu \in {\mathscr {P}}(X)$ is defined by $\displaystyle \mathrm {m}_2(\mu )=\int _{X}\Vert x\Vert _X^2\,\mathrm{d}\mu (x)$, and we set ${\mathscr {P}}_2(X)=\{\mu \in {\mathscr {P}}(X):\, \mathrm {m}_2(\mu )<+\infty \}$. For any Borel map $r:X\rightarrow Y$ and $\mu \in {\mathscr {P}}(X)$, we define the *push forward measure* $r\sharp \mu \in {\mathscr {P}}(Y)$ by setting $r\sharp \mu (B)=\mu (r^{-1}(B))$ for any Borel set *B* of *Y*. In other words,

$$\begin{aligned} \int _Y \varphi (y)\,\mathrm{d}[r\sharp \mu ](y)=\int _X \varphi (r(x))\,\mathrm{d}\mu (x), \end{aligned}$$

for any bounded Borel measurable function $\varphi :Y\rightarrow {\mathbb {R}}$.

We denote by ${\mathscr {M}}(X;Y)$ the set of *Y*-valued Borel measures defined on *X*. The total variation measure of $\nu \in {\mathscr {M}}(X;Y)$ is defined for every Borel set $B\subseteq X$ as

$$\begin{aligned} |\nu |(B):=\sup _{\{B_i\}_{i\in {\mathbb {N}}}}\left\{ \sum \Vert \nu (B_i)\Vert _Y\right\} , \end{aligned}$$

where the sup ranges on countable Borel partitions of *B*.

We now recall the definitions of transport plans and Wasserstein distance (cf. for instance Chapter 6 in [2]). Let *X* be a complete separable Banach space, $\mu _1,\mu _2\in {\mathscr {P}}(X)$. The set of *admissible transport plans* between $\mu _1$ and $\mu _2$ is

$$\begin{aligned} \Pi (\mu _1,\mu _2)=\{\varvec{\pi }\in {\mathscr {P}}(X\times X): \mathrm {pr}_i\sharp {\varvec{\pi }}=\mu _i,\,i=1,2\}, \end{aligned}$$

where for $i=1,2$, $\mathrm {pr}_i:{\mathbb {R}}^d\times {\mathbb {R}}^d\rightarrow {\mathbb {R}}^d$ is a projection $\mathrm {pr}_i(x_1,x_2)=x_i$. The *Wasserstein distance* between $\mu _1$ and $\mu _2$ is

$$\begin{aligned} W_2^2(\mu _1,\mu _2)=\mathop {{{\,\mathrm{inf}\,}}}\limits _{{\varvec{\pi }}\in \Pi (\mu _1,\mu _2)}\int _{X\times X}|x_1-x_2|^2\,\mathrm{d}{\varvec{\pi }}(x_1,x_2). \end{aligned}$$

If $\mu _1,\mu _2\in {\mathscr {P}}_2(X)$, then the above infimum is actually a minimum, and the set of minima is denoted by

$$\begin{aligned}\Pi _o(\mu _1,\mu _2):=\left\{ {\varvec{\pi }}\in \Pi (\mu _1,\mu _2): W_2^2(\mu _1,\mu _2)= \int _{X\times X}|x_1-x_2|^p\,\mathrm{d}{\varvec{\pi }}(x_1,x_2)\right\} .\end{aligned}$$

Recall that ${\mathscr {P}}_2(X)$ endowed with the $W_2$-Wasserstein distance is a complete separable metric space.

The following result is Theorem 5.3.1 in [2].

### Theorem 2.1

(Disintegration) Let ${\mathbb {X}},X$ be complete separable metric spaces. Given a measure $\mu \in {\mathscr {P}}({\mathbb {X}})$ and a Borel map $r:{\mathbb {X}}\rightarrow X$, there exists a Borel family of probability measures $\{\mu _x\}_{x\in X}\subseteq {\mathscr {P}}({\mathbb {X}})$, uniquely defined for $r\sharp \mu$-a.e. $x\in X$, such that $\mu _x({\mathbb {X}}\setminus r^{-1}(x))=0$ for $r\sharp \mu$-a.e. $x\in X$, and for any Borel map $\varphi :{\mathbb {X}}\rightarrow [0,+\infty ]$ we have

$$\begin{aligned}\int _{{\mathbb {X}}}\varphi (z)\,\mathrm{d}\mu (z)=\int _X \left[ \int _{r^{-1}(x)}\varphi (z)\,\mathrm{d}\mu _x(z)\right] \mathrm{d}(r\sharp \mu )(x).\end{aligned}$$

We will write $\mu =(r\sharp \mu )\otimes \mu _x$. If ${\mathbb {X}}=X\times Y$ and $r^{-1}(x)\subseteq \{x\}\times Y$ for all $x\in X$, we can identify each measure $\mu _x\in {\mathscr {P}}(X\times Y)$ with a measure on *Y*.

## Admissible trajectories

The goal of this section is to give a precise definition of the macroscopic dynamics (1.3 , 1.4) and to study its trajectories. To maintain the flow of the paper, the proofs of the results of this section are postponed to “Appendix A.”

### Definition 3.1

(*Admissible trajectories*) Let $I=[a,b]$ be a closed real interval, ${\varvec{\mu }}=\{\mu _t\}_{t\in I}\subseteq {\mathscr {P}}_2({\mathbb {R}}^d)$, ${\varvec{\nu }}=\{\nu _t\}_{t\in I}\subseteq {\mathscr {M}}({\mathbb {R}}^d;{\mathbb {R}}^d)$, $F:{\mathscr {P}}_2({\mathbb {R}}^d)\times {\mathbb {R}}^d \rightrightarrows {\mathbb {R}}^d$ be a set-valued map.

We say that ${\varvec{\mu }}$ is an *admissible trajectory driven by* ${\varvec{\nu }}$ defined on *I* with underlying dynamics *F* if

- $$|\nu _t|\ll \mu _t$$ for a.e. $$t\in I$$;
- $$v_t(x):=\dfrac{\nu _t}{\mu _t}(x)\in F(\mu _t,x)$$ for a.e. $$t\in I$$ and $$\mu _t$$-a.e. $$x\in {\mathbb {R}}^d$$;
- $\partial _t\mu _t+\mathrm {div}\,\nu _t=0$ in the sense of distributions in $[a,b]\times {\mathbb {R}}^d$.

Given $\mu \in {\mathscr {P}}_2({\mathbb {R}}^d)$, we define the set of *admissible trajectories* as

$$\begin{aligned} {\mathcal {A}}_I(\mu )&:=\Big \{{\varvec{\mu }}=\{\mu _t\}_{t\in I}\,:\,\exists \, {\varvec{\nu }}=\{\nu _t\}_{t\in I}\subseteq {\mathscr {M}}({\mathbb {R}}^d;{\mathbb {R}}^d) \text{ s.t. } {\varvec{\mu }} \text{ is } \text{ an } \text{ admissible } \text{ traj. }\\ {}&\qquad \qquad \text{ driven } \text{ by } {\varvec{\nu }}, \text{ defined } \text{ on } I \text{ with } \text{ underlying } \text{ dynamics } F \text{ and } \mu _{a}=\mu \Big \}. \end{aligned}$$

We make the following assumptions on the set-valued map *F*:

$$({\varvec{F}}_1)$$
$F:{\mathscr {P}}_2({\mathbb {R}}^d)\times {\mathbb {R}}^d\rightrightarrows {\mathbb {R}}^d$ is continuous with convex, compact and nonempty images, where on ${\mathscr {P}}_2({\mathbb {R}}^d)\times {\mathbb {R}}^d$ we consider the metric
  $$\begin{aligned}d_{{\mathscr {P}}_2({\mathbb {R}}^d)\times {\mathbb {R}}^d}((\mu _1,x_1),(\mu _2,x_2))=W_2(\mu _1,\mu _2)+|x_1-x_2|.\end{aligned}$$
$$({\varvec{F}}_2)$$
there exists $L>0$, a compact metric space *U* and a continuous map $f:{\mathscr {P}}_2({\mathbb {R}}^d)\times {\mathbb {R}}^d\times U\rightarrow {\mathbb {R}}^d$ satisfying
  $$\begin{aligned}|f(\mu _1,x_1,u)-f(\mu _2,x_2,u)|\le L (W_2(\mu _1,\mu _2)+|x_1-x_2|),\end{aligned}$$
  for all $\mu _i\in {\mathscr {P}}_2({\mathbb {R}}^d)$, $x_i\in {\mathbb {R}}^d$, $i=1,2$, $u\in U$, such that the set-valued map *F* can be represented as
  $$\begin{aligned}F(\mu ,x)=\left\{ f(\mu ,x,u):u\in U\right\} .\end{aligned}$$

As pointed out also in Remark 2 of [16], from the Lipschitz continuity of the set-valued map *F* coming from assumption $({\varvec{F}}_2)$, we easily get

$$\begin{aligned}F(\mu ,x)\subseteq F(\nu ,y)+L (W_2(\mu ,\nu )+|x-y|)\overline{B(0,1)},\end{aligned}$$

for all $\mu ,\nu \in {\mathscr {P}}_2({\mathbb {R}}^d)$ and $x,y\in {\mathbb {R}}^d$. From which, for all $\mu \in {\mathscr {P}}_2({\mathbb {R}}^d)$ and $x\in {\mathbb {R}}^d$, we have

3.1 $$\begin{aligned} F(\mu ,x)\subseteq C(1+\mathrm {m}_2^{1/2}(\mu ))\,(1+|x|)\overline{B(0,1)}, \end{aligned}$$

where $$C:=\max \{1,L\,\max \{|y|\,:\,y\in F(\delta _0,0)\}\}$$.

### Definition 3.2

Let $\{\varvec{\mu }^{(n)}\}_{n\in {\mathbb {N}}}\subseteq \mathrm {AC}([a,b];{\mathscr {P}}_2({\mathbb {R}}^d))$. We say that $\{{\varvec{\mu }}^{(n)}\}_{n\in {\mathbb {N}}}$ *uniformly* $W_2$*-converges* to ${\varvec{\mu }}$, $\varvec{\mu }^{(n)}\rightrightarrows {\varvec{\mu }}$, if we have

$$\begin{aligned} \sup _{t\in [a,b]}W_2(\mu ^{(n)}_t,\mu _t)\rightarrow 0. \end{aligned}$$

We recall the following result taken from [16].

### Lemma 3.3

(Grönwall-like estimate (Prop. 2 in [16])) Assume $\varvec{(F_1)-(F_2)}$. Let $\mu _0,\theta _0\in {\mathscr {P}}_2({\mathbb {R}}^d)$, and $\varvec{\mu }=\{\mu _t\}_{t\in [a,b]}\in {\mathcal {A}}_{[a,b]}(\mu _0)$ an admissible trajectory. Then, there exists an admissible trajectory $\varvec{\theta }=\{\theta _t\}_{t\in [a,b]}\in {\mathcal {A}}_{[a,b]}(\theta _0)$, such that for all $t\in [a,b]$ we have

$$\begin{aligned}W_2(\mu _t,\theta _t)\le e^{L(b-a)+(b-a)e^{L(b-a)}}\cdot W_2(\mu _0,\theta _0),\end{aligned}$$

where *L* is as in $\varvec{(F_2)}$.

### Proposition 3.4

Assume $\varvec{(F_1)-(F_2)}$. Let ${\varvec{\mu }}=\{\mu _t\}_{t\in [a,b]}$ be an admissible trajectory, with $0\le a< b<+\infty$. Then, there exists ${\varvec{\eta }}\in {\mathscr {P}}({\mathbb {R}}^d\times \Gamma _{[a,b]})$ such that $e_t\sharp {\varvec{\eta }}=\mu _t$ for all $t\in [a,b]$, and for ${\varvec{\eta }}$-a.e. $(x,\gamma )$

$$\begin{aligned} {\left\{ \begin{array}{ll} {\dot{\gamma }}(t)\in F(\mu _t,\gamma (t)),&{}\text {for a.e. }t\in [a,b],\\ \gamma (a)=x. \end{array}\right. } \end{aligned}$$

Moreover, for any ${\varvec{\eta }}$ as above and for all $t,s\in [a,b]$ with $s<t$, we have

1. for $${\varvec{\eta }}$$-a.e. $$(x,\gamma )\in {\mathbb {R}}^d\times \Gamma _{[a,b]}$$,
  $$\begin{aligned}&\dfrac{e_t-e_s}{t-s}(x,\gamma )\in F(\mu _s,\gamma (s))+\\&\qquad +\left[ \dfrac{L}{t-s}\int _s^t\left[ W_2(\mu _\tau ,\mu _s)+|(e_\tau -e_s)(x,\gamma )|\right] \,\mathrm {d}\tau \right] \cdot \overline{B(0,1)}; \end{aligned}$$
2. $$\Vert e_t-e_s\Vert _{L^2_{{\varvec{\eta }}}}\le e^{L(t-s)}\left[ (t-s)(K+2L \mathrm {m}^{1/2}_2(\mu _s))+L\displaystyle \int _s^tW_2(\mu _\tau ,\mu _s)\,\mathrm{d}\tau \right] =:h(t,s)$$;
3. $$\lim _{t\rightarrow s^+}\left\| \dfrac{e_t-e_s}{t-s}\right\| _{L^2_{{\varvec{\eta }}}}=K+2 L\mathrm {m}_2^{1/2}(\mu _s)$$,

where $$L=\mathrm {Lip}(F)$$ and $$K=\max \{|y|:\,y\in F(\delta _0,0)\}$$.

In particular, there exists a Borel map $w:{\mathbb {R}}^d\times \Gamma _{[a,b]}\rightarrow {\mathbb {R}}^d$, with $w(x,\gamma )\in F(\mu _s,\gamma (s))$ for ${\varvec{\eta }}$-a.e. $(x,\gamma )\in {\mathbb {R}}^d\times \Gamma _{[a,b]}$, such that

$$\begin{aligned}\left\| \dfrac{e_t-e_s}{t-s}-w\right\| _{L^2_{{\varvec{\eta }}}}\le \dfrac{L}{t-s}\int _s^t\left[ W_2(\mu _\tau ,\mu _s)+h(\tau ,s)\right] \,\mathrm{d}\tau .\end{aligned}$$

### Proposition 3.5

(Compactness of ${\mathcal {A}}_{[a,b)}(\mu )$] Assume $({\varvec{F}}_1)-({\varvec{F}}_2)$ and let $0\le a<b<+\infty$ and $\mu _0\in {\mathscr {P}}_2({\mathbb {R}}^d)$. Then, the set of admissible trajectories ${\mathcal {A}}_{[a,b]}(\mu _0)$ is nonempty and compact w.r.t. uniform $W_2$-convergence (see Definition 3.2).

## Viability problem and the value function

Throughout the paper, let ${\mathscr {K}}\subseteq {\mathscr {P}}_2({\mathbb {R}}^d)$ be closed w.r.t. the metric $W_2$. We are interested in the definitions of compatibility of our dynamics w.r.t. the state constraint given by ${\mathscr {K}}$ (cf. introduction of the present paper).

Notice that, since concatenation of admissible trajectories is an admissible trajectory (see the note before Prop. 3 in [16]), if ${\mathscr {K}}$ is viable (resp. invariant) in $[t_0,T]$ then it is viable (resp. invariant) in $[0,{\hat{T}}]$ for any ${\hat{T}}>T$.

As we will investigate in Sect. 5, the viability and invariance properties of a closed set ${\mathscr {K}}\subseteq {\mathscr {P}}_2({\mathbb {R}}^d)$ are closely related to the following optimal control problems, with fixed time-horizon $T>0$.

### Definition 4.1

(*Value functions*) Given ${\mathscr {K}}\subseteq {\mathscr {P}}_2({\mathbb {R}}^d)$ closed, $\mu \in {\mathscr {P}}_2({\mathbb {R}}^d)$ and $t_0\in [0,T]$ , we set

1. $$V^{\mathrm {viab}}:[0,T]\times {\mathscr {P}}_2({\mathbb {R}}^d)\rightarrow [0,+\infty )$$ as follows
  4.1 $$\begin{aligned} V^{\mathrm {viab}}(t_0,\mu ):=\mathop {{{\,\mathrm{inf}\,}}}\limits _{{\varvec{\mu }}\in {\mathcal {A}}_{[t_0,T]}(\mu )}\int _{t_0}^T d_{{\mathscr {K}}}(\mu _t)\,\mathrm{d}t, \end{aligned}$$
  where $d_{{\mathscr {K}}}:{\mathscr {P}}_2({\mathbb {R}}^d)\rightarrow [0,+\infty )$, $d_{{\mathscr {K}}}(\mu ):=\mathop {{{\,\mathrm{inf}\,}}}\limits _{\sigma \in {\mathscr {K}}} W_2(\mu ,\sigma )$.We say that ${\varvec{\mu }}\in {\mathcal {A}}_{[t_0,T]}(\mu )$ is an *optimal trajectory for* $V^{\mathrm {viab}}$ starting from $\mu$ at time $t_0$ if it achieves the minimum in (4.1).
2. $$V^{\mathrm {inv}}:[0,T]\times {\mathscr {P}}_2({\mathbb {R}}^d)\rightarrow [0,+\infty )$$ as follows
  4.2 $$\begin{aligned} V^{\mathrm {inv}}(t_0,\mu ):=\sup _{{\varvec{\mu }}\in {\mathcal {A}}_{[t_0,T]}(\mu )}\int _{t_0}^T d_{{\mathscr {K}}}(\mu _t)\,\mathrm{d}t. \end{aligned}$$
  We say that ${\varvec{\mu }}\in {\mathcal {A}}_{[t_0,T]}(\mu )$ is an *optimal trajectory for *$V^{\mathrm {inv}}$ starting from $\mu$ at time $t_0$ if it achieves the maximum in (4.2).

The main interest in the above value functions lies in the fact that they give a characterization of the viability/invariance as explained in Proposition 4.3. We first state a regularity result of the above value functions and the existence of optimal trajectories.

### Proposition 4.2

Assume $\varvec{(F_1)-(F_2)}$. Given $\mu \in {\mathscr {P}}_2({\mathbb {R}}^d)$, $t_0\in [0,T]$, there exist an *optimal trajectory* ${\varvec{\mu }}\in {\mathcal {A}}_{[t_0,T]}(\mu )$ *for *$V^{\mathrm {viab}}$ and an *optimal trajectory* ${\varvec{\mu }}'\in {\mathcal {A}}_{[t_0,T]}(\mu )$ *for *$V^{\mathrm {inv}}$.

### Proof

We prove the existence of an optimal trajectory for $V^{\mathrm {viab}}$. Take any $\mu ^1,\mu ^2\in {\mathscr {P}}_2({\mathbb {R}}^d)$. By passing to the infimum over $\sigma \in {\mathscr {K}}$ on the triangular inequality

$$\begin{aligned}W_2(\mu ^1,\sigma )\le W_2(\mu ^1,\mu ^2)+W_2(\mu ^2,\sigma ),\end{aligned}$$

we have $d_{{\mathscr {K}}}(\mu ^1)\le W_2(\mu ^1,\mu ^2)+d_{{\mathscr {K}}}(\mu ^2)$. Reversing the roles of $\mu ^1$ and $\mu ^2$, we get the 1-Lipschitz continuity of $d_{{\mathscr {K}}}$. Hence, by Fatou’s Lemma, we get the l.s.c. of the cost functional, i.e.,

$$\begin{aligned}\int _{t_0}^Td_{{\mathscr {K}}}(\mu _t)\,\mathrm{d}t\le \liminf _{n\rightarrow +\infty }\int _{t_0}^Td_{{\mathscr {K}}}(\mu _t^{(n)})\,\mathrm{d}t,\end{aligned}$$

for any sequence $\{{\varvec{\mu }}^{(n)}\}_{n\in {\mathbb {N}}}\subseteq \mathrm {AC}([t_0,T];{\mathscr {P}}_2({\mathbb {R}}^d))$ uniformly $W_2$-converging to ${\varvec{\mu }}$. Combining this with the $W_2$-compactness property of Proposition 3.5, we get the desired result.

We prove the existence of an optimal trajectory for $V^{\mathrm {inv}}$. We fix $\{{\varvec{\mu }}^{(n)}\}_{n\in {\mathbb {N}}}\subset {\mathcal {A}}_{[t_0,T]}(\mu )$ and ${\hat{\sigma }}\in {\mathscr {K}}$. For any $t\in [t_0,T]$, by triangular inequality and recalling that by definition we have the equivalence $\mathrm {m}_2^{1/2}(\rho )=W_2(\rho ,\delta _0)$, we get the following uniform bound

$$\begin{aligned} d_{{\mathscr {K}}}(\mu _t^{(n)})&\le W_2(\mu _t^{(n)},{\hat{\sigma }})\le W_2(\mu _t^{(n)},\delta _0)+W_2(\delta _0,{\hat{\sigma }})\\&=\mathrm {m}_2^{1/2}(\mu _t^{(n)})+\mathrm {m}_2^{1/2}({\hat{\sigma }})\le {\tilde{C}}(1+\mathrm {m}_2^{1/2}(\mu ))+\mathrm {m}_2^{1/2}({\hat{\sigma }}), \end{aligned}$$

for some constant ${\tilde{C}}>0$ coming from estimate (A.2) proved in “Appendix A”. Thus, as for the proof of the existence of a minimizer for $V^{\mathrm {viab}}$, we can apply Fatou’s Lemma to get the u.s.c. of the cost functional and conclude. $\square$

We state here a first characterization of viability/invariance in terms of the optimal control problems introduced in Definition 4.1.

### Proposition 4.3

Assume $\varvec{(F_1)-(F_2)}$. Let ${\mathscr {K}}\subseteq {\mathscr {P}}_2({\mathbb {R}}^d)$ be closed in the $W_2$-topology, $t_0\in [0,T]$. Then,

1. ${\mathscr {K}}$ is *viable* for *F* if and only if $V^{\mathrm {viab}}(t_0,\mu _0)=0$ for all $\mu _0\in {\mathscr {K}}$;
2. ${\mathscr {K}}$ is *invariant* for *F* if and only if $V^{\mathrm {inv}}(t_0,\mu _0)=0$ for all $\mu _0\in {\mathscr {K}}$.

### Proof

We just prove (1), since the proof of (2) is similar. One implication follows directly by definition, so we prove the other direction assuming $V^{\mathrm {viab}}(t_0,\mu _0)=0$ for all $\mu _0\in {\mathscr {K}}$. By Proposition 4.2, for all $\mu _0\in {\mathscr {K}}$, there exists an optimal trajectory $\varvec{{\bar{\mu }}}\in {\mathcal {A}}_{[t_0,T]}(\mu _0)$ such that

$$\begin{aligned} 0=V^{\mathrm {viab}}(t_0,\mu _0)=\int _{t_0}^Td_{{\mathscr {K}}}({\bar{\mu }}_t)\,\mathrm{d}t. \end{aligned}$$

This implies that $d_{{\mathscr {K}}}({\bar{\mu }}_t)=0$ for a.e. $t\in [t_0,T]$. By continuity of $\varvec{{\bar{\mu }}}$ and by closedness of ${\mathscr {K}}$ w.r.t. $W_2$-topology, we obtain the viability property for ${\mathscr {K}}$. $\square$

As usual, the value function satisfies a Dynamic Programming Principle.

### Lemma 4.4

(DPP) The function $V^{\mathrm {viab}}:[0,T]\times {\mathscr {P}}_2({\mathbb {R}}^d)\rightarrow [0,+\infty )$ satisfies

4.3 $$\begin{aligned} V^{\mathrm {viab}}(t_0,\mu )={{\,\mathrm{inf}\,}}\left\{ \int _{t_0}^t d_{{\mathscr {K}}}(\mu _s)\,\mathrm{d}s+V^{\mathrm {viab}}(t,\mu _t):\, t\in [t_0,T],\, {\varvec{\mu }}\in {\mathcal {A}}_{[t_0,T]}(\mu )\right\} .\nonumber \\ \end{aligned}$$

Furthermore, for any ${\varvec{\mu }}\in {\mathcal {A}}_{[t_0,T]}(\mu )$, the map

$$\begin{aligned}t\mapsto g_{{\varvec{\mu }}}(t):=\int _{t_0}^t d_{{\mathscr {K}}}(\mu _s)\,\mathrm{d}s+V^{\mathrm {viab}}(t,\mu _t)\end{aligned}$$

is nondecreasing in $[t_0,T]$, and it is constant if and only if ${\varvec{\mu }}$ is an optimal trajectory.

### Proof

We prove one inequality ($\ge$). By definition of $V^{\mathrm {viab}}(t_0,\mu )$, for any $\varepsilon >0$ there exists ${\varvec{\mu }}^\varepsilon \in {\mathcal {A}}_{[t_0,T]}(\mu )$ s.t.

$$\begin{aligned} \begin{aligned}&V^{\mathrm {viab}}(t_0,\mu )+\varepsilon \ge \int _{t_0}^t d_{{\mathscr {K}}}(\mu ^\varepsilon _s)\,\mathrm {d}s+\int _t^T d_{{\mathscr {K}}}(\mu ^\varepsilon _s)\,\mathrm {d}s\ge \int _{t_0}^t d_{{\mathscr {K}}}(\mu ^\varepsilon _s)\,\mathrm {d}s+V^{\mathrm {viab}}(t,\mu ^\varepsilon _t), \end{aligned} \end{aligned}$$

for any $t\in [t_0,T]$, since the truncated trajectory $\varvec{{\hat{\mu }}}:={\varvec{\mu }}^\varepsilon _{|[t,T]}$ belongs to ${\mathcal {A}}_{[t,T]}(\mu ^\varepsilon _t)$. We conclude by passing to the infimum on ${\varvec{\mu }}\in {\mathcal {A}}_{[t_0,T]}(\mu )$ and $t\in [t_0,T]$ on the right-hand side and then letting $\varepsilon \rightarrow 0^+$.

Concerning the other inequality, fix any ${\varvec{\mu }}\in {\mathcal {A}}_{[t_0,T]}(\mu )$ and $t\in [t_0,T]$. By definition of $V^{\mathrm {viab}}(t,\mu _t)$, for all $\varepsilon >0$ there exists ${\varvec{\mu }}^\varepsilon \in {\mathcal {A}}_{[t,T]}(\mu _t)$ s.t. $V^{\mathrm {viab}}(t,\mu _t)+\varepsilon \ge \int _t^T d_{{\mathscr {K}}}(\mu ^\varepsilon _s)\,\mathrm{d}s$. Now, defining

$$\begin{aligned} {\hat{\mu }}_s:={\left\{ \begin{array}{ll} \mu _s,&{}\text {if }s\in [t_0,t],\\ \mu _s^\varepsilon ,&{}\text {if }s\in [t,T], \end{array}\right. } \end{aligned}$$

we see that $\varvec{{\hat{\mu }}}\in {\mathcal {A}}_{[t_0,T]}(\mu )$. Thus,

$$\begin{aligned} V^{\mathrm {viab}}(t_0,\mu )\le & {} \int _{t_0}^T d_{{\mathscr {K}}}({\hat{\mu }}_s)\,\mathrm{d}s=\int _{t_0}^t d_{{\mathscr {K}}}(\mu _s)\,\mathrm{d}s+\int _t^T d_{{\mathscr {K}}}(\mu ^\varepsilon _s)\,\mathrm{d}s \le \int _{t_0}^t d_{{\mathscr {K}}}(\mu _s)\,\mathrm{d}s+V^{\mathrm {viab}}(t,\mu _t)+\varepsilon . \end{aligned}$$

By passing to the ${{\,\mathrm{inf}\,}}$ on ${\varvec{\mu }}\in {\mathcal {A}}_{[t_0,T]}(\mu )$, and then letting $\varepsilon \rightarrow 0^+$, we conclude.

The proof of the second part of the statement is standard and follows straightforwardly from (4.3) (see for instance Prop. 3 in [16]). $\square$

We come now to the formulation of a Dynamic Programming Principle for the value function $V^{\mathrm {inv}}$ whose proof is omitted since it is similar to that of Lemma 4.4.

### Lemma 4.5

(DPP) The function $V^{\mathrm {inv}}:[0,T]\times {\mathscr {P}}_2({\mathbb {R}}^d)\rightarrow [0,+\infty )$ satisfies

4.4 $$\begin{aligned} V^{\mathrm {inv}}(t_0,\mu )=\sup \left\{ \int _{t_0}^t d_{{\mathscr {K}}}(\mu _s)\,\mathrm{d}s+V^{\mathrm {inv}}(t,\mu _t):\, t\in [t_0,T],\, {\varvec{\mu }}\in {\mathcal {A}}_{[t_0,T]}(\mu )\right\} .\nonumber \\ \end{aligned}$$

Furthermore, for any ${\varvec{\mu }}\in {\mathcal {A}}_{[t_0,T]}(\mu )$, the map

$$\begin{aligned}t\mapsto j_{{\varvec{\mu }}}(t):=\int _{t_0}^t d_{{\mathscr {K}}}(\mu _s)\,\mathrm{d}s+V^{\mathrm {inv}}(t,\mu _t)\end{aligned}$$

is nonincreasing in $[t_0,T]$, and it is constant if and only if ${\varvec{\mu }}$ is an optimal trajectory.

As in the classical case, the infinitesimal version of the Dynamic Programming Principle gives rise to a Hamilton–Jacobi–Bellman equation. The next section is devoted to such a Hamilton–Jacobi equation.

### Proposition 4.6

Assume $\varvec{(F_1)-(F_2)}$. The value functions $V^{\mathrm {viab}}(t,\mu )$ and $V^{\mathrm {inv}}(t,\mu )$ are uniformly continuous in $t\in [0,T]$ and Lipschitz continuous in $\mu \in {\mathscr {P}}_2({\mathbb {R}}^d)$ w.r.t. the $W_2$-metric.

### Proof

We prove the statement for $V^{\mathrm {viab}}$, since the proof for $V^{\mathrm {inv}}$ is analogous. Fix $t_0\in [0,T]$ and take any $\mu ^1,\mu ^2\in {\mathscr {P}}_2({\mathbb {R}}^d)$. By Proposition 4.2, there exists an optimal trajectory $\varvec{{\bar{\mu }}}^2\in {\mathcal {A}}_{[t_0,T]}(\mu ^2)$ starting from $\mu ^2$. Thus, for any admissible ${\varvec{\mu }}^1\in {\mathcal {A}}_{[t_0,T]}(\mu ^1)$, we have

$$\begin{aligned} \left| V^{\mathrm {viab}}(t_0,\mu ^1)-V^{\mathrm {viab}}(t_0,\mu ^2)\right| \le \int _{t_0}^T \left| d_{{\mathscr {K}}}(\mu ^1_t)-d_{{\mathscr {K}}}({\bar{\mu }}^2_t)\right| \,\mathrm{d}t\le \int _{t_0}^T W_2(\mu ^1_t,{\bar{\mu }}^2_t)\,\mathrm{d}t. \end{aligned}$$

We can now choose ${\varvec{\mu }}^1\in {\mathcal {A}}_{[t_0,T]}(\mu ^1)$ such that the Grönwall-like inequality of Lemma 3.3 holds, thus getting

4.5 $$\begin{aligned} \left| V^{\mathrm {viab}}(t_0,\mu ^1)-V^{\mathrm {viab}}(t_0,\mu ^2)\right| \le (T-t_0) e^{L(T-t_0)+(T-t_0)e^{L(T-t_0)}}\cdot W_2(\mu ^1,\mu ^2).\nonumber \\ \end{aligned}$$

We now prove the uniform continuity in time of $V^{\mathrm {viab}}$. Let $0\le t_1\le t_2\le T$, $\mu \in {\mathscr {P}}_2({\mathbb {R}}^d)$ and ${\varvec{\mu }}\in {\mathcal {A}}_{[t_1,T]}(\mu )$ an optimal trajectory. Then by the second part of the statement of Lemma 4.4, noticing that in particular $g_{{\varvec{\mu }}}(t_1)=V^{\mathrm {viab}}(t_1,\mu )$, we have

$$\begin{aligned} V^{\mathrm {viab}}(t_1,\mu )-V^{\mathrm {viab}}(t_2,\mu )&=\int _{t_1}^{t_2}d_{{\mathscr {K}}}(\mu _t)\,\mathrm{d}t+V^{\mathrm {viab}}(t_2,\mu _{|t=t_2})-V(t_2,\mu )\\&\le \int _{t_1}^{t_2}d_{{\mathscr {K}}}(\mu _t)\,\mathrm{d}t+T e^{LT+Te^{LT}}W_2(\mu _{|t=t_2},\mu ). \end{aligned}$$

By continuity of $d_{{\mathscr {K}}}(\cdot )$ and of $t\mapsto \mu _t$ we have the convergence to zero of the right-hand-side as $t_2\rightarrow t_1$. Reversing the roles of $t_1$ and $t_2$ we conclude. $\square$

## Hamilton Jacobi Bellman equation

As reported in p. 352 in [11] and at the beginning of Sec. 6.1 in [10], we recall the following crucial fact. Throughout the paper, let $(\Omega ,{\mathcal {B}},{\mathbb {P}})$ be a sufficiently “rich” probability space, i.e., $\Omega$ is a complete, separable metric space, ${\mathcal {B}}$ is the Borel $\sigma$-algebra on $\Omega$, and ${\mathbb {P}}$ is an atomless Borel probability measure. We use the notation $L^2_{{\mathbb {P}}}(\Omega )=L^2_{{\mathbb {P}}}(\Omega ;{\mathbb {R}}^d)$. Then, given any $\mu _1,\mu _2\in {\mathscr {P}}_2({\mathbb {R}}^d)$, there exist $X_1,X_2\in L^2_{{\mathbb {P}}}(\Omega )$ such that $\mu _i=X_i\sharp {\mathbb {P}}$, $i=1,2$, and $W_2(\mu _1,\mu _2)=\Vert X_1-X_2\Vert _{L^2_{{\mathbb {P}}}}$.

### Definition 5.1

1. Given a function $u:[0,T]\times {\mathscr {P}}_2({\mathbb {R}}^d)\rightarrow {\mathbb {R}}$, we define its *lift* $U:[0,T]\times L^2_{{\mathbb {P}}}(\Omega )\rightarrow {\mathbb {R}}$ by setting $U(t,X)=u(t,X\sharp {\mathbb {P}})$ for all $X\in L^2_{{\mathbb {P}}}(\Omega )$.
2. Let ${\mathscr {H}}={\mathscr {H}}(\mu ,p)$ be a Hamiltonian function mapping $\mu \in {\mathscr {P}}_2({\mathbb {R}}^d)$, $p\in L^2_\mu ({\mathbb {R}}^d)$ into ${\mathbb {R}}$. We say that the Hamiltonian function $H:L^2_{{\mathbb {P}}}(\Omega )\times L^2_{{\mathbb {P}}}(\Omega )\rightarrow {\mathbb {R}}$ is a *lift* of ${\mathscr {H}}$, if $H(X,p\circ X)={\mathscr {H}}(X\sharp {\mathbb {P}},p)$, for all $X\in L^2_{{\mathbb {P}}}(\Omega )$, $p\in L^2_{X\sharp {\mathbb {P}}}({\mathbb {R}}^d)$.

### Definition 5.2

(*Viscosity solution*) Let ${\mathscr {H}}$ and *H* be as in Definition 5.1(2). Given $\lambda \ge 0$, we consider a first-order HJB equation of the form

5.1 $$\begin{aligned} -\partial _t u(t,\mu )+\lambda u(t,\mu )+{\mathscr {H}}(\mu ,D_\mu u(t,\mu ))=0, \end{aligned}$$

and its lifted form

5.2 $$\begin{aligned} -\partial _t U(t,X)+\lambda U(t,X)+H(X,DU(t,X))=0. \end{aligned}$$

We say that $u:[0,T]\times {\mathscr {P}}_2({\mathbb {R}}^d)\rightarrow {\mathbb {R}}$ is a viscosity subsolution (resp. supersolution) of (5.1) in $[0,T)\times {\mathscr {P}}_2({\mathbb {R}}^d)$ if and only if its lift is a viscosity subsolution (resp. supersolution) of (5.2) in $[0,T)\times L^2_{{\mathbb {P}}}(\Omega )$. We recall that $U:[0,T]\times L^2_{{\mathbb {P}}}(\Omega )\rightarrow {\mathbb {R}}$ is a

- viscosity subsolution of (5.2) if for any test function $\phi \in C^1([0,T]\times L^2_{{\mathbb {P}}}(\Omega ))$ such that $U-\phi$ has a local maximum at $(t_0,X_0)\in [0,T)\times L^2_{{\mathbb {P}}}(\Omega )$ it holds $-\partial _t \phi (t_0,X_0)+\lambda U(t_0,X_0)+H(X_0,D\phi (t_0,X_0))\le 0$;
- viscosity supersolution of (5.2) if for any test function $\phi \in C^1([0,T]\times L^2_{{\mathbb {P}}}(\Omega ))$ such that $U-\phi$ has a local minimum at $(t_0,X_0)\in [0,T)\times L^2_{{\mathbb {P}}}(\Omega )$ it holds $-\partial _t \phi (t_0,X_0)+\lambda U(t_0,X_0)+H(X_0,D\phi (t_0,X_0))\ge 0$;
- viscosity solution of (5.2) if it is both a supersolution and a subsolution.

### Remark 5.3

Assume $u:[0,T]\times {\mathscr {P}}_2({\mathbb {R}}^d)\rightarrow {\mathbb {R}}$ is constant in time, i.e., with slight abuse of notation we can identify $u(t,\mu )=u(\mu )$ for any $(t,\mu )\in [0,T]\times {\mathscr {P}}_2({\mathbb {R}}^d)$, with $u:{\mathscr {P}}_2({\mathbb {R}}^d)\rightarrow {\mathbb {R}}$. Then, (5.1) and (5.2) become, respectively

5.3 $$\begin{aligned} \lambda u(\mu )+{\mathscr {H}}(\mu ,D_\mu u(\mu ))=0, \quad \lambda U(X)+H(X,DU(X))=0, \end{aligned}$$

where $U:L^2_{{\mathbb {P}}}(\Omega )\rightarrow {\mathbb {R}}$ is the lift of *u*. Moreover, the test functions in Definition 5.2 can be taken independent of *t*, i.e.,

- *U* is a viscosity subsolution of (5.3) if for any test function $\phi \in C^1(L^2_{{\mathbb {P}}}(\Omega ))$ such that $U-\phi$ has a local maximum at $X_0\in L^2_{{\mathbb {P}}}(\Omega )$ it holds $\lambda U(X_0)+H(X_0,D\phi (X_0))\le 0$;
- *U* is a viscosity supersolution of (5.3) if for any test function $\phi \in C^1(L^2_{{\mathbb {P}}}(\Omega ))$ such that $U-\phi$ has a local minimum at $X_0\in L^2_{{\mathbb {P}}}(\Omega )$ it holds $\lambda U(X_0)+H(X_0,D\phi (X_0))\ge 0$.
- *U* is a viscosity solution of (5.3) if it is both a supersolution and a subsolution.

### Theorem 5.4

(Comparison principle) Assume that there exists $L,C>0$ such that the Hamiltonian function $H:L^2_{{\mathbb {P}}}(\Omega )\times L^2_{{\mathbb {P}}}(\Omega )\rightarrow {\mathbb {R}}$ satisfies the following assumption:

$$\varvec{(H)}$$
for any $X,Y\in L^2_{{\mathbb {P}}}(\Omega )$, any $a,b_1,b_2>0$ and $C_1,C_2\in L^2_{{\mathbb {P}}}(\Omega )$,
  $$\begin{aligned} \begin{aligned}&H(Y,a(X-Y)-b_1 Y-C_1)-H(X,a(X-Y)+b_2 X+C_2)\\ {}&\le \Vert X-Y\Vert _{L^2_{{\mathbb {P}}}}+2aL\Vert X-Y\Vert ^2_{L^2_{{\mathbb {P}}}}+\\ {}&\quad +C(1+\mathrm {m}_2^{1/2}(Y\sharp {\mathbb {P}}))\,(1+\Vert Y\Vert _{L^2_{{\mathbb {P}}}})\,(\Vert C_1\Vert _{L^2_{{\mathbb {P}}}}+b_1\Vert Y\Vert _{L^2_{{\mathbb {P}}}})+\\ {}&\quad +C(1+\mathrm {m}_2^{1/2}(X\sharp {\mathbb {P}}))\,(1+\Vert X\Vert _{L^2_{{\mathbb {P}}}})\,(\Vert C_2\Vert _{L^2_{{\mathbb {P}}}}+b_2\Vert X\Vert _{L^2_{{\mathbb {P}}}}). \end{aligned} \end{aligned}$$

Let $\lambda \ge 0$. Then, if $u_1,u_2\in UC([0,T]\times {\mathscr {P}}_2({\mathbb {R}}^d))$ are a subsolution and a supersolution of (5.1), respectively, we have

5.4 $$\begin{aligned} \sup _{[0,T]\times {\mathscr {P}}_2({\mathbb {R}}^d)}(u_1-u_2)\le \sup _{\{T\}\times {\mathscr {P}}_2({\mathbb {R}}^d)}(u_1-u_2)^+. \end{aligned}$$

### Proof

The proof follows the line of the corresponding classical finite-dimensional argument (see, e.g., Theorem II.2.12 p. 107) in [6]. In the following, we define ${\mathbb {G}}:={\mathbb {R}}\times L^2_{{\mathbb {P}}}(\Omega )$ and, for any $(t,X)\in {\mathbb {G}}$, we set $\Vert (t,X)\Vert ^2_{{\mathbb {G}}}:=|t|^2+\Vert X\Vert ^2_{L^2_{{\mathbb {P}}}}$. We denote ${\mathbb {A}}:=[0,T]\times L^2_{{\mathbb {P}}}(\Omega )\subset {\mathbb {G}}$, that is a complete metric space with distance induced by the norm $\Vert \cdot \Vert _{{\mathbb {G}}}$ of ${\mathbb {G}}$.

Let $U_1,U_2:{\mathbb {A}}\rightarrow {\mathbb {R}}$ be, respectively, the lift functionals for $u_1$ and $u_2$ as in Definition 5.1(1). We define the functional $\Phi :{\mathbb {A}}^2\rightarrow {\mathbb {R}}$ by setting

$$\begin{aligned} \Phi (t,X,s,Y):=&U_1(t,X)-U_2(s,Y)-\frac{\Vert (t,X)-(s,Y)\Vert ^2_{{\mathbb {G}}}}{2\varepsilon }+\\&\quad -\beta \left( (1+\Vert X\Vert ^2_{L^2_{{\mathbb {P}}}})^{m/2}+(1+\Vert Y\Vert ^2_{L^2_{{\mathbb {P}}}})^{m/2}\right) +\eta (t+s), \end{aligned}$$

where $\varepsilon ,\beta ,m,\eta >0$ are positive constants which will be chosen later. Notice that since $u_i\in UC([0,T]\times {\mathscr {P}}_2({\mathbb {R}}^d))$, $i=1,2$, we have $U_i\in UC([0,T]\times L^2_{{\mathbb {P}}}(\Omega ))$. Indeed, for all $X,Y\in L^2_{{\mathbb {P}}}(\Omega )$, $t,s\in [0,T]$,

$$\begin{aligned} |U_i(t,X)-U_i(s,Y)|&=|u_i(t,X\sharp {\mathbb {P}})-u_i(s,Y\sharp {\mathbb {P}})| \le \omega _{u_i}\left( \sqrt{|t-s|^2+W^2_2(X\sharp {\mathbb {P}},Y\sharp {\mathbb {P}})}\right) \\&\le \omega _{u_i}\left( \Vert (t,X)-(s,Y)\Vert _{{\mathbb {G}}}\right) , \end{aligned}$$

where $\omega _{u_i}(\cdot )$ is the modulus of continuity of $u_i$ and where we used the fact that $W_2(X\sharp {\mathbb {P}},Y\sharp {\mathbb {P}})\le \Vert X-Y\Vert _{L^2_{{\mathbb {P}}}}$. Set

$$\begin{aligned}A:=\sup _{\{T\}\times {\mathscr {P}}_2({\mathbb {R}}^d)}(u_1-u_2)^+=\sup _{\{T\}\times L^2_{{\mathbb {P}}}}(U_1-U_2)^+.\end{aligned}$$

For $$R'>0$$, $$i=1,2$$, set

$$\begin{aligned}\varrho _i(R'):=\sup \{|U_i(t,X)-U_i(s,Y)|\,:\,\Vert (t,X)-(s,Y)\Vert _{{\mathbb {G}}}\le R'\};\end{aligned}$$

by uniform continuity we have

5.5 $$\begin{aligned} \sup _{R'\ge 0}\frac{\varrho _i(R')}{1+R'}<+\infty . \end{aligned}$$

Thus,

$$\begin{aligned} U_1(t,X)-U_2(s,Y)&=U_1(t,X)-U_1(T,X)+U_1(T,X)-U_2(T,X)\\&\quad +U_2(T,X)-U_2(s,Y)\\&\le \varrho _1(T-t)+A+\varrho _2(\Vert (T,X)-(s,Y)\Vert _{{\mathbb {G}}}) \end{aligned}$$

for all $(t,X,s,Y)\in {\mathbb {A}}^2$. By (5.5), there exists ${\mathscr {C}}>0$ such that

5.6 $$\begin{aligned} |U_1(t,X)-U_2(s,Y)|\le {\mathscr {C}}(1+\Vert X-Y\Vert _{L^2_{{\mathbb {P}}}}),\quad \text {for all }(t,X,s,Y)\in {\mathbb {A}}^2. \end{aligned}$$

The proof proceeds by contradiction: assume that there exist $({\tilde{t}},{\tilde{\mu }})\in [0,T]\times {\mathscr {P}}_2({\mathbb {R}}^d)$ and $\delta >0$ such that $u_1({\tilde{t}},{\tilde{\mu }})-u_2({\tilde{t}},{\tilde{\mu }})=A+\delta$. In particular, for any ${\tilde{X}}\in L^2_{{\mathbb {P}}}(\Omega )$ such that ${\tilde{X}}\sharp {\mathbb {P}}={\tilde{\mu }}$, it holds $U_1({\tilde{t}},{\tilde{X}})-U_2({\tilde{t}},{\tilde{X}})=A+\delta$.

Select $\beta ,\eta >0$ such that

$$\begin{aligned}A+\dfrac{\delta }{2}\le A+\delta -2\beta (1+\Vert {\tilde{X}}\Vert ^2_{L^2_{{\mathbb {P}}}})^{m/2}+2\eta {\tilde{t}}=\Phi ({\tilde{t}},{\tilde{X}},{\tilde{t}},{\tilde{X}})\le \sup _{{\mathbb {A}}^2}\Phi .\end{aligned}$$

Noting that $\Phi \in C^0({\mathbb {A}}^2)$, by taking $\varepsilon <\frac{1}{2{\mathscr {C}}}$ and recalling (5.6), we have

$$\begin{aligned}\lim _{\begin{array}{c} \Vert X\Vert _{L^2_{{\mathbb {P}}}}\rightarrow +\infty \\ \Vert Y\Vert _{L^2_{{\mathbb {P}}}}\rightarrow +\infty \end{array}}\Phi (t,X,s,Y)=-\infty ,\end{aligned}$$

for any $t,s\in [0,T]$. Therefore, there exists $R>0$ such that

$$\begin{aligned}\sup _{{\mathbb {A}}^2}\Phi (t,X,s,Y)=\sup _{([0,T]\times \overline{B_{L^2_{{\mathbb {P}}}}(0,R)})^2}\Phi (t,X,s,Y).\end{aligned}$$

Thus, by Stegall’s Variational Principle (see, e.g., Theorem 6.3.5 in [8]) for any fixed $\xi >0$, there exists a linear and continuous functional $\Lambda :{\mathbb {G}}^2\rightarrow {\mathbb {R}}$ with $\Vert \Lambda \Vert _{{{\mathbb {G}}^2}^*}<\xi$ and such that $\Phi -\Lambda$ attains a strong maximum in $([0,T]\times \overline{B_{L^2_{{\mathbb {P}}}}(0,R)})^2$. Moreover, on $([0,T]\times \overline{B_{L^2_{{\mathbb {P}}}}(0,R)})^2$, we have

$$\begin{aligned}\Phi (t,X,s,Y)-\Lambda (t,X,s,Y)\ge \Phi (t,X,s,Y)-2\xi \sqrt{T^2+R^2},\end{aligned}$$

and so

5.7 $$\begin{aligned} \sup _{{\mathbb {A}}^2}\Phi \le 2\xi \sqrt{T^2+R^2}+\sup _{([0,T]\times \overline{B_{L^2_{{\mathbb {P}}}}(0,R)})^2}(\Phi -\Lambda ). \end{aligned}$$

Let $({\bar{t}},{\bar{X}},{\bar{s}},{\bar{Y}})\in ([0,T]\times \overline{B_{L^2_{{\mathbb {P}}}}(0,R)})^2$ be a maximizer of $\Phi -\Lambda$ on $([0,T]\times \overline{B_{L^2_{{\mathbb {P}}}}(0,R)})^2$, obtained by choosing $\xi >0$ s.t. $2\xi \sqrt{T^2+R^2}\le \frac{\delta }{8}$. In particular, we get

$$\begin{aligned} A+\frac{\delta }{2}&\le \sup _{{\mathbb {A}}^2}\Phi \le 2\xi \sqrt{T^2+R^2}+(\Phi -\Lambda )({\bar{t}},{\bar{X}},{\bar{s}},{\bar{Y}})\le \frac{\delta }{8}+(\Phi -\Lambda )({\bar{t}},{\bar{X}},{\bar{s}},{\bar{Y}})\\&\le \frac{\delta }{8}+\Phi ({\bar{t}},{\bar{X}},{\bar{s}},{\bar{Y}})+2\xi \sqrt{T^2+R^2}\le \frac{\delta }{4}+\Phi ({\bar{t}},{\bar{X}},{\bar{s}},{\bar{Y}}), \end{aligned}$$

and so

5.8 $$\begin{aligned} \Phi ({\bar{t}},{\bar{X}},{\bar{s}},{\bar{Y}})\ge A+\frac{\delta }{4}, \end{aligned}$$

leading to

$$\begin{aligned} \beta \left( (1+\Vert {\bar{X}}\Vert ^2_{L^2_{{\mathbb {P}}}})^{m/2}+(1+\Vert {\bar{Y}}\Vert ^2_{L^2_{{\mathbb {P}}}})^{m/2}\right) \le \sup U_1-{{\,\mathrm{inf}\,}}U_2-A-\frac{\delta }{4}+\eta ({\bar{t}}+{\bar{s}}). \end{aligned}$$

By choosing $0<\eta <1$, we get for all $\varepsilon >0$, $m\in (0,1]$

5.9 $$\begin{aligned}&\beta ((1+\Vert {\bar{X}}\Vert ^2_{L^2_{{\mathbb {P}}}})^{m/2}+(1+\Vert {\bar{Y}}\Vert ^2_{L^2_{{\mathbb {P}}}})^{m/2}) \le \sup U_1-{{\,\mathrm{inf}\,}}U_2-A-\frac{\delta }{4}+2T =:d>0.\nonumber \\ \end{aligned}$$

By Riesz’ representation theorem, there exist unique $(\lambda _1,\lambda _2,\lambda _3,\lambda _4)\in {\mathbb {G}}^2$ such that

$$\begin{aligned}\Lambda (t,X,s,Y)=\lambda _1 t+\langle \lambda _2,X\rangle _{L^2_{{\mathbb {P}}}}+\lambda _3 s+\langle \lambda _4,Y\rangle _{L^2_{{\mathbb {P}}}}.\end{aligned}$$

From (5.7), we have

$$\begin{aligned} \Phi ({\bar{t}},{\bar{X}},{\bar{t}},{\bar{X}})+\Phi ({\bar{s}},{\bar{Y}},{\bar{s}},{\bar{Y}})&\le 2\,(\Phi -\Lambda )({\bar{t}},{\bar{X}},{\bar{s}},{\bar{Y}})+4\xi \sqrt{T^2+R^2}\\&\le 2\Phi ({\bar{t}},{\bar{X}},{\bar{s}},{\bar{Y}})+8\xi \sqrt{T^2+R^2}, \end{aligned}$$

and so

$$\begin{aligned}&U_1({\bar{t}},{\bar{X}})-U_2({\bar{t}},{\bar{X}})+U_1({\bar{s}}, {\bar{Y}})-U_2({\bar{s}},{\bar{Y}})+\\&\qquad -2\beta \left( (1+\Vert {\bar{X}}\Vert ^2_{L^2_{{\mathbb {P}}}})^{m/2}+(1+\Vert {\bar{Y}}\Vert ^2_{L^2_{{\mathbb {P}}}})^{m/2}\right) +2\eta ({\bar{t}}+{\bar{s}})\\&\qquad \le 2 U_1({\bar{t}},{\bar{X}})-2U_2({\bar{s}},{\bar{Y}})-\frac{\Vert ({\bar{t}},{\bar{X}})-({\bar{s}},{\bar{Y}})\Vert ^2_{{\mathbb {G}}}}{\varepsilon } -2\beta \left( (1+\Vert {\bar{X}}\Vert ^2_{L^2_{{\mathbb {P}}}})^{m/2} +(1+\Vert {\bar{Y}}\Vert ^2_{L^2_{{\mathbb {P}}}})^{m/2}\right) +\\&\qquad +2\eta ({\bar{t}}+{\bar{s}})+8\xi \sqrt{T^2+R^2}, \end{aligned}$$

which leads to

5.10 $$\begin{aligned} \frac{\Vert ({\bar{t}},{\bar{X}})-({\bar{s}},{\bar{Y}})\Vert ^2_{{\mathbb {G}}}}{\varepsilon }\le & {} U_1({\bar{t}},{\bar{X}})-U_1({\bar{s}},{\bar{Y}}) +U_2({\bar{t}},{\bar{X}})-U_2({\bar{s}},{\bar{Y}})\nonumber \\&+8\xi \sqrt{T^2+R^2}. \end{aligned}$$

Take $0<\xi<\varepsilon <1$. From the previous inequality, the boundedness of $U_1, U_2$ in $[0,T]\times \overline{B_{L^2_{{\mathbb {P}}}}(0,R)}$ gives

5.11 $$\begin{aligned} \Vert ({\bar{t}},{\bar{X}})-({\bar{s}},{\bar{Y}})\Vert _{{\mathbb {G}}}\le B'\sqrt{\varepsilon }+8\varepsilon \sqrt{T^2+R^2}\le B\sqrt{\varepsilon }, \end{aligned}$$

for suitable constants $B',B>0$ independent on $\varepsilon$.

By uniform continuity of $U_i$, $i=1,2$, and by plugging the previous relation in (5.10), we can build a modulus of continuity $\omega (\cdot )$ such that

5.12 $$\begin{aligned} \frac{\Vert ({\bar{t}},{\bar{X}})-({\bar{s}},{\bar{Y}})\Vert ^2_{{\mathbb {G}}}}{\varepsilon }\le \omega (\varepsilon ):=\omega _{u_1}(B\sqrt{\varepsilon })+\omega _{u_2}(B\sqrt{\varepsilon })+8\varepsilon \sqrt{T^2+R^2}. \end{aligned}$$

We show that neither ${\bar{t}}$ nor ${\bar{s}}$ can be equal to *T*. Indeed, in ${\bar{t}}=T$,

$$\begin{aligned} \Phi (T,{\bar{X}},{\bar{s}},{\bar{Y}})&\le U_1(T,{\bar{X}})-U_2(T,{\bar{X}})+U_2(T,{\bar{X}})-U_2({\bar{s}},{\bar{Y}})+\eta (T+{\bar{s}})\\&\le A+\omega _{u_2}(B\sqrt{\varepsilon })+2\eta T, \end{aligned}$$

by definition of *A*. We thus get a contradiction with (5.8) by choosing $\varepsilon$ and $\eta$ small enough s.t. $\omega _{u_2}(B\sqrt{\varepsilon })+2\eta T<\frac{\delta }{4}$. The same reasoning applies for proving ${\bar{s}}<T$.

We define the $C^1({\mathbb {A}})$ test functions

$$\begin{aligned} \begin{aligned} \phi (t,X)&:=U_2({\bar{s}},{\bar{Y}})+\frac{\Vert (t, X)-({\bar{s}},{\bar{Y}})\Vert ^2_{{\mathbb {G}}}}{2\varepsilon }+\\ {}&\quad +\beta \left( (1+\Vert X\Vert ^2_{L^2_{{\mathbb {P}}}})^{m/2}+(1+\Vert {\bar{Y}}\Vert ^2_{L^2_{{\mathbb {P}}}})^{m/2}\right) -\eta (t+{\bar{s}})+\\ {}&\quad +\Lambda (t,X,{\bar{s}},{\bar{Y}}),\\ \psi (s,Y)&:=U_1({\bar{t}},{\bar{X}})-\frac{\Vert ({\bar{t}}, {\bar{X}})-(s,Y)\Vert ^2_{{\mathbb {G}}}}{2\varepsilon }+\\ {}&\quad -\beta \left( (1+\Vert {\bar{X}}\Vert ^2_{L^2_{{\mathbb {P}}}})^{m/2}+(1+\Vert Y\Vert ^2_{L^2_{{\mathbb {P}}}})^{m/2}\right) +\eta ({\bar{t}}+s)+\\ {}&\quad -\Lambda ({\bar{t}},{\bar{X}},s,Y). \end{aligned} \end{aligned}$$

Notice that $(U_1-\phi )(t,X)=(\Phi -\Lambda )(t,X,{\bar{s}},{\bar{Y}})$, hence, $U_1-\phi$ attains its maximum at $({\bar{t}},{\bar{X}})\in [0,T)\times \overline{B_{L^2_{{\mathbb {P}}}}(0,R)}$ and, similarly, $U_2-\psi$ attains its minimum at $({\bar{s}},{\bar{Y}})\in [0,T)\times \overline{B_{L^2_{{\mathbb {P}}}}(0,R)}$. We have

$$\begin{aligned}&\partial _t\phi ({\bar{t}},{\bar{X}})=\frac{{\bar{t}}-{\bar{s}}}{\varepsilon }-\eta +\lambda _1, \qquad \partial _t\psi ({\bar{s}},{\bar{Y}})=\frac{{\bar{t}}-{\bar{s}}}{\varepsilon }+\eta -\lambda _3,\\&D\phi ({\bar{t}},{\bar{X}})=\frac{{\bar{X}}-{\bar{Y}}}{\varepsilon }+m\beta (1+\Vert {\bar{X}}\Vert ^2_{L^2_{{\mathbb {P}}}})^{\frac{m-2}{2}}{\bar{X}}+\lambda _2,\\&D\psi ({\bar{s}},{\bar{Y}})=\frac{{\bar{X}}-{\bar{Y}}}{\varepsilon }-m\beta (1+\Vert {\bar{Y}}\Vert ^2_{L^2_{{\mathbb {P}}}})^{\frac{m-2}{2}}{\bar{Y}}-\lambda _4. \end{aligned}$$

Since ${\bar{t}},{\bar{s}}\in [0,T)$, by definition of viscosity sub/supersolution, we have

$$\begin{aligned} \begin{aligned}&-\partial _t\phi ({\bar{t}},{\bar{X}})+\lambda U_1({\bar{t}},{\bar{X}})+H({\bar{X}},D\phi ({\bar{t}},{\bar{X}}))\le 0 \le \\ {}&\le -\partial _t\psi ({\bar{s}},{\bar{Y}})+\lambda U_2({\bar{s}},{\bar{Y}})+H({\bar{Y}},D\psi ({\bar{s}},{\bar{Y}})). \end{aligned} \end{aligned}$$

Now, by (5.8), we have

$$\begin{aligned} U_1({\bar{t}},{\bar{X}})-U_2({\bar{s}},{\bar{Y}})&\ge \Phi ({\bar{t}},{\bar{X}},{\bar{s}},{\bar{Y}})-\eta ({\bar{t}}+{\bar{s}})\ge \Phi ({\bar{t}},{\bar{X}},{\bar{s}},{\bar{Y}})-2T\eta \\&\ge A+\frac{\delta }{4}-2T\eta , \end{aligned}$$

and we can choose $\eta$ sufficiently small so that $A+\frac{\delta }{4}-2T\eta \ge 0$. Then, we get

$$\begin{aligned} 2\eta&\le 2\eta +\lambda (U_1({\bar{t}},{\bar{X}})-U_2({\bar{s}},{\bar{Y}}))\\&\le \lambda _1+\lambda _3+H({\bar{Y}},D\psi ({\bar{s}},{\bar{Y}}))-H({\bar{X}},D\phi ({\bar{t}},{\bar{X}})). \end{aligned}$$

We can now invoke assumption $\varvec{(H)}$ with

$$\begin{aligned}&a=\frac{1}{\varepsilon },\quad b_1=m\beta (1+\Vert {\bar{Y}}\Vert ^2_{L^2_{{\mathbb {P}}}})^{\frac{m-2}{2}},\quad C_1=\lambda _4,\\&b_2=m\beta (1+\Vert {\bar{X}}\Vert ^2_{L^2_{{\mathbb {P}}}})^{\frac{m-2}{2}},\quad C_2=\lambda _2, \end{aligned}$$

recalling that $\lambda _1,\lambda _3\le \varepsilon$ and $\Vert \lambda _2\Vert _{L^2_{{\mathbb {P}}}},\Vert \lambda _4\Vert _{L^2_{{\mathbb {P}}}}\le \varepsilon$ by the bound on the dual norm of the operator $\Lambda$. We get

$$\begin{aligned} \begin{array}{l} 2\eta \le 2\varepsilon +\Vert {\bar{X}}-{\bar{Y}}\Vert _{L^2_{{\mathbb {P}}}}+2L\frac{\Vert {\bar{X}}-{\bar{Y}}\Vert ^2_{L^2_{{\mathbb {P}}}}}{\varepsilon }+ \Vert \lambda _4\Vert _{L^2_{{\mathbb {P}}}}\, C(1+\mathrm {m}_2^{1/2}({\bar{Y}}\sharp {\mathbb {P}}))\,(1+\Vert {\bar{Y}}\Vert _{L^2_{{\mathbb {P}}}})+\\ \quad +C(1+\mathrm {m}_2^{1/2}({\bar{Y}}\sharp {\mathbb {P}}))\,(1+\Vert {\bar{Y}}\Vert _{L^2_{{\mathbb {P}}}}) \,m\beta (1+\Vert {\bar{Y}}\Vert ^2_{L^2_{{\mathbb {P}}}})^{\frac{m-2}{2}}\Vert {\bar{Y}}\Vert _{L^2_{{\mathbb {P}}}} +\\ \quad +\Vert \lambda _2\Vert _{L^2_{{\mathbb {P}}}}\, C(1+\mathrm {m}_2^{1/2}({\bar{X}}\sharp {\mathbb {P}}))\,(1+\Vert {\bar{X}}\Vert _{L^2_{{\mathbb {P}}}})+\\ \quad +C(1+\mathrm {m}_2^{1/2}({\bar{X}}\sharp {\mathbb {P}}))\,(1+\Vert {\bar{X}}\Vert _{L^2_{{\mathbb {P}}}})\,m\beta (1+\Vert {\bar{X}}\Vert ^2_{L^2_{{\mathbb {P}}}})^{\frac{m-2}{2}}\Vert {\bar{X}}\Vert _{L^2_{{\mathbb {P}}}}. \end{array}\end{aligned}$$

By (5.11), (5.12) and recalling that ${\bar{X}},{\bar{Y}}\in \overline{B_{L^2_{{\mathbb {P}}}}(0,R)}$, we have

$$\begin{aligned} 2\eta&\le 2\varepsilon +B\sqrt{\varepsilon }+2L\,\omega (\varepsilon )+\\&\quad +2\varepsilon \,D (1+R)+D_R m\beta \left( (1+\Vert {\bar{Y}}\Vert ^2_{L^2_{{\mathbb {P}}}})^{\frac{m-2}{2}}+(1+\Vert {\bar{X}}\Vert ^2_{L^2_{{\mathbb {P}}}})^{\frac{m-2}{2}})\right) , \end{aligned}$$

where we defined $D_R:=D\,(1+R) \,R$, where $D:=\max \{ C(1+\mathrm {m}_2^{1/2}({\bar{Y}}\sharp {\mathbb {P}})),\,C(1+\mathrm {m}_2^{1/2}({\bar{X}}\sharp {\mathbb {P}}))\}>0$. Finally, by (5.9) we get

$$\begin{aligned} 2\eta&\le 2\varepsilon +B\sqrt{\varepsilon }+2L\,\omega (\varepsilon )+2\varepsilon \,D (1+R)+D_R m \,d \le K o(1)+\eta , \end{aligned}$$

where for the last passage we choose $m\le \frac{\eta }{D_R d}$, and *o*(1) is a function of $\varepsilon$ going to 0 as $\varepsilon \rightarrow 0^+$. This leads to a contradiction as $\varepsilon \rightarrow 0^+$. $\square$

### Remark 5.5

As highlighted also in Remark 3.8 p. 154 of [6], if $\lambda =0$ in (5.1), we can drop the symbol of the positive part in (5.4) and conclude that

$$\begin{aligned}\sup _{[0,T]\times {\mathscr {P}}_2({\mathbb {R}}^d)}(u_1-u_2)\le \sup _{\{T\}\times {\mathscr {P}}_2({\mathbb {R}}^d)}(u_1-u_2).\end{aligned}$$

## Viscosity characterization of viability and invariance

We now provide the main results of the paper: Theorems 6.6 and 6.7. As pointed out also in Remark 4.2 in [18], by Theorem 8.2.11 in [4], the Hamiltonian ${\mathscr {H}}_F^{\mathrm {viab}}$ defined in Theorem 1.1 satisfies

6.1 $$\begin{aligned} {\mathscr {H}}_F^{\mathrm {viab}}(\mu ,p)=-d_{{\mathscr {K}}}(\mu )-\int _{{\mathbb {R}}^d}\mathop {{{\,\mathrm{inf}\,}}}\limits _{v\in F(\mu ,x)}\langle v,p(x)\rangle \,\mathrm{d}\mu (x). \end{aligned}$$

### Definition 6.1

(*Lifted Hamiltonian for viability*) We define the lifted *Hamiltonian in* $L^2_{{\mathbb {P}}}(\Omega )$ associated with ${\mathscr {H}}_F^{\mathrm {viab}}$

$$\begin{aligned} H_F^{\mathrm {viab}}(X,Q):=-d_{{\mathscr {K}}}(X\sharp {\mathbb {P}})-\mathop {{{\,\mathrm{inf}\,}}}\limits _{\begin{array}{c} v(\cdot )\in L^2_{X\sharp {\mathbb {P}}}({\mathbb {R}}^d)\\ v(x)\in F(X\sharp {\mathbb {P}},x)\\ \text {for}\,X\sharp {\mathbb {P}}-\text {a.e.}\,x \end{array}} \int _{\Omega }\langle v\circ X(\omega ),Q(\omega )\rangle \,\mathrm{d}{\mathbb {P}}(\omega ),\end{aligned}$$

for all $X,Q\in L^2_{{\mathbb {P}}}(\Omega )$. Note that $H_F^{\mathrm {viab}}$ is a lift of ${\mathscr {H}}_F^{\mathrm {viab}}$ according to Definition 5.1.

By disintegrating ${\mathbb {P}}=(X\sharp {\mathbb {P}})\otimes {\mathbb {P}}_x$ (see Theorem 2.1), we have

6.2 $$\begin{aligned} \begin{aligned} H_F^{\mathrm {viab}}(X,Q)&=-d_{{\mathscr {K}}}(X\sharp {\mathbb {P}}) -\mathop {{{\,\mathrm{inf}\,}}}\limits _{\begin{array}{c} v\in L^2_{X\sharp {\mathbb {P}}}({\mathbb {R}}^d)\\ v(x)\in F(X\sharp {\mathbb {P}},x)\\ \text {for}\,X\sharp {\mathbb {P}}-\text {a.e.}\,x \end{array}} \int _{{\mathbb {R}}^d}\int _{X^{-1}(x)}\langle v\circ X(\omega ),Q(\omega )\rangle \, d{\mathbb {P}}_x(\omega )dX\sharp {\mathbb {P}}(x) \\&=-d_{{\mathscr {K}}}(X\sharp {\mathbb {P}})-\mathop {{{\,\mathrm{inf}\,}}}\limits _{\begin{array}{c} v\in L^2_{X\sharp {\mathbb {P}}}({\mathbb {R}}^d)\\ v(x)\in F(X\sharp {\mathbb {P}},x)\\ \text {for}\,X\sharp {\mathbb {P}}-\text {a.e.}\,x \end{array}} \int _{{\mathbb {R}}^d}\langle v(x),\int _{X^{-1}(x)}Q(\omega )d{\mathbb {P}}_x(\omega )\rangle \,\mathrm{d}X\sharp {\mathbb {P}}(x) \\&=-d_{{\mathscr {K}}}(X\sharp {\mathbb {P}})-\int _{{\mathbb {R}}^d}\mathop {{{\,\mathrm{inf}\,}}}\limits _{v\in F(X\sharp {\mathbb {P}},x)}\langle v,\int _{X^{-1}(x)}Q(\omega )d{\mathbb {P}}_x(\omega )\rangle \,\mathrm{d}X\sharp {\mathbb {P}}(x) \\&=-d_{{\mathscr {K}}}(X\sharp {\mathbb {P}})-\int _{{\mathbb {R}}^d}\int _{X^{-1}(x)}\mathop {{{\,\mathrm{inf}\,}}}\limits _{v\in F(X\sharp {\mathbb {P}},X(\omega ))}\langle v,Q(\omega )\rangle \,\mathrm{d}{\mathbb {P}}_x(\omega )dX\sharp {\mathbb {P}}(x) \\&=-d_{{\mathscr {K}}}(X\sharp {\mathbb {P}})-\int _{\Omega }\mathop {{{\,\mathrm{inf}\,}}}\limits _{v\in F(X\sharp {\mathbb {P}},X(\omega ))}\langle v,Q(\omega )\rangle \,\mathrm{d}{\mathbb {P}}(\omega ) \\&=-d_{{\mathscr {K}}}(X\sharp {\mathbb {P}})-\mathop {{{\,\mathrm{inf}\,}}}\limits _{\begin{array}{c} v\in L^2_{{\mathbb {P}}}(\Omega )\\ v(\cdot )\in F(X\sharp {\mathbb {P}},X(\cdot )) \end{array}}\int _{\Omega }\langle v(\omega ),Q(\omega )\rangle \,\mathrm{d}{\mathbb {P}}(\omega ), \end{aligned} \end{aligned}$$

where in the last equality we used Theorem 8.2.11 in [4] (or Theorem 6.31 in [14]).

### Definition 6.2

(*Lifted Hamiltonian for invariance*) Related with the invariance problem and associated with ${\mathscr {H}}_F^{\mathrm {inv}}$, we define the following lifted *Hamiltonian in* $L^2_{{\mathbb {P}}}(\Omega )$

$$\begin{aligned} H_F^{\mathrm {inv}}(X,Q):=-d_{{\mathscr {K}}}(X\sharp {\mathbb {P}})-\sup _{\begin{array}{c} v\in L^2_{X\sharp {\mathbb {P}}}({\mathbb {R}}^d)\\ v(x)\in F(X\sharp {\mathbb {P}},x)\\ \text {for}\,X\sharp {\mathbb {P}}- \text {a.e.}\,x \end{array}} \int _{\Omega }\langle v\circ X(\omega ),Q(\omega )\rangle \,\mathrm{d}{\mathbb {P}}(\omega ),\end{aligned}$$

for all $X,Q\in L^2_{{\mathbb {P}}}(\Omega )$. Notice that $H_F^{\mathrm {inv}}$ is a lift of ${\mathscr {H}}_F^{\mathrm {inv}}$ according to Definition 5.1. Moreover, the equivalences (6.1) and (6.2) hold also in this case replacing, respectively, ${\mathscr {H}}_F^{\mathrm {viab}}$, $H_F^{\mathrm {viab}}$ with ${\mathscr {H}}_F^{\mathrm {inv}}$, $H_F^{\mathrm {inv}}$, and ${{\,\mathrm{inf}\,}}$ with $\sup$.

### Lemma 6.3

Assume $\varvec{(F_1)-(F_2)}$. Then, both the Hamiltonian functions $H_F^{\mathrm {viab}}$ and $H_F^{\mathrm {inv}}$ satisfy assumption $\varvec{(H)}$ with *L* and *C*, respectively, as in $\varvec{(F_2)}$ and (3.1).

### Proof

We prove here the assertion for $H_F^{\mathrm {viab}}$ since the assertion for $H_F^{\mathrm {inv}}$ can be proved in the same way. Fix any $X,Y\in L^2_{{\mathbb {P}}}$, $a,b_1,b_2>0$ and $C_1,C_2\in L^2_{{\mathbb {P}}}$, and denote $\mu _1:=X\sharp {\mathbb {P}}$, $\mu _2:=Y\sharp {\mathbb {P}}$. We have

6.3 $$\begin{aligned} \begin{aligned}&H_F^{\mathrm {viab}}(Y,a(X-Y)-b_1 Y-C_1)-H_F^{\mathrm {viab}}(X,a(X-Y)+b_2 X+C_2)=\\&\quad -d_{{\mathscr {K}}}(\mu _2)-\int _{\Omega }\mathop {{{\,\mathrm{inf}\,}}}\limits _{v\in F(\mu _2,Y(\omega ))}\{a\langle v,X(\omega )-Y(\omega )\rangle -b_1\langle v,Y(\omega )\rangle -\langle v,C_1(\omega )\rangle \}\,\mathrm{d}{\mathbb {P}}\\&\quad +d_{{\mathscr {K}}}(\mu _1)+\int _{\Omega }\mathop {{{\,\mathrm{inf}\,}}}\limits _{w\in F(\mu _1,X(\omega ))} \{a\langle w,X(\omega )-Y(\omega )\rangle +b_2\langle w,X(\omega )\rangle +\langle w,C_2(\omega )\rangle \}\,\mathrm{d}{\mathbb {P}}. \end{aligned} \end{aligned}$$

Let $p\in {\mathbb {R}}^d$. For any $x,y\in {\mathbb {R}}^d$, define $\delta _{x,y}:=L(W_2(\mu _1,\mu _2)+|x-y|)$. Given any $\varepsilon >0$, there exists $z_{\varepsilon ,p}\in F(\mu _1,x)+\delta _{x,y}\overline{B(0,1)}$ such that

$$\begin{aligned} \mathop {{{\,\mathrm{inf}\,}}}\limits _{v\in F(\mu _2,y)}\langle v,p\rangle \ge \mathop {{{\,\mathrm{inf}\,}}}\limits _{z\in F(\mu _1,x)+\delta _{x,y}\overline{B(0,1)}}\langle z,p\rangle \ge \langle z_{\varepsilon ,p},p\rangle - \varepsilon , \end{aligned}$$

where the first inequality comes from Lipschitz continuity of the set-valued map *F*. In particular, we can write $z_{\varepsilon ,p}={\hat{w}}_{\varepsilon ,p}+\delta _{x,y}w_{\varepsilon ,p}$, with ${\hat{w}}_{\varepsilon ,p}\in F(\mu _1,x)$ and $w_{\varepsilon ,p}\in \overline{B(0,1)}$, thus getting

$$\begin{aligned} \mathop {{{\,\mathrm{inf}\,}}}\limits _{v\in F(\mu _2,y)}\langle v,p\rangle&\ge \langle {\hat{w}}_{\varepsilon ,p},p\rangle +\delta _{x,y}\langle w_{\varepsilon ,p},p\rangle - \varepsilon \\&\ge \mathop {{{\,\mathrm{inf}\,}}}\limits _{w\in F(\mu _1,x)}\langle w,p\rangle -\delta _{x,y} |p|-\varepsilon . \end{aligned}$$

Hence, we have

6.4 $$\begin{aligned} \mathop {{{\,\mathrm{inf}\,}}}\limits _{w\in F(\mu _1,x)}\langle w,p\rangle -\mathop {{{\,\mathrm{inf}\,}}}\limits _{v\in F(\mu _2,y)}\langle v,p\rangle \le L(W_2(\mu _1,\mu _2)+|x-y|)\,|p|. \end{aligned}$$

Thus, for any $x,y,c_1, c_2\in {\mathbb {R}}^d$ and by choosing $p=x-y$, it holds

$$\begin{aligned} \begin{aligned}&\mathop {{{\,\mathrm {inf}\,}}}\limits _{w\in F(\mu _1,x)}\left\{ a\langle w,x-y\rangle +b_2\langle w,x\rangle +\langle w,c_2\rangle \right\} + \\ {}&\qquad -\mathop {{{\,\mathrm {inf}\,}}}\limits _{v\in F(\mu _2,y)}\left\{ a\langle v,x-y\rangle -b_1\langle v,y\rangle -\langle v,c_1\rangle \right\} \\ {}&\quad \le a\mathop {{{\,\mathrm {inf}\,}}}\limits _{w\in F(\mu _1,x)}\langle w,x-y\rangle +\sup _{w\in F(\mu _1,x)}\left\{ b_2\langle w,x\rangle +\langle w,c_2\rangle \right\} +\\ {}&\qquad -a\mathop {{{\,\mathrm {inf}\,}}}\limits _{v\in F(\mu _2,y)}\langle v,x-y\rangle +\sup _{v\in F(\mu _2,y)}\left\{ b_1\langle v,y\rangle +\langle v,c_1\rangle \right\} \\ {}&\quad \le a\,L(W_2(\mu _1,\mu _2)+|x-y|)\,|x-y|+\\ {}&\qquad +\sup _{w\in F(\mu _1,x)}\left\{ b_2\langle w,x\rangle +\langle w,c_2\rangle \right\} +\sup _{v\in F(\mu _2,y)}\left\{ b_1\langle v,y\rangle +\langle v,c_1\rangle \right\} \\ {}&\quad \le a\,L(W_2(\mu _1,\mu _2)+|x-y|)\,|x-y|+\\ {}&\qquad +b_2\sup _{w\in F(\mu _1,x)}\langle w,x\rangle +\sup _{w\in F(\mu _1,x)}\langle w,c_2\rangle +b_1\sup _{v\in F(\mu _2,y)}\langle v,y\rangle +\sup _{v\in F(\mu _2,y)}\langle v,c_1\rangle \\ {}&\quad \le a\,L(W_2(\mu _1,\mu _2)+|x-y|)\,|x-y|+\\ {}&\qquad +b_2|x|\sup _{w\in F(\mu _1,x)}|w|+|c_2|\sup _{w\in F(\mu _1,x)}|w|+b_1 |y|\sup _{v\in F(\mu _2,y)}|v|+|c_1|\sup _{v\in F(\mu _2,y)}|v|, \end{aligned} \end{aligned}$$

where we used the Cauchy–Schwarz’s inequality. Integrating with respect to the measure $(X,Y,C_1,C_2)\sharp {\mathbb {P}}$ on the variables $(x,y,c_1,c_2)$ and by (3.1), we get

$$\begin{aligned}&\int _{\Omega }\mathop {{{\,\mathrm{inf}\,}}}\limits _{w\in F(\mu _1,X(\omega ))}\left\{ a\langle w,X(\omega )-Y(\omega )\rangle +b_2\langle w,X(\omega )\rangle +\langle w,C_2(\omega )\rangle \right\} \,\mathrm{d}{\mathbb {P}}+\\&\qquad -\int _{\Omega }\mathop {{{\,\mathrm{inf}\,}}}\limits _{v\in F(\mu _2,Y(\omega ))}\left\{ a\langle v,X(\omega )-Y(\omega )\rangle -b_1\langle v,Y(\omega )\rangle -\langle v,C_1(\omega )\rangle \right\} \,\mathrm{d}{\mathbb {P}}\\&\quad \le 2aL\,\Vert X-Y\Vert ^2_{L^2_{{\mathbb {P}}}}+b_2\,C(1+\mathrm {m}_2^{1/2}(\mu _1))\,(1+\Vert X\Vert _{L^2_{{\mathbb {P}}}})\,\Vert X\Vert _{L^2_{{\mathbb {P}}}}+\\&\qquad +b_1\,C(1+\mathrm {m}_2^{1/2}(\mu _2))\,(1+\Vert Y\Vert _{L^2_{{\mathbb {P}}}})\,\Vert Y\Vert _{L^2_{{\mathbb {P}}}}+\\&\qquad +\Vert C_2\Vert _{L^2_{{\mathbb {P}}}}\,C(1+\mathrm {m}_2^{1/2}(\mu _1))\,(1+\Vert X\Vert _{L^2_{{\mathbb {P}}}})+\Vert C_1\Vert _{L^2_{{\mathbb {P}}}}\,C(1+\mathrm {m}_2^{1/2}(\mu _2))\,(1+\Vert Y\Vert _{L^2_{{\mathbb {P}}}}) \end{aligned}$$

recalling that $$W_2(X\sharp {\mathbb {P}},Y\sharp {\mathbb {P}})\le \Vert X-Y\Vert _{L^2_{{\mathbb {P}}}}$$.

We conclude from (6.3), thanks to the Lipschitz continuity of $d_{{\mathscr {K}}}(\cdot )$. $\square$

### Remark 6.4

Assume $\varvec{(F_1)-(F_2)}$. Let $\mu \in {\mathscr {P}}_2({\mathbb {R}}^d)$ be fixed. Then, the set of continuous selections of $F(\mu ,\cdot )$ is dense in $L^2_{\mu }({\mathbb {R}}^d)$ in the set of Borel selections of $F(\mu ,\cdot )$. Indeed, let $v(\cdot )$ be a Borel selection of $F(\mu ,\cdot )$. By Lusin’s Theorem, for any $\varepsilon >0$ there exists a compact $K_\varepsilon \subseteq {\mathbb {R}}^d$ and a continuous map $w_\varepsilon :{\mathbb {R}}^d\rightarrow {\mathbb {R}}^d$ such that $v=w_\varepsilon$ on $K_\varepsilon$ and $\mu ({\mathbb {R}}^d\setminus K_\varepsilon )<\varepsilon$. By Corollary 9.1.3 in [4], we can extend $w_{\varepsilon |K_\varepsilon }$ to a continuous selection $v_\varepsilon$ of $F(\mu ,\cdot )$. Moreover, we have

$$\begin{aligned} \Vert v_\varepsilon -v\Vert _{L^2_{\mu }}&\le 2\Vert \chi _{{\mathbb {R}}^d\setminus K_\varepsilon }|F(\mu ,\cdot )|\,\Vert _{L^2_{\mu }}. \end{aligned}$$

Since $|F(\mu ,x)|\le |F(\delta _0,0)|+L\mathrm {m}^{1/2}_2(\mu )+L|x|$, we have that

$$\begin{aligned} \Vert v_\varepsilon -v\Vert _{L^2_{\mu }}\le 2\left\| \left( |F(\delta _0,0)|+2L\mathrm {m}^{1/2}_2(\mu )\right) \chi _{{\mathbb {R}}^d\setminus K_\varepsilon }\right\| _{L^2_{\mu }} \le \varepsilon \left( |F(\delta _0,0)|+2L\mathrm {m}^{1/2}_2(\mu )\right) , \end{aligned}$$

and the right hand side tends to 0 as $\varepsilon \rightarrow 0^+$.

Now, we deduce that the value functions $V^{\mathrm {viab}}$ and $V^{\mathrm {inv}}$ satisfy the following Hamilton–Jacobi equations.

### Proposition 6.5

Assume $$\varvec{(F_1)-(F_2)}$$. Then,

1. $V^{\mathrm {viab}}$ is a viscosity solution of
  6.5 $$\begin{aligned} -\partial _t u(t,\mu )+{\mathscr {H}}_F^{\mathrm {viab}}(\mu ,D_\mu u(t,\mu ))=0;\end{aligned}$$
2. $V^{\mathrm {inv}}$ is a viscosity solution of
  6.6 $$\begin{aligned} -\partial _t u(t,\mu )+{\mathscr {H}}_F^{\mathrm {inv}}(\mu ,D_\mu u(t,\mu ))=0.\end{aligned}$$

### Proof

We prove (1). Let $U:[0,T]\times L^2(\Omega ;{\mathbb {R}}^d)\rightarrow {\mathbb {R}}$ be the lift of $V^{\mathrm {viab}}$ according to Definition 5.1.

### Claim 1

*U* is a viscosity supersolution of $-\partial _t U(t,X)+H_F(X,DU(t,X)=0$.

### Proof of Claim 1

Let $\phi :[0,T]\times L^2_{{\mathbb {P}}}(\Omega ;{\mathbb {R}}^d)\rightarrow {\mathbb {R}}$ be a $C^1$ map such that $U-\phi$ attains its minimum at (*s*, *X*), and define $\mu =X\sharp {\mathbb {P}}$. Let ${\varvec{\mu }}=\{\mu _t\}_{t\in [s,T]}$ be an optimal trajectory defined on [*s*, *T*] with $\mu _s=\mu$, its existence being assured by Proposition 4.2, and let ${\varvec{\eta }}\in {\mathscr {P}}({\mathbb {R}}^d\times \Gamma _{[s,T]})$ such that $e_t\sharp {\varvec{\eta }}=\mu _t$ for all $t\in [s,T]$. Fix $\varepsilon >0$ and choose a family $\{Y^\varepsilon _t\}_{t\in [s,T]}\subseteq L^2_{{\mathbb {P}}}(\Omega )$ of random variables satisfying the properties of Corollary A.3 related to ${\varvec{\mu }}$. Then, by the Dynamic Programming Principle in Lemma 4.4 and optimality of ${\varvec{\mu }}$,

$$\begin{aligned} 0=&U(t,Y^\varepsilon _t)-U(s,Y^\varepsilon _s)+\int _s^t d_{\mathscr {K}}(\mu _\tau )\,\mathrm{d}\tau =U(t,Y^\varepsilon _t)-U(s,X)+\int _s^t d_{\mathscr {K}}(\mu _\tau )\,\mathrm{d}\tau \\ \ge&\phi (t,Y^\varepsilon _t)-\phi (s,X)+\int _s^t d_{{\mathscr {K}}}(\mu _\tau )\,\mathrm{d}\tau , \end{aligned}$$

where the equality $U(s,Y^\varepsilon _s)=U(s,X)$ holds since $Y^\varepsilon _s\sharp {\mathbb {P}}=X\sharp {\mathbb {P}}=\mu$ and since *U*, as a lift, is law dependent. Therefore, there exists a continuous increasing function $\varrho :[0,+\infty [\rightarrow [0,+\infty [$ with $\varrho (k)/k\rightarrow 0$ as $k\rightarrow 0^+$ such that we have

$$\begin{aligned} 0\ge&\phi (t,Y^\varepsilon _t)-\phi (s,X)+\int _s^t d_{\mathscr {K}}(\mu _\tau )\,\mathrm{d}\tau \\ \ge&\partial _t\phi (s,X)(t-s)+\langle D\phi (s,X),Y^\varepsilon _t-X\rangle _{L^2_{{\mathbb {P}}}}+\int _s^t d_{{\mathscr {K}}}(\mu _\tau )\,\mathrm{d}\tau +\\&-\varrho \left( |t-s|\left( 1+\left\| \dfrac{Y^\varepsilon _t-Y^\varepsilon _s}{t-s}\right\| _{L^2_{\mathbb P}}\right) +\varepsilon \right) \\ \ge&\partial _t\phi (s,X)(t-s)+\langle D\phi (s,X),Y^\varepsilon _t-Y^\varepsilon _s\rangle _{L^2_{\mathbb P}}+\int _s^t d_{\mathscr {K}}(\mu _\tau )\,\mathrm{d}\tau -\varepsilon \Vert D\phi (s,X)\Vert _{L^2_{{\mathbb {P}}}}\\&-\varrho \left( |t-s|\left( 1+\left\| \dfrac{e_t-e_s}{t-s}\right\| _{L^2_{{\varvec{\eta }}}}\right) +\varepsilon \right) . \end{aligned}$$

Dividing by $t-s>0$, by Corollary A.3(3), we have

$$\begin{aligned} 0\ge&\partial _t\phi (s,X)+\int _{\Omega }\mathop {{{\,\mathrm{inf}\,}}}\limits _{v\in F(X\sharp \mathbb P,X(\omega ))}\langle D\phi (s,X),v\rangle \,\mathrm{d}\mathbb P(\omega )+\dfrac{1}{t-s}\int _s^t d_{{\mathscr {K}}}(\mu _\tau )\,\mathrm{d}\tau +\\&-\dfrac{\varepsilon }{t-s}\Vert D\phi (s,X)\Vert _{L^2_{\mathbb P}}-\dfrac{1}{t-s}\varrho \left( |t-s|\left( 1+\left\| \dfrac{e_t-e_s}{t-s}\right\| _{L^2_{{\varvec{\eta }}}}\right) +\varepsilon \right) +\\&-(\widehat{\varpi }(t)+L\varepsilon )\Vert D\phi (s,X)\Vert _{L^2_{{\mathbb {P}}}}. \end{aligned}$$

By letting $\varepsilon \rightarrow 0^+$, we obtain

$$\begin{aligned} 0\ge&\partial _t\phi (s,X)+\int _{\Omega }\mathop {{{\,\mathrm{inf}\,}}}\limits _{v\in F(X\sharp \mathbb P,X(\omega ))}\langle D\phi (s,X),v\rangle \,\mathrm{d}\mathbb P(\omega )+\dfrac{1}{t-s}\int _s^t d_{{\mathscr {K}}}(\mu _\tau )\,\mathrm{d}\tau +\\&-\dfrac{1}{t-s}\varrho \left( |t-s|\left( 1+\left\| \dfrac{e_t-e_s}{t-s}\right\| _{L^2_{{\varvec{\eta }}}}\right) \right) -\widehat{\varpi }(t)\cdot \Vert D\phi (s,X)\Vert _{L^2_{\mathbb P}}. \end{aligned}$$

Recalling the boundedness of $\left\| \dfrac{e_t-e_s}{t-s}\right\| _{L^2_{{\varvec{\eta }}}}$ coming from Proposition 3.4, by letting $t\rightarrow s^+$, we have

$$\begin{aligned} 0\ge&\partial _t\phi (s,X)+\int _{\Omega }\mathop {{{\,\mathrm{inf}\,}}}\limits _{v\in F(X\sharp \mathbb P,X(\omega ))}\langle D\phi (s,X),v\rangle \,\mathrm{d}{\mathbb {P}}(\omega )+d_{\mathscr {K}}(\mu _s), \end{aligned}$$

i.e., $-\partial _t\phi (s,X)+H_F^{\mathrm {viab}}(X,D\phi (s,X))\ge 0$, where, as already discussed, we have

$$\begin{aligned}H_F^{\mathrm {viab}}(X,Q)=-d_{{\mathscr {K}}}(X\sharp {\mathbb {P}})-\int _{\Omega } \mathop {{{\,\mathrm{inf}\,}}}\limits _{v\in F(X\sharp {\mathbb {P}},X(\omega ))}\langle Q(\omega ),v\rangle \,\mathrm{d}\mathbb P(\omega ). \end{aligned}$$

Thus, *U* is a viscosity supersolution of $-\partial _t U(t,X)+H_F^{\mathrm {viab}}(X,DU(t,X))=0$.

### Claim 2

*U* is a viscosity subsolution of $-\partial _t U(t,X)+H_F^{\mathrm {viab}}(X,DU(t,X)=0$.

### Proof of Claim 2

Let $\phi :[0,T]\times L^2_{{\mathbb {P}}}(\Omega ;{\mathbb {R}}^d)\rightarrow {\mathbb {R}}$ be a $C^1$ map such that $U-\phi$ attains its maximum at (*s*, *X*) and define $\mu =X\sharp {\mathbb {P}}$. Fix $\varepsilon >0$, and let $v_\varepsilon \in L^2_{\mu }({\mathbb {R}}^d)$ be such that $v_\varepsilon (x)\in F(\mu ,x)$ for $\mu$-a.e. $x\in {\mathbb {R}}^d$ and

$$\begin{aligned}&\int _{\Omega } \langle D\phi (s,X)(\omega ),v_\varepsilon \circ X(\omega )\rangle \,\mathrm{d}{\mathbb {P}}(\omega ) \le \mathop {{{\,\mathrm{inf}\,}}}\limits _{\begin{array}{c} v\in L^2_{X\sharp {\mathbb {P}}}({\mathbb {R}}^d)\\ v(\cdot )\in F(X\sharp {\mathbb {P}},\cdot ) \end{array}}\int _{\Omega }\langle D\phi (s,X)(\omega ),v\circ X(\omega )\rangle \,\mathrm{d}{\mathbb {P}}(\omega )+\dfrac{\varepsilon }{2}. \end{aligned}$$

By Remark 6.4, we can suppose that $v_\varepsilon \in C^0$, and by Lemma A.4 there exists an admissible trajectory $\varvec{\mu ^\varepsilon }=\{\mu ^\varepsilon _t\}_{t\in [s,T]}$ defined on [*s*, *T*] with $\mu ^\varepsilon _s=\mu$, and $\varvec{\eta ^\varepsilon }\in {\mathscr {P}}({\mathbb {R}}^d\times \Gamma _{[s,T]})$ such that $e_t\sharp \varvec{\eta ^\varepsilon }=\mu ^\varepsilon _t$ for all $t\in [s,T]$ and

$$\begin{aligned}\lim _{t\rightarrow s^+}\left\| \dfrac{e_t-e_s}{t-s}-v_\varepsilon \circ e_s\right\| _{L^2_{\varvec{\eta ^\varepsilon }}}=0.\end{aligned}$$

By density, we can find ${\hat{v}}_\varepsilon \in C^0_b({\mathbb {R}}^d)$ such that $\Vert v_\varepsilon -{\hat{v}}_\varepsilon \Vert _{L^2_{\mu }}\le \varepsilon$.

Denote by ${\mathscr {V}}_\varepsilon :\Omega \rightarrow {\mathbb {R}}^d\times \Gamma _{[s,T]}$ a Borel map satisfying $\varvec{\eta ^\varepsilon }={\mathscr {V}}_\varepsilon \sharp {\mathbb {P}}$. Recalling Lemma A.2, since for all $\varepsilon >0$ we have $\mu =\mu ^\varepsilon _s=e_s\sharp \varvec{\eta ^\varepsilon }=(e_s\circ {\mathscr {V}}_\varepsilon )\sharp {\mathbb {P}}=X\sharp {\mathbb {P}}$, we can find a sequence of measure-preserving Borel maps $\{r^\varepsilon _{n}(\cdot )\}_{n\in {\mathbb {N}}}$ such that

$$\begin{aligned}{\mathbb {P}}\left( \left\{ \omega \in \Omega :\,|X(\omega )-e_s\circ {\mathscr {V}}_\varepsilon \circ r^\varepsilon _{n}(\omega )|\le \dfrac{1}{n}\right\} \right) =1,\end{aligned}$$

and we set $Y^{\varepsilon ,n}_t=e_t\circ {\mathscr {V}}_\varepsilon \circ r^\varepsilon _{n}$ for all $t\in [s,T]$. In particular, $Y^{\varepsilon ,n}_t\sharp {\mathbb {P}}=\mu ^\varepsilon _t$ for all $t\in [s,T]$. We then have

$$\begin{aligned}\lim _{t\rightarrow s^+}\left\| \dfrac{Y^{\varepsilon ,n}_t-Y^{\varepsilon ,n}_s}{t-s}-v_\varepsilon \circ Y^{\varepsilon ,n}_s\right\| _{L^2_{{\mathbb {P}}}} =\lim _{t\rightarrow s^+}\left\| \dfrac{e_t-e_s}{t-s}-v_\varepsilon \circ e_s\right\| _{L^2_{\varvec{\eta ^\varepsilon }}}=0.\end{aligned}$$

Recalling the choice of ${\hat{v}}_\varepsilon$, we have also

$$\begin{aligned}\Vert v_\varepsilon \circ X-{\hat{v}}_\varepsilon \circ X\Vert _{L^2_{{\mathbb {P}}}}=\Vert v_\varepsilon \circ Y^{\varepsilon ,n}_s-{\hat{v}}_\varepsilon \circ Y^{\varepsilon ,n}_s\Vert _{L^2_{{\mathbb {P}}}}=\Vert v_\varepsilon -{\hat{v}}_\varepsilon \Vert _{L^2_\mu }\le \varepsilon .\end{aligned}$$

Since, by Lemma A.2, $\Vert Y^{\varepsilon ,n}_s-X\Vert _{L^2_{{\mathbb {P}}}}\le \frac{1}{n}$, we can find a subsequence $\{Y^{\varepsilon ,n_h}_s\}_{h\in {\mathbb {N}}}$ such that for ${\mathbb {P}}$-a.e. $\omega \in \Omega$ it holds $\lim _{h\rightarrow +\infty }Y^{\varepsilon ,n_h}_s(\omega )=X(\omega )$. Therefore,

6.7 $$\begin{aligned} \lim _{h\rightarrow +\infty }\int |{\hat{v}}_\varepsilon \circ Y^{\varepsilon ,n_h}_s(\omega )-{\hat{v}}_\varepsilon \circ X(\omega )|^2\,\mathrm{d}{\mathbb {P}}(\omega )=0, \end{aligned}$$

where we used the Dominated Convergence Theorem to pass to the limit under the integral sign, exploiting the global boundedness of ${\hat{v}}_\varepsilon$.

From the Dynamic Programming Principle, for all $t\in [s,T]$ we have

$$\begin{aligned} 0&\le U(t,Y^{\varepsilon ,n_h}_t)-U(s,Y^{\varepsilon ,n_h}_s)+\int _s^t d_{{\mathscr {K}}}(\mu _\tau ^\varepsilon )\,\mathrm{d}\tau \\&=U(t,Y^{\varepsilon ,n_h}_t)-U(s,X)+\int _s^t d_{{\mathscr {K}}}(\mu _\tau ^\varepsilon )\,\mathrm{d}\tau \le \phi (t,Y^{\varepsilon ,n_h}_t)-\phi (s,X) +\int _s^t d_{{\mathscr {K}}}(\mu _\tau ^\varepsilon )\,\mathrm{d}\tau . \end{aligned}$$

Therefore, there exists a continuous increasing function $\varrho :[0,+\infty [\rightarrow [0,+\infty [$ with $\varrho (k)/k\rightarrow 0$ as $k\rightarrow 0^+$ such that we have

$$\begin{aligned} 0&\le \phi (t,Y^{\varepsilon ,n_h}_t)-\phi (s,X)+\int _s^t d_{{\mathscr {K}}}(\mu _\tau ^\varepsilon )\,\mathrm{d}\tau \\&\le \partial _t\phi (s,X)(t-s)+\langle D\phi (s,X),Y^{\varepsilon ,n_h}_t-X\rangle _{L^2_{{\mathbb {P}}}}+\int _s^t d_{{\mathscr {K}}}(\mu _\tau ^\varepsilon )\,\mathrm{d}\tau +\\&\quad +\varrho \left( |t-s|\left( 1+\left\| \dfrac{Y^{\varepsilon ,n_h}_t-Y^{\varepsilon ,n_h}_s}{t-s}\right\| _{L^2_{{\mathbb {P}}}}\right) +\dfrac{1}{n_h}\right) \\&\le \partial _t\phi (s,X)(t-s)+\langle D\phi (s,X),Y^{\varepsilon ,n_h}_t-Y^{\varepsilon ,n_h}_s\rangle _{L^2_{{\mathbb {P}}}}+\int _s^t d_{{\mathscr {K}}}(\mu _\tau ^\varepsilon )\,\mathrm{d}\tau +\dfrac{1}{n_h}\Vert D\phi (s,X)\Vert _{L^2_{{\mathbb {P}}}}\\&\quad +\varrho \left( |t-s|\left( 1+\left\| \dfrac{e_t-e_s}{t-s}\right\| _{L^2_{\varvec{\eta ^\varepsilon }}}\right) +\dfrac{1}{n_h}\right) . \end{aligned}$$

Dividing by $t-s>0$, and recalling the choice of $v_\varepsilon$, we have

$$\begin{aligned} 0\le&\partial _t\phi (s,X)+\langle D\phi (s,X),\frac{Y^{\varepsilon ,n_h}_t-Y^{\varepsilon ,n_h}_s}{t-s}\rangle _{L^2_{{\mathbb {P}}}}+\dfrac{1}{t-s}\int _s^t d_{{\mathscr {K}}}(\mu _\tau ^\varepsilon )\,\mathrm{d}\tau +\\&+\dfrac{1}{n_h}\cdot \dfrac{1}{t-s}\Vert D\phi (s,X)\Vert _{L^2_{\mathbb P}}+\dfrac{1}{t-s}\varrho \left( |t-s|\left( 1+\left\| \dfrac{e_t-e_s}{t-s}\right\| _{L^2_{\varvec{\eta ^\varepsilon }}}\right) +\dfrac{1}{n_h}\right) \\ \le&\partial _t\phi (s,X)+\langle D\phi (s,X),v_\varepsilon \circ X\rangle _{L^2_{{\mathbb {P}}}}+\\&+\Vert D\phi (s,X)\Vert _{L^2_{{\mathbb {P}}}}\left( \Vert v_\varepsilon \circ X-{\hat{v}}_\varepsilon \circ X\Vert _{L^2_{{\mathbb {P}}}}+\Vert {\hat{v}}_\varepsilon \circ X-{\hat{v}}_\varepsilon \circ Y^{\varepsilon ,n_h}_s\Vert _{L^2_{{\mathbb {P}}}}\right. +\\&+\left. \Vert {\hat{v}}_\varepsilon \circ Y^{\varepsilon ,n_h}_s-v_\varepsilon \circ Y^{\varepsilon ,n_h}_s\Vert _{L^2_{{\mathbb {P}}}}+\left\| \frac{Y^{\varepsilon ,n_h}_t-Y^{\varepsilon ,n_h}_s}{t-s}-v_\varepsilon \circ Y^{\varepsilon ,n_h}_s\right\| _{L^2_{{\mathbb {P}}}}\right) +\\&+\dfrac{1}{t-s}\int _s^t d_{{\mathscr {K}}}(\mu _\tau ^\varepsilon )\,\mathrm{d}\tau +\dfrac{1}{n_h}\cdot \dfrac{1}{t-s}\Vert D\phi (s,X)\Vert _{L^2_{{\mathbb {P}}}}+\\&+\dfrac{1}{t-s}\varrho \left( |t-s|\left( 1+\left\| \dfrac{e_t-e_s}{t-s}\right\| _{L^2_{\varvec{\eta ^\varepsilon }}}\right) +\dfrac{1}{n_h}\right) \\ \le&\partial _t\phi (s,X)+\mathop {{{\,\mathrm{inf}\,}}}\limits _{\begin{array}{c} v\in L^2_{X\sharp {\mathbb {P}}}({\mathbb {R}}^d)\\ v(\cdot )\in F(X\sharp {\mathbb {P}},\cdot ) \end{array}}\int _{\Omega }\langle D\phi (s,X)(\omega ),v\circ X(\omega )\rangle \,\mathrm{d}{\mathbb {P}}(\omega )+\dfrac{\varepsilon }{2}+\\&+\Vert D\phi (s,X)\Vert _{L^2_{{\mathbb {P}}}}\left( 2\varepsilon +\Vert {\hat{v}}_\varepsilon \circ X-{\hat{v}}_\varepsilon \circ Y^{\varepsilon ,n_h}_s\Vert _{L^2_{{\mathbb {P}}}}+\left\| \dfrac{e_t-e_s}{t-s}-v_\varepsilon \circ e_s\right\| _{L^2_{\varvec{\eta ^\varepsilon }}}\right) +\\&+\dfrac{1}{t-s}\int _s^t d_{{\mathscr {K}}}(\mu _\tau ^\varepsilon )\,\mathrm{d}\tau +\dfrac{1}{n_h}\cdot \dfrac{1}{t-s}\Vert D\phi (s,X)\Vert _{L^2_{{\mathbb {P}}}}+\dfrac{1}{t-s}\varrho \left( |t-s|(1+ \Vert \dfrac{e_t-e_s}{t-s}\Vert _{L^2_{\varvec{\eta ^\varepsilon }}})+\dfrac{1}{n_h}\right) . \end{aligned}$$

By letting $h\rightarrow +\infty$ and thanks to (6.7), we have

$$\begin{aligned} 0\le&\partial _t\phi (s,X)+\mathop {{{\,\mathrm{inf}\,}}}\limits _{\begin{array}{c} v\in L^2_{X\sharp {\mathbb {P}}}({\mathbb {R}}^d)\\ v(\cdot )\in F(X\sharp {\mathbb {P}},\cdot ) \end{array}}\int _{\Omega }\langle D\phi (s,X)(\omega ),v\circ X(\omega )\rangle \,\mathrm{d}{\mathbb {P}}(\omega )+\dfrac{\varepsilon }{2}+\\&+\Vert D\phi (s,X)\Vert _{L^2_{{\mathbb {P}}}}\left( 2\varepsilon +\left\| \dfrac{e_t-e_s}{t-s}-v_\varepsilon \circ e_s\right\| _{L^2_{\varvec{\eta ^\varepsilon }}}\right) +\\&+\dfrac{1}{t-s}\int _s^t d_{{\mathscr {K}}}(\mu _\tau ^\varepsilon )\,\mathrm{d}\tau +\dfrac{1}{t-s}\varrho \left( |t-s|\left( 1+\left\| \dfrac{e_t-e_s}{t-s}\right\| _{L^2_{\varvec{\eta ^\varepsilon }}}\right) \right) . \end{aligned}$$

By letting $t\rightarrow s^+$ and recalling the boundedness of $\left\| \dfrac{e_t-e_s}{t-s}\right\| _{L^2_{\varvec{\eta ^\varepsilon }}}$ coming from Proposition 3.4, we have

$$\begin{aligned} 0&\le \partial _t\phi (s,X)+\mathop {{{\,\mathrm{inf}\,}}}\limits _{\begin{array}{c} v\in L^2_{X\sharp {\mathbb {P}}}({\mathbb {R}}^d)\\ v(\cdot )\in F(X\sharp {\mathbb {P}},\cdot ) \end{array}}\int _{\Omega }\langle D\phi (s,X)(\omega ),v\circ X(\omega )\rangle \,\mathrm{d}{\mathbb {P}}(\omega ) +\dfrac{\varepsilon }{2} +2\varepsilon \Vert D\phi (s,X)\Vert _{L^2_{{\mathbb {P}}}}+d_{{\mathscr {K}}}(\mu ). \end{aligned}$$

Finally, letting $\varepsilon \rightarrow 0^+$ yields

$$\begin{aligned} 0\le&\partial _t\phi (s,X)+\mathop {{{\,\mathrm{inf}\,}}}\limits _{\begin{array}{c} v\in L^2_{X\sharp {\mathbb {P}}}({\mathbb {R}}^d)\\ v(\cdot )\in F(X\sharp {\mathbb {P}},\cdot ) \end{array}}\int _{\Omega }\langle D\phi (s,X)(\omega ),v\circ X(\omega )\rangle \,\mathrm{d}{\mathbb {P}}(\omega )+d_{{\mathscr {K}}}(\mu _s), \end{aligned}$$

i.e., in view of Definition 6.1, $-\partial _t\phi (s,X)+H_F^{\mathrm {viab}}(X,D\phi (s,X))\le 0$.

The proof of item (2) is omitted since it is a straightforward adaption of the previous argument just provided for item (1). We specify that, in this case, the proofs of the assertions regarding subsolutions and supersolutions are reversed, minimum has to be replaced by maximum and vice versa, the inequality signs are reversed and the signs of the terms involving $\rho$ and $\varepsilon$ need to be changed accordingly. $\square$

We finish the section with our main results: a viscosity characterization of viability (Theorem 6.6) and invariance (Theorem 6.7).

### Theorem 6.6

(Characterization of viability) Assume $\varvec{(F_1)-(F_2)}$ and let $L=\mathrm {Lip}(F)$ and ${\mathscr {H}}_F^{\mathrm {viab}}$ as in Definition 6.1. Consider a $W_2$-closed subset ${\mathscr {K}}\subseteq {\mathscr {P}}_2({\mathbb {R}}^d)$. The following are equivalent:

1. the function $z:[0,T]\times {\mathscr {P}}_2({\mathbb {R}}^d)\rightarrow {\mathbb {R}}$, defined by $z(t,\mu ):=d_{{\mathscr {K}}}(\mu )$, is a viscosity supersolution of
  6.8 $$\begin{aligned} (L+2) u(t,\mu ) +{\mathscr {H}}_F^{\mathrm {viab}}(\mu ,D_\mu u(t,\mu ))=0,\quad \text {in }[0,T]\times {\mathscr {P}}_2({\mathbb {R}}^d); \end{aligned}$$
2. there exists $T>0$ such that the function $w:[0,T]\times {\mathscr {P}}_2({\mathbb {R}}^d)\rightarrow {\mathbb {R}}$, defined by
  6.9 $$\begin{aligned} w(t,\mu ):=\dfrac{e^{-(L+1)(t-T)}-1}{L+1}d_{{\mathscr {K}}}(\mu ), \end{aligned}$$
  is a viscosity supersolution of
  6.10 $$\begin{aligned} -\partial _t u(t,\mu )+{\mathscr {H}}_F^{\mathrm {viab}}(\mu ,D_\mu u(t,\mu ))=0,\quad \text {in }[0,T]\times {\mathscr {P}}_2({\mathbb {R}}^d); \end{aligned}$$
3. ${\mathscr {K}}$ is *viable* for the dynamics *F*.

### Proof

For any $T>0$, consider the decreasing function $\alpha :[0,T]\rightarrow {\mathbb {R}}$ defined as

6.11 $$\begin{aligned} \alpha (t)=\dfrac{e^{-(L+1)(t-T)}-1}{L+1}. \end{aligned}$$

We denote by $W(t,X):=w(t,X\sharp {\mathbb {P}})$ the lift of $w(\cdot )$ according to Definition 5.1(1).

*Proof of* $(1\Rightarrow 2)$. Let $d_{{\mathscr {K}}}$ be a supersolution to (6.8) (cf. Remark 5.3). Fix $t\in [0,T)$, $\mu$ and $X \in L^2 _{ {\mathbb {P}}}(\Omega )$. Let $\Psi :[0,T]\times L^2_{{\mathbb {P}}}(\Omega )\rightarrow {\mathbb {R}}$ be a $C^1$ test function such that $W-\Psi$ has a local minimum at (*t*, *X*). We want to prove that

$$\begin{aligned}-\partial _t\Psi (t,X)+H_F^{\mathrm {viab}}(X,D\Psi (t,X))\ge 0.\end{aligned}$$

Since $s\mapsto \alpha (s) d_{{\mathscr {K}}}(Y\sharp {\mathbb {P}})=W(s,Y)$ is regular for any $Y\in L^2_{{\mathbb {P}}}(\Omega )$, then by the minimality we should have

$$\begin{aligned} \partial _s\Psi (t,X)=\partial _s W(t,X),\quad \text {i.e. }\partial _s\Psi (t,X)={\dot{\alpha }}(t)d_{{\mathscr {K}}}(X\sharp {\mathbb {P}}). \end{aligned}$$

Hence, for all $(s,Y) \in [0,T]\times L^2 _{ {\mathbb {P}}}(\Omega )$ in a small enough neighborhood $I_{t,X}$ of (*t*, *X*),

6.12 $$\begin{aligned} \begin{aligned}&\Psi (s,Y)=\alpha (s)\varphi (Y)+g(s,Y),\\ \text {with }&\varphi \in C^1(L^2_{{\mathbb {P}}}(\Omega )) \text { s.t. }\varphi (X)=d_{{\mathscr {K}}}(X\sharp {\mathbb {P}}),\\&g\in C^1([0,T]\times L^2_{{\mathbb {P}}}(\Omega )) \text { s.t. }\partial _t g(t,X)=0, \end{aligned} \end{aligned}$$

and $\varphi , g$ such that

6.13 $$\begin{aligned} W(s,Y)-\Psi (s,Y)\ge W(t,X)-\Psi (t,X), \end{aligned}$$

by local minimality of (*t*, *X*). By definition of *W* and (6.13), we get

$$\begin{aligned}\alpha (s)[d_{{\mathscr {K}}}(Y\sharp {\mathbb {P}})-\varphi (Y)]\ge g(s,Y)-g(t,X),\end{aligned}$$

for any $(s,Y)\in I_{t,X}$. In particular, by choosing $s=t$, we obtain

$$\begin{aligned} d_{{\mathscr {K}}}(Y\sharp {\mathbb {P}})\ge \varphi (Y)+\frac{1}{\alpha (t)}[g(t,Y)-g(t,X)], \end{aligned}$$

with equality holding when $Y=X$. Thus, denoting with $\Phi _t:L^2_{{\mathbb {P}}}(\Omega )\rightarrow {\mathbb {R}}$ the function given by

$$\begin{aligned}\Phi _t(Y):=\varphi (Y)+\frac{1}{\alpha (t)}[g(t,Y)-g(t,X)],\end{aligned}$$

we notice that $\Phi _t\in C^1(L^2_{{\mathbb {P}}}(\Omega ))$ and that the map $Y\mapsto d_{{\mathscr {K}}}(Y\sharp {\mathbb {P}})-\Phi _t(Y)$ attains a local minimum in *X*. Thus, recalling also Remark 5.3, we can employ $\Phi _t$ as a test function for $d_{{\mathscr {K}}}$ to get

6.14 $$\begin{aligned} (L+2) d_{{\mathscr {K}}}(X\sharp {\mathbb {P}})+H_F^{\mathrm {viab}}(X,D\Phi _t(X))\ge 0. \end{aligned}$$

Notice that by (6.12),

6.15 $$\begin{aligned} \begin{aligned} \partial _t\Psi (t,X)&={\dot{\alpha }}(t) d_{{\mathscr {K}}}(X\sharp {\mathbb {P}})=-[(L+1)\alpha (t)+1] d_{{\mathscr {K}}}(X\sharp {\mathbb {P}}),\\ D\Psi (t,X)&=\alpha (t)D\varphi (X)+Dg(t,X)=\alpha (t) D\Phi _t(X). \end{aligned} \end{aligned}$$

Recalling the definition of the lifted Hamiltonian $H_F^{\mathrm {viab}}$, by (6.14) we obtain

$$\begin{aligned} 0&\le (L+2) d_{{\mathscr {K}}}(X\sharp {\mathbb {P}})+H_F^{\mathrm {viab}}\left( X,\frac{1}{\alpha (t)}D\Psi (t,X)\right) \\&=(L+2) d_{{\mathscr {K}}}(X\sharp {\mathbb {P}})-d_{{\mathscr {K}}}(X\sharp {\mathbb {P}}) -\frac{1}{\alpha (t)} \mathop {{{\,\mathrm{inf}\,}}}\limits _{\begin{array}{c} v\in L^2_{{\mathbb {P}}}(\Omega )\\ v(\cdot )\in F(X\sharp {\mathbb {P}},X(\cdot )) \end{array}}\int _{\Omega }\langle v(\omega ),D\Psi (t,X)(\omega )\rangle \,\mathrm{d}{\mathbb {P}}(\omega ). \end{aligned}$$

Multiplying by $\alpha (t)$, we finally get

$$\begin{aligned}d_{{\mathscr {K}}}(X\sharp {\mathbb {P}})+H_F^{\mathrm {viab}}(X,D\Psi (t,X))\ge 0,\end{aligned}$$

thus

$$\begin{aligned}-\partial _t\Psi (t,X)+H_F^{\mathrm {viab}}(X,D\Psi (t,X))\ge 0,\end{aligned}$$

which concludes that *w* is a supersolution of (6.10).

*Proof of* $(2\Rightarrow 3)$. Let $T>0$ and assume that $w(t,\mu )=\alpha (t)d_{{\mathscr {K}}}(\mu )$ is a viscosity supersolution of (6.10). We recall that $H_F^{\mathrm {viab}}$, given in Definition 6.1, satisfies the assumptions of Theorem 5.4 as proved in Lemma 6.3. In particular, if we denote by $U(t,X):=V^{\mathrm {viab}}(t,X\sharp {\mathbb {P}})$ the lift of the value function of Definition 4.1, we have

$$\begin{aligned}W(T,X)=U(T,X)=0,\text { for every }X\in L^2_{{\mathbb {P}}}(\Omega ).\end{aligned}$$

Therefore, since both *w* and $V^{\mathrm {viab}}$ are uniformly continuous (see Proposition 4.6), by Theorem 5.4 and Proposition 6.5, we have $U(t,X)\le W(t,X)$ for all $(t,X)\in [0,T]\times L^2_{{\mathbb {P}}}(\Omega )$. Thus, for all $\mu \in {\mathscr {K}}$ and all $X\in L^2_{{\mathbb {P}}}(\Omega )$ with $X\sharp {\mathbb {P}}=\mu$ we obtain $V^{\mathrm {viab}}(t,\mu )=U(t,X)=W(t,X)=0$ for all $t\in [0,T]$. By Proposition 4.3, we conclude that there exists an admissible trajectory starting from $\mu$ and defined on [0, *T*], which is entirely contained in ${\mathscr {K}}$. So ${\mathscr {K}}$ is viable.

*Proof of* $(3\Rightarrow 1)$. Assume that ${\mathscr {K}}$ is viable. Set ${\hat{d}}_{{\mathscr {K}}}(Y):=d_{{\mathscr {K}}}(Y\sharp {\mathbb {P}})$ for all $Y\in L^2_{{\mathbb {P}}}(\Omega )$, i.e., ${\hat{d}}_{{\mathscr {K}}}$ is the lift of $d_{{\mathscr {K}}}$. Let $\phi \in C^1(L^2_{{\mathbb {P}}}(\Omega ))$ and $X\in L^2_{{\mathbb {P}}}(\Omega )$ be such that ${\hat{d}}_{{\mathscr {K}}}-\phi$ has a local minimum at *X*, and set $\mu =X\sharp {\mathbb {P}}\in {\mathscr {P}}_2({\mathbb {R}}^d)$.

For any $\varepsilon >0$ and $T>0$, there exist ${\bar{\mu }}^\varepsilon \in {\mathscr {K}}$, and $\varvec{{\bar{\mu }}}^\varepsilon \in {\mathcal {A}}_{[0,T]}({\bar{\mu }}^\varepsilon )$ satisfying $W_2(\mu ,{\bar{\mu }}^\varepsilon )\le d_{{\mathscr {K}}}(\mu )+\varepsilon$ and $\varvec{{\bar{\mu }}}^\varepsilon \subseteq {\mathscr {K}}$. By Grönwall’s inequality (Lemma 3.3), there exists $\varvec{\mu }^\varepsilon \in {\mathcal {A}}_{[0,T]}(\mu )$, $\varvec{\eta }^\varepsilon \in {\mathscr {P}}({\mathbb {R}}^d\times \Gamma _{[0,T]})$ such that $\mu ^\varepsilon _t=e_t\sharp \varvec{\eta }^\varepsilon$, and

$$\begin{aligned}d_{{\mathscr {K}}}(\mu ^\varepsilon _t)\le W_2(\mu _t^\varepsilon ,{\bar{\mu }}_t^\varepsilon )\le e^{Lt+te^{Lt}}\cdot W_2(\mu ,{\bar{\mu }}^\varepsilon )\le e^{Lt+te^{Lt}}\cdot (d_{{\mathscr {K}}}(\mu )+\varepsilon ),\end{aligned}$$

for all $$t\in [0,T]$$.

According to Corollary A.3 applied to $\varvec{\mu }^\varepsilon$, set

$$\begin{aligned}\widehat{\varpi }(t):=\dfrac{L}{t}\int _0^t\left[ W_2(\mu ^\varepsilon _\tau ,\mu ^\varepsilon _0)+\Vert e_\tau -e_0\Vert _{L^2_{\varvec{\eta }^\varepsilon }}\right] \,\mathrm{d}\tau ,\end{aligned}$$

there exists a family $\{Y^\varepsilon _t\}_{t\in [0,T]}\subseteq L^2_{{\mathbb {P}}}(\Omega )$ satisfying $Y^\varepsilon _t\sharp {\mathbb {P}}=\mu _t^\varepsilon$ for all $t\in [0,T]$ and

$$\begin{aligned} \langle p,\dfrac{Y^\varepsilon _t-Y^\varepsilon _0}{t}\rangle _{L^2_{{\mathbb {P}}}}\ge&\int _{\Omega }\mathop {{{\,\mathrm{inf}\,}}}\limits _{v\in F(X\sharp {\mathbb {P}},X(\omega ))} \langle p(\omega ),v\rangle \,\mathrm{d}{\mathbb {P}}(\omega )-(\widehat{\varpi }(t)+L\varepsilon )\Vert p\Vert _{L^2_{{\mathbb {P}}}}\\ =&-d_{{\mathscr {K}}}(X\sharp {\mathbb {P}})-H_F^{\mathrm {viab}}(X,p)-(\widehat{\varpi }(t)+L\varepsilon )\Vert p\Vert _{L^2_{{\mathbb {P}}}} \end{aligned}$$

for any $p\in L^2_{{\mathbb {P}}}(\Omega )$ (recall that $\mu =\mu _0=X\sharp {\mathbb {P}}=Y^\varepsilon _0\sharp {\mathbb {P}}$). According to the choice of *X*, we have

6.16 $$\begin{aligned} \dfrac{d_{{\mathscr {K}}}(\mu ^\varepsilon _t)-d_{{\mathscr {K}}}(\mu )}{t}=\dfrac{{\hat{d}}_{{\mathscr {K}}}(Y^\varepsilon _t)-{\hat{d}}_{{\mathscr {K}}}(X)}{t}\ge \dfrac{\phi (Y^\varepsilon _t)-\phi (X)}{t}. \end{aligned}$$

We estimate the first term as follows

$$\begin{aligned} \dfrac{d_{{\mathscr {K}}}(\mu ^\varepsilon _t)-d_{{\mathscr {K}}}(\mu )}{t}&\le \dfrac{W_2(\mu ^\varepsilon _t,{\bar{\mu }}^\varepsilon _t)-W_2(\mu ,{\bar{\mu }}^\varepsilon )}{t}+\dfrac{\varepsilon }{t}\le \dfrac{e^{Lt+te^{Lt}}-1}{t}\cdot W_2(\mu ,{\bar{\mu }}^\varepsilon )+\dfrac{\varepsilon }{t}\\&\le \dfrac{e^{Lt+te^{Lt}}-1}{t}\cdot (d_{{\mathscr {K}}}(X\sharp {\mathbb {P}})+\varepsilon )+\dfrac{\varepsilon }{t}. \end{aligned}$$

Concerning the right hand side of (6.16), we have that there exists a continuous increasing map $\varrho :[0,+\infty )\rightarrow [0,+\infty )$ with $\varrho (r)/r\rightarrow 0$ as $r\rightarrow 0^+$ such that

$$\begin{aligned} \begin{aligned} \dfrac{\phi (Y^\varepsilon _t)-\phi (X)}{t}&\ge \langle D\phi (X),\dfrac{Y^\varepsilon _t-X}{t}\rangle _{L^2_{{\mathbb {P}}}}-\dfrac{\varrho (\Vert Y^\varepsilon _t-X\Vert _{L^2_{{\mathbb {P}}}}+t)}{t}\\ {}&\ge \langle D\phi (X),\dfrac{Y^\varepsilon _t-Y^\varepsilon _0}{t}\rangle _{L^2_{{\mathbb {P}}}}-\Vert D\phi (X)\Vert _{L^2_{{\mathbb {P}}}}\cdot \dfrac{\Vert X-Y^\varepsilon _0\Vert _{L^2_{{\mathbb {P}}}}}{t}+\\ {}&\quad -\dfrac{1}{t}\varrho (\Vert Y^\varepsilon _t-Y^\varepsilon _0\Vert _{L^2_{{\mathbb {P}}}}+\Vert Y^\varepsilon _0-X\Vert _{L^2_{{\mathbb {P}}}}+t)\\ {}&\ge \langle D\phi (X),\dfrac{Y^\varepsilon _t-Y^\varepsilon _0}{t}\rangle _{L^2_{{\mathbb {P}}}}-\dfrac{\varepsilon }{t}\Vert D\phi (X)\Vert _{L^2_{{\mathbb {P}}}} -\dfrac{\varrho (\Vert e_t-e_0\Vert _{L^2_{\varvec{\eta }^\varepsilon }}+t+\varepsilon )}{t}\\ {}&\ge -d_{{\mathscr {K}}}(X\sharp {\mathbb {P}})-H_F^{\mathrm {viab}}(X,D\phi (X))-\left( \widehat{\varpi }(t)+L\varepsilon +\dfrac{\varepsilon }{t}\right) \Vert D\phi (X)\Vert _{L^2_{{\mathbb {P}}}}+\\ {}&\quad -\dfrac{\varrho \left( t\left( \Vert \frac{e_t-e_0}{t}\Vert _{L^2_{\varvec{\eta }^\varepsilon }}+1\right) +\varepsilon \right) }{t}, \end{aligned} \end{aligned}$$

where in the third inequality we employed the definition of $Y_t^\varepsilon$ provided in the proof of Corollary A.3, i.e., $Y_t^\varepsilon =e_t\circ {\mathscr {W}}_\varepsilon$ for any $t\in [0,T]$, for some ${\mathscr {W}}_\varepsilon :\Omega \rightarrow {\mathbb {R}}^d\times \Gamma _{[0,T]}$ s.t. ${\mathscr {W}}_\varepsilon \sharp {\mathbb {P}}=\varvec{\eta }^\varepsilon$. Recalling now the uniform boundedness in $\varepsilon$ of $\Vert \frac{e_t-e_0}{t}\Vert _{L^2_{\varvec{\eta }^\varepsilon }}$ coming from Proposition 3.4(3), by letting $\varepsilon \rightarrow 0^+$ and $t\rightarrow 0^+$, and by setting

$$\begin{aligned}\ell :=\liminf _{t\rightarrow 0^+}\liminf _{\varepsilon \rightarrow 0^+}\dfrac{\phi (Y^\varepsilon _t)-\phi (X)}{t},\end{aligned}$$

we have

$$\begin{aligned} -d_{{\mathscr {K}}}(X\sharp {\mathbb {P}})-H_F^{\mathrm {viab}}(X,D\phi (X))\le \ell \le&(L+1)\cdot d_{{\mathscr {K}}}(X\sharp {\mathbb {P}}). \end{aligned}$$

This leads to $(L+2)d_{{\mathscr {K}}}(X\sharp {\mathbb {P}})+H_F^{\mathrm {viab}}(X,D\phi (X))\ge 0$, i.e., $d_{{\mathscr {K}}}(\mu )$ is a supersolution of (6.8). $\square$

### Theorem 6.7

(Characterization of invariance) Assume $\varvec{(F_1)-(F_2)}$ and let $L=\mathrm {Lip}(F)$ and ${\mathscr {H}}_F^{\mathrm {inv}}$ as in Definition 6.2. Consider a $W_2$-closed subset ${\mathscr {K}}\subseteq {\mathscr {P}}_2({\mathbb {R}}^d)$. The following is equivalent:

1. the function $z:[0,T]\times {\mathscr {P}}_2({\mathbb {R}}^d)\rightarrow {\mathbb {R}}$, defined by $z(t,\mu ):=d_{{\mathscr {K}}}(\mu )$, is a viscosity supersolution of
  6.17 $$\begin{aligned} (L+2) u(t,\mu ) +{\mathscr {H}}_F^{\mathrm {inv}}(\mu ,D_\mu u(t,\mu ))=0\quad \text {in }[0,T]\times {\mathscr {P}}_2({\mathbb {R}}^d); \end{aligned}$$
2. there exists $T>0$ such that the function $w:[0,T]\times {\mathscr {P}}_2({\mathbb {R}}^d)\rightarrow {\mathbb {R}}$, defined by (6.9), is a viscosity supersolution of
  6.18 $$\begin{aligned} -\partial _t u(t,\mu )+{\mathscr {H}}_F^{\mathrm {inv}}(\mu ,D_\mu u(t,\mu ))=0\quad \text {in }[0,T]\times {\mathscr {P}}_2({\mathbb {R}}^d); \end{aligned}$$
3. ${\mathscr {K}}$ is *invariant* for the dynamics *F*.

### Proof

For any $T>0$, consider the decreasing function $\alpha :[0,T]\rightarrow {\mathbb {R}}$ defined as in (6.11).

We denote by $W(t,X):=w(t,X\sharp {\mathbb {P}})$ the lift of $w(\cdot )$ defined in (6.9) according to Definition 5.1(1).

*Proof of* $(1\Rightarrow 2)$. This part of the proof is the same as the one developed in Theorem 6.6 with $H_F^{\mathrm {inv}}$ in place of $H_F^{\mathrm {viab}}$.

*Proof of* $(2\Rightarrow 3)$. Same as in Theorem 6.6, with $V^{\mathrm {viab}}$ replaced by $V^{\mathrm {inv}}$.

*Proof of* $(3\Rightarrow 1)$. Assume that ${\mathscr {K}}$ is invariant. Set $\hat{d}_{{\mathscr {K}}}(Y)=d_{{\mathscr {K}}}(Y\sharp {\mathbb {P}})$ for all $Y\in L^2_{{\mathbb {P}}}(\Omega )$, i.e., $\hat{d}_{{\mathscr {K}}}$ is the lift of $d_{{\mathscr {K}}}$. Let $\phi \in C^1(L^2_{{\mathbb {P}}}(\Omega ))$ and $X\in L^2_{{\mathbb {P}}}(\Omega )$ be such that $\hat{d}_{{\mathscr {K}}}-\phi$ has a local minimum at *X*, and set $\mu =X\sharp {\mathbb {P}}\in {\mathscr {P}}_2({\mathbb {R}}^d)$.

Fix $\varepsilon >0$, and let $v_\varepsilon \in L^2_{\mu }({\mathbb {R}}^d)$ be such that $v_\varepsilon (x)\in F(\mu ,x)$ for $\mu$-a.e. $x\in {\mathbb {R}}^d$ and

$$\begin{aligned}&\int _{\Omega } \langle D\phi (X)(\omega ),v_\varepsilon \circ X(\omega )\rangle \,\mathrm{d}{\mathbb {P}}(\omega ) \ge \sup _{\begin{array}{c} v\in L^2_{X\sharp {\mathbb {P}}}({\mathbb {R}}^d)\\ v(\cdot )\in F(X\sharp {\mathbb {P}},\cdot ) \end{array}}\int _{\Omega }\langle D\phi (X)(\omega ),v\circ X(\omega )\rangle \,\mathrm{d}{\mathbb {P}}(\omega )-\dfrac{\varepsilon }{2}.\end{aligned}$$

By Remark 6.4, we can suppose that $v_\varepsilon \in C^0$, and by Lemma A.4 there exists an admissible trajectory $\varvec{\mu }^\varepsilon =\{\mu ^\varepsilon _t\}_{t\in [0,T]}$ defined on [0, *T*] with $\mu ^\varepsilon _s=\mu$, and $\varvec{\eta }^\varepsilon \in {\mathscr {P}}({\mathbb {R}}^d\times \Gamma _{[0,T]})$ such that $e_t\sharp \varvec{\eta }^\varepsilon =\mu ^\varepsilon _t$ for all $t\in [0,T]$ and

$$\begin{aligned}\lim _{t\rightarrow 0^+}\left\| \dfrac{e_t-e_0}{t}-v_\varepsilon \circ e_0\right\| _{L^2_{\varvec{\eta }^\varepsilon }}=0.\end{aligned}$$

By density, we can find ${\hat{v}}_\varepsilon \in C^0_b({\mathbb {R}}^d)$ such that $\Vert v_\varepsilon -{\hat{v}}_\varepsilon \Vert _{L^2_{\mu }}\le \varepsilon$.

Denote by ${\mathscr {V}}_\varepsilon :\Omega \rightarrow {\mathbb {R}}^d\times \Gamma _{[0,T]}$ a Borel map satisfying $\varvec{\eta }^\varepsilon ={\mathscr {V}}_\varepsilon \sharp {\mathbb {P}}$. Recalling Lemma A.2, since for all $\varepsilon >0$ we have $\mu =\mu ^\varepsilon _0=e_0\sharp \varvec{\eta }^\varepsilon =(e_0\circ {\mathscr {V}}_\varepsilon )\sharp {\mathbb {P}}=X\sharp {\mathbb {P}}$, we can find a sequence of measure-preserving Borel maps $\{r^\varepsilon _{n}(\cdot )\}_{n\in {\mathbb {N}}}$ such that

$$\begin{aligned}{\mathbb {P}}\left( \left\{ \omega \in \Omega :\,|X(\omega )-e_0\circ {\mathscr {V}}_\varepsilon \circ r^\varepsilon _{n}(\omega )|\le \dfrac{1}{n}\right\} \right) =1,\end{aligned}$$

and we set $Y^{\varepsilon ,n}_t=e_t\circ {\mathscr {V}}_\varepsilon \circ r^\varepsilon _{n}$ for all $t\in [0,T]$. In particular, $Y^{\varepsilon ,n}_t\sharp {\mathbb {P}}=\mu ^\varepsilon _t$ for all $t\in [0,T]$. We then have

$$\begin{aligned}\lim _{t\rightarrow 0^+}\left\| \dfrac{Y^{\varepsilon ,n}_t-Y^{\varepsilon ,n}_0}{t}-v_\varepsilon \circ Y^{\varepsilon ,n}_0\right\| _{L^2_{{\mathbb {P}}}} =\lim _{t\rightarrow 0^+}\left\| \dfrac{e_t-e_0}{t}-v_\varepsilon \circ e_0\right\| _{L^2_{\varvec{\eta }^\varepsilon }}=0.\end{aligned}$$

Recalling the choice of ${\hat{v}}_\varepsilon$, we have also

$$\begin{aligned}\Vert v_\varepsilon \circ X-{\hat{v}}_\varepsilon \circ X\Vert _{L^2_{{\mathbb {P}}}}=\Vert v_\varepsilon \circ Y^{\varepsilon ,n}_0-{\hat{v}}_\varepsilon \circ Y^{\varepsilon ,n}_0\Vert _{L^2_{{\mathbb {P}}}}=\Vert v_\varepsilon -{\hat{v}}_\varepsilon \Vert _{L^2_\mu }\le \varepsilon .\end{aligned}$$

Since, by Lemma A.2, $\Vert Y^{\varepsilon ,n}_0-X\Vert _{L^2_{{\mathbb {P}}}}\le \frac{1}{n}$, we can find a subsequence $\{Y^{\varepsilon ,n_h}_0\}_{h\in {\mathbb {N}}}$ such that for ${\mathbb {P}}$-a.e. $\omega \in \Omega$ it holds $\lim _{h\rightarrow +\infty }Y^{\varepsilon ,n_h}_0(\omega )=X(\omega )$. Therefore,

6.19 $$\begin{aligned} \lim _{h\rightarrow +\infty }\int |{\hat{v}}_\varepsilon \circ Y^{\varepsilon ,n_h}_0(\omega )-{\hat{v}}_\varepsilon \circ X(\omega )|^2\,\mathrm{d}{\mathbb {P}}(\omega )=0, \end{aligned}$$

where we used the Dominated Convergence Theorem to pass to the limit under the integral sign, exploiting the global boundedness of ${\hat{v}}_\varepsilon$.

Now, let ${\bar{\mu }}^{n_h}\in {\mathscr {K}}$ such that $W_2(\mu ,{\bar{\mu }}^{n_h})\le d_{{\mathscr {K}}}(\mu )+\dfrac{1}{n_h}$. By Grönwall’s inequality (Lemma 3.3), given $\varvec{\mu }^\varepsilon$ as before there exist $\varvec{{\bar{\mu }}}^{\varepsilon ,n_h}\in {\mathcal {A}}_{[0,T]}({\bar{\mu }}^{n_h})$ such that

$$\begin{aligned} d_{{\mathscr {K}}}(\mu ^\varepsilon _t)\le W_2(\mu _t^\varepsilon ,{\bar{\mu }}_t^{\varepsilon ,n_h})\le e^{Lt+te^{Lt}}\cdot W_2(\mu ,{\bar{\mu }}^{n_h})\le e^{Lt+te^{Lt}}\cdot \left( d_{{\mathscr {K}}}(\mu )+\dfrac{1}{n_h}\right) , \end{aligned}$$

for all $t\in [0,T]$, where we used the fact that $\varvec{{\bar{\mu }}}^{\varepsilon ,n_h}\subseteq {\mathscr {K}}$ by invariance of the set ${\mathscr {K}}$ and since ${\bar{\mu }}^{\varepsilon ,n_h}_0={\bar{\mu }}^{n_h}\in {\mathscr {K}}$. According to the choice of *X*, we have

6.20 $$\begin{aligned} \dfrac{d_{{\mathscr {K}}}(\mu ^\varepsilon _t)-d_{{\mathscr {K}}}(\mu )}{t}=\dfrac{\hat{d}_{{\mathscr {K}}}(Y^{\varepsilon ,n_h}_t)-\hat{d}_{{\mathscr {K}}}(X)}{t}\ge \dfrac{\phi (Y^{\varepsilon ,n_h}_t)-\phi (X)}{t}. \end{aligned}$$

We estimate the first term as follows

6.21 $$\begin{aligned}&\dfrac{d_{{\mathscr {K}}}(\mu ^\varepsilon _t)-d_{{\mathscr {K}}}(\mu )}{t} \le \dfrac{W_2(\mu ^\varepsilon _t,{\bar{\mu }}^{\varepsilon ,n_h}_t)- W_2(\mu ,{\bar{\mu }}^{n_h})}{t}+\dfrac{1}{n_h}\cdot \dfrac{1}{t}\nonumber \\&\quad \le \dfrac{e^{Lt+te^{Lt}}-1}{t}\cdot W_2(\mu ,{\bar{\mu }}^{n_h})+\dfrac{1}{n_h}\cdot \dfrac{1}{t}\le \dfrac{e^{Lt+te^{Lt}}-1}{t}\cdot \left( d_{{\mathscr {K}}}(X\sharp \mu )+\dfrac{1}{n_h}\right) +\dfrac{1}{n_h}\cdot \dfrac{1}{t}.\nonumber \\ \end{aligned}$$

Concerning the right hand side of (6.20), we have that there exists a continuous increasing map $\varrho :[0,+\infty )\rightarrow [0,+\infty )$ with $\varrho (r)/r\rightarrow 0$ as $r\rightarrow 0^+$ such that

$$\begin{aligned} \dfrac{\phi (Y^{\varepsilon ,n_h}_t)-\phi (X)}{t} \ge&\langle D\phi (X),\dfrac{Y^{\varepsilon ,n_h}_t-X}{t}\rangle _{L^2_{{\mathbb {P}}}}-\dfrac{\varrho (\Vert Y^{\varepsilon ,n_h}_t-X\Vert _{L^2_{{\mathbb {P}}}}+t)}{t}\\ \ge&\langle D\phi (X),\dfrac{Y^{\varepsilon ,n_h}_t-Y^{\varepsilon ,n_h}_0}{t}\rangle _{L^2_{{\mathbb {P}}}}-\Vert D\phi (X)\Vert _{L^2_{{\mathbb {P}}}}\cdot \dfrac{\Vert X-Y^{\varepsilon ,n_h}_0\Vert _{L^2_{{\mathbb {P}}}}}{t}+\\&-\dfrac{1}{t}\varrho (\Vert Y^{\varepsilon ,n_h}_t-Y^{\varepsilon ,n_h}_0\Vert _{L^2_{{\mathbb {P}}}}+\Vert Y^{\varepsilon ,n_h}_0-X\Vert _{L^2_{{\mathbb {P}}}}+t)\\ \ge&\langle D\phi (X),\dfrac{Y^{\varepsilon ,n_h}_t-Y^{\varepsilon ,n_h}_0}{t}\rangle _{L^2_{{\mathbb {P}}}}-\dfrac{1}{n_h}\cdot \dfrac{1}{t}\Vert D\phi (X)\Vert _{L^2_{{\mathbb {P}}}}+\\&\qquad -\dfrac{\varrho \left( \Vert e_t-e_0\Vert _{L^2_{\varvec{\eta }^\varepsilon }}+t+\dfrac{1}{n_h}\right) }{t}. \end{aligned}$$

Recalling the choice of $v_\varepsilon$, we have

$$\begin{aligned}&\dfrac{\phi (Y^{\varepsilon ,n_h}_t)-\phi (X)}{t} \ge \langle D\phi (X),v_\varepsilon \circ X\rangle _{L^2_{{\mathbb {P}}}}+\\&\qquad -\Vert D\phi (X)\Vert _{L^2_{{\mathbb {P}}}}\left( \Vert v_\varepsilon \circ X-{\hat{v}}_\varepsilon \circ X\Vert _{L^2_{{\mathbb {P}}}}+\Vert {\hat{v}}_\varepsilon \circ X-{\hat{v}}_\varepsilon \circ Y^{\varepsilon ,n_h}_0\Vert _{L^2_{{\mathbb {P}}}}\right. +\\&\qquad +\left. \Vert {\hat{v}}_\varepsilon \circ Y^{\varepsilon ,n_h}_0-v_\varepsilon \circ Y^{\varepsilon ,n_h}_0\Vert _{L^2_{{\mathbb {P}}}}+\left\| \frac{Y^{\varepsilon ,n_h}_t-Y^{\varepsilon ,n_h}_0}{t}-v_\varepsilon \circ Y^{\varepsilon ,n_h}_0\right\| _{L^2_{{\mathbb {P}}}}\right) +\\&\qquad -\dfrac{1}{n_h}\cdot \dfrac{1}{t}\Vert D\phi (X)\Vert _{L^2_{{\mathbb {P}}}}-\dfrac{1}{t}\varrho \left( t\left( 1+\left\| \dfrac{e_t-e_0}{t}\right\| _{L^2_{\varvec{\eta }^\varepsilon }}\right) +\dfrac{1}{n_h}\right) \\&\quad \ge \sup _{\begin{array}{c} v\in L^2_{X\sharp {\mathbb {P}}}({\mathbb {R}}^d)\\ v(\cdot )\in F(X\sharp {\mathbb {P}},\cdot ) \end{array}}\int _{\Omega }\langle D\phi (X)(\omega ),v\circ X(\omega )\rangle \,\mathrm{d}{\mathbb {P}}(\omega )-\dfrac{\varepsilon }{2}+\\&\qquad -\Vert D\phi (X)\Vert _{L^2_{{\mathbb {P}}}}\left( 2\varepsilon +\Vert {\hat{v}}_\varepsilon \circ X-{\hat{v}}_\varepsilon \circ Y^{\varepsilon ,n_h}_0\Vert _{L^2_{{\mathbb {P}}}}+\left\| \dfrac{e_t-e_0}{t}-v_\varepsilon \circ e_0\right\| _{L^2_{\varvec{\eta }^\varepsilon }}\right) +\\&\qquad -\dfrac{1}{n_h}\cdot \dfrac{1}{t}\Vert D\phi (X)\Vert _{L^2_{{\mathbb {P}}}}-\dfrac{1}{t}\varrho \left( t\left( 1+\left\| \dfrac{e_t-e_0}{t}\right\| _{L^2_{\varvec{\eta }^\varepsilon }}\right) +\dfrac{1}{n_h}\right) . \end{aligned}$$

Recalling now the uniform boundedness in $\varepsilon$ of $\Vert \frac{e_t-e_0}{t}\Vert _{L^2_{\varvec{\eta }^\varepsilon }}$ coming from Proposition 3.4(3), by letting $h\rightarrow +\infty$, $t\rightarrow 0^+$ and $\varepsilon \rightarrow 0^+$, and by setting

$$\begin{aligned}\ell :=\liminf _{\varepsilon \rightarrow 0^+}\liminf _{t\rightarrow 0^+}\liminf _{h\rightarrow +\infty }\dfrac{\phi (Y^{\varepsilon ,n_h}_t)-\phi (X)}{t},\end{aligned}$$

we have, thanks also to (6.19),

6.22 $$\begin{aligned} \ell \ge \sup _{\begin{array}{c} v\in L^2_{X\sharp {\mathbb {P}}}({\mathbb {R}}^d)\\ v(\cdot )\in F(X\sharp {\mathbb {P}},\cdot ) \end{array}}\int _{\Omega }\langle D\phi (X)(\omega ),v\circ X(\omega )\rangle \,\mathrm{d}{\mathbb {P}}(\omega ) = -d_{{\mathscr {K}}}(X\sharp {\mathbb {P}})-H_F^{\mathrm {inv}}(X,D\phi (X)).\nonumber \\ \end{aligned}$$

Thus, by passing to the limit also in (6.21) and combining that estimate with (6.22), we get

$$\begin{aligned} - d_{{\mathscr {K}}}(X\sharp {\mathbb {P}})-H_F^{\mathrm {inv}}(X,D\phi (X))\le \ell \le (L+1)\cdot d_{{\mathscr {K}}}(X\sharp {\mathbb {P}}). \end{aligned}$$

This leads to $(L+2)d_{{\mathscr {K}}}(X\sharp {\mathbb {P}})+H_F^{\mathrm {inv}}(X,D\phi (X))\ge 0$, i.e., $d_{{\mathscr {K}}}(\mu )$ is a supersolution of (6.17) (cf. Remark 5.3). $\square$

## An example

Given $\mu \in {\mathscr {P}}_2({\mathbb {R}}^d)$, $x\in {\mathbb {R}}^d$, $u\in {\mathbb {R}}$, let $U=[1/2,3/2]$, $U'=[-3/2,3/2]$ and define the functions $f,g:{\mathscr {P}}_2({\mathbb {R}}^d)\times {\mathbb {R}}^d\times {\mathbb {R}}\rightarrow {\mathbb {R}}^d$ as

$$\begin{aligned} f(\mu ,x,u):=u\arctan (1-\mathrm {m}^{1/2}_2(\mu ))e^{-|x|^2}x,&g(\mu ,x,u):=&\pi u x. \end{aligned}$$

Define the set-valued maps $F,G:{\mathscr {P}}_2({\mathbb {R}}^d)\times {\mathbb {R}}^d\rightrightarrows {\mathbb {R}}^d$ as

$$\begin{aligned} F(\mu ,x):=\left\{ f(\mu ,x,u):\,u\in U\right\} ,&G(\mu ,x):=\left\{ g(\mu ,x,u):\,u\in U'\right\} , \end{aligned}$$

and the closed set

$$\begin{aligned}{\mathscr {K}}:=\{\mu \in {\mathscr {P}}_2({\mathbb {R}}^d):\, \mathrm {m}_2(\mu )\le 1\}=\{X\sharp {\mathbb {P}}:\, \Vert X\Vert _{L^2_{{\mathbb {P}}}(\Omega )}\le 1\}.\end{aligned}$$

Notice that *F*, *G* satisfy the assumptions $(\varvec{F_1})-(\varvec{F_2})$ and $G(\mu ,x)\supseteq F(\mu ,x)$. In particular,

$$\begin{aligned}F(X\sharp {\mathbb {P}},X(\omega ))=\left\{ \lambda \arctan (1-\Vert X\Vert _{L^2_{{\mathbb {P}}}})e^{-|X(\omega )|^2}X(\omega ):\,\lambda \in [1/2,3/2]\right\} .\end{aligned}$$

We have

$$\begin{aligned}d_{{\mathscr {K}}}(\mu )={\left\{ \begin{array}{ll}\mathrm {m}^{1/2}_2(\mu )-1,&{} \text { if }\mu \notin {\mathscr {K}},\\ 0,&{}\text { if }\mu \in {\mathscr {K}}.\end{array}\right. }\end{aligned}$$

Indeed, to prove that $d_{{\mathscr {K}}}(\mu )\le \mathrm {m}^{1/2}_2(\mu )-1$ for all $\mu \notin {\mathscr {K}}$, take a $W_2$-geodesic $\{\xi _t\}_{t\in [0,\mathrm {m}^{1/2}_2(\mu )]}$ with constant speed joining $\delta _0$ to $\mu \notin {\mathscr {K}}$. We have $\mathrm {m}^{1/2}_2(\xi _1)=W_2(\delta _0,\xi _1)=1$, and $W_2(\mu ,\delta _0)=W_2(\mu ,\xi _1)+1$. So $\xi _1\in {\mathscr {K}}$ and $d_{{\mathscr {K}}}(\mu )\le \mathrm {m}^{1/2}_2(\mu )-1$. Conversely, fix $\varepsilon >0$ and let $\mu _\varepsilon \in {\mathscr {K}}$ be such that $d_{{\mathscr {K}}}(\mu )\ge W_2(\mu ,\mu _\varepsilon )-\varepsilon$. Then, recalling that $W_2(\mu _\varepsilon ,\delta _0)\le 1$, we have

$$\begin{aligned}d_{{\mathscr {K}}}(\mu )+1\ge W_2(\mu ,\mu _\varepsilon )+W_2(\mu _\varepsilon ,\delta _0)-\varepsilon \ge W_2(\mu ,\delta _0)-\varepsilon =\mathrm {m}^{1/2}_2(\mu )-\varepsilon .\end{aligned}$$

By letting $\varepsilon \rightarrow 0^+$, we have the desired inequality.

The lift of $d_{{\mathscr {K}}}(\cdot )$ is the convex function ${\hat{U}}:L^2_{{\mathbb {P}}}(\Omega )\rightarrow {\mathbb {R}}$ defined as

$$\begin{aligned}{\hat{U}}(X)={\left\{ \begin{array}{ll}\Vert X\Vert _{L^2_{{\mathbb {P}}}}-1,&{}\text { if }\Vert X\Vert _{L^2_{{\mathbb {P}}}}\ge 1,\\ 0,&{} \text { otherwise}.\end{array}\right. }\end{aligned}$$

The function ${\hat{U}}(\cdot )$ is $C^1$ in the open set $D:=\{X\in L^2_{{\mathbb {P}}}:\, \Vert X\Vert _{L^2_{{\mathbb {P}}}}\ne 1\}$. Thus, if $\psi \in C^1(L^2_{{\mathbb {P}}}(\Omega ))$ is such that ${\hat{U}}-\psi$ attains a local minimum at $X\in D$ then

$$\begin{aligned}D\psi (X)=D{\hat{U}}(X)={\left\{ \begin{array}{ll}0,&{}\text { if }\Vert X\Vert _{L^2_{{\mathbb {P}}}}<1,\\ \dfrac{X}{\Vert X\Vert _{L^2_{{\mathbb {P}}}}},&{}\text { if }\Vert X\Vert _{L^2_{{\mathbb {P}}}}>1.\end{array}\right. }\end{aligned}$$

Let $\psi \in C^1(L^2_{{\mathbb {P}}})$ such that ${\hat{U}}-\psi$ attains a local minimum at $X\in L^2_{{\mathbb {P}}}$ with $\Vert X\Vert _{L^2_{{\mathbb {P}}}}=1$. By Propositions 1.2 and 1.5 in [17], we have that

$$\begin{aligned}D\psi (X)\in \partial {\hat{U}}(X):=\left\{ \xi \in L^2_{{\mathbb {P}}}(\Omega ):\, {\hat{U}}(Y)-{\hat{U}}(X)\ge \langle \xi ,Y-X\rangle _{L^2_{{\mathbb {P}}}},\,\forall \,Y\in L^2_{{\mathbb {P}}}\right\} .\end{aligned}$$

Conversely, given $\xi \in \partial {\hat{U}}(X)$, set $\psi (Y)={\hat{U}}(X)+\langle \xi ,Y-X\rangle _{L^2_{{\mathbb {P}}}}$. Then, $\psi \in C^1$, ${\hat{U}}-\psi$ has a minimum at *X*, and $D\psi (X)=\xi$.

We want to prove that if $\Vert X\Vert _{L^2_{{\mathbb {P}}}}=1$, then $\partial {\hat{U}}(X)=\{\lambda X:\, \lambda \in [0,1]\}$.

We prove $\supseteq$. Given $X,Y\in L^2_{{\mathbb {P}}}$ with $\Vert X\Vert _{L^2_{{\mathbb {P}}}}=1$, and $\lambda \in [0,1]$, it holds

$$\begin{aligned}\langle \lambda X,Y-X\rangle _{L^2_{{\mathbb {P}}}}\le \lambda (\Vert Y\Vert _{L^2_{{\mathbb {P}}}}-1)\le {\left\{ \begin{array}{ll} \Vert Y\Vert _{L^2_{{\mathbb {P}}}}-1={\hat{U}}(Y)-{\hat{U}}(X),&{}\text { if }\Vert Y\Vert _{L^2_{{\mathbb {P}}}}\ge 1,\\ 0={\hat{U}}(Y)-{\hat{U}}(X),&{}\text { if }\Vert Y\Vert _{L^2_{{\mathbb {P}}}}< 1. \end{array}\right. } \end{aligned}$$

Thus, in any case $\langle \lambda X,Y-X\rangle _{L^2_{{\mathbb {P}}}}\le {\hat{U}}(Y)-{\hat{U}}(X)$, proving $\supseteq$.

Conversely, we prove $\subseteq$. Let $X\in L^2_{{\mathbb {P}}}(\Omega )$, $\Vert X\Vert _{L^2_{{\mathbb {P}}}}=1$, so ${\hat{U}}(X)=0$. Assume that $\xi =\lambda X+\hat{\lambda } Z\in \partial {\hat{U}}(X)$, with $\Vert Z\Vert _{L^2_{{\mathbb {P}}}}=1$, $\langle Z,X\rangle _{L^2_{{\mathbb {P}}}}=0$ and $\lambda ,\hat{\lambda }\in {\mathbb {R}}$. We want to prove that $\lambda \in [0,1]$ and $\hat{\lambda }=0$. Indeed, for all $Y\in L^2_{{\mathbb {P}}}(\Omega )$ it holds

$$\begin{aligned} {\hat{U}}(Y)-{\hat{U}}(X)\!\ge \! \langle \xi ,Y-X\rangle _{L^2_{{\mathbb {P}}}}\!=\!\langle \lambda X\!+\!\hat{\lambda } Z,Y-X\rangle _{L^2_{{\mathbb {P}}}} \!=\!\hat{\lambda }\langle Y,Z\rangle +\lambda (\langle Y,X\rangle _{L^2_{{\mathbb {P}}}}-1). \end{aligned}$$

By taking $Y=aX+bZ$, we have ${\hat{U}}(Y)=\max \{0,\sqrt{|a|^2+|b|^2}-1\}$, and so

$$\begin{aligned} \max \{0,\sqrt{|a|^2+|b|^2}-1\}\ge b\hat{\lambda }+\lambda (a-1). \end{aligned}$$

- Choosing $(a,b)=(2,0)$ leads to $\lambda \le 1$. Choosing $(a,b)=(1/2,0)$ leads to $\lambda \ge 0$. Therefore, $0\le \lambda \le 1$.
- Choose $a=1$. Then for all $b>0$, we have $\dfrac{\sqrt{1+b^2}-1}{b}\ge \hat{\lambda }$, and by passing to the limit as $b\rightarrow 0^+$ we have $0\ge \hat{\lambda }$. For all $b<0$, we have $\dfrac{\sqrt{1+b^2}-1}{b}\le \hat{\lambda }$, and by passing to the limit as $b\rightarrow 0^-$ we have $0\le \hat{\lambda }$. Therefore, $\hat{\lambda }=0$.

We prove now that ${\mathscr {K}}$ is invariant for the dynamics *F*. Thanks to Theorem 6.7, we have to prove that for every $\psi \in C^1(L^2_{{\mathbb {P}}})$ such that ${\hat{U}}-\psi$ attains a local minimum at $X\in L^2_{{\mathbb {P}}}$ it holds

$$\begin{aligned}(L+2)d_{{\mathscr {K}}}(X\sharp {\mathbb {P}})+H^{\mathrm {inv}}_F(X,D\psi (X))\ge 0.\end{aligned}$$

We distinguish two cases

- when $\Vert X\Vert _{L^2_{{\mathbb {P}}}}<1$, we have $d_{{\mathscr {K}}}(X\sharp {\mathbb {P}})=0$ and $D\psi (X)=0$, which implies $H^{\mathrm {inv}}_F(X,D\psi (X))=0$, so the equation is trivially satisfied.
- when $\Vert X\Vert _{L^2_{{\mathbb {P}}}}\ge 1$, we have $d_{{\mathscr {K}}}(X\sharp {\mathbb {P}})=\Vert X\Vert _{L^2_{{\mathbb {P}}}}-1$ and $D\psi (X)=\lambda \dfrac{X}{\Vert X\Vert _{L^2_{{\mathbb {P}}}}}$, with $\lambda =1$ if $\Vert X\Vert _{L^2_{{\mathbb {P}}}}> 1$, and $\lambda \in [0,1]$ otherwise, which implies
  $$\begin{aligned} H^{\mathrm {inv}}_F(X,D\psi (X))=&1-\Vert X\Vert _{L^2_{{\mathbb {P}}}}-\dfrac{1}{2}\lambda \int _{\Omega }\arctan (1-\Vert X\Vert _{L^2_{{\mathbb {P}}}})e^{-|X(\omega )|^2}|X(\omega )|^2\,\mathrm{d}{\mathbb {P}}(\omega )\\ \ge&1-\Vert X\Vert _{L^2_{{\mathbb {P}}}}, \end{aligned}$$
  So, also in this case, we have
  $$\begin{aligned}(L+2)d_{{\mathscr {K}}}(X\sharp {\mathbb {P}})+H^{\mathrm {inv}}_F(X,D\psi (X))\ge (L+2)(\Vert X\Vert _{L^2_{{\mathbb {P}}}}-1)+1-\Vert X\Vert _{L^2_{{\mathbb {P}}}}\ge 0,\end{aligned}$$

from which we get the invariance, and thus the viability, of the set ${\mathscr {K}}$ for the dynamics *F*. Since all the admissible trajectories for *F* are also admissible for *G*, we have that ${\mathscr {K}}$ is viable for *G*. We prove now that ${\mathscr {K}}$ is not invariant for *G*. Indeed, take $X\in L^2_{{\mathbb {P}}}(\Omega )$ with $\Vert X\Vert _{L^2_{{\mathbb {P}}}}=1$. Then, we can consider $\psi \in C^1(L^2_{{\mathbb {P}}}(\Omega ))$ s.t. $\psi (Y)=\Vert Y\Vert _{L^2_{{\mathbb {P}}}}$ in a neighborhood *V* of *X*. Given $Y\in V$, we have ${\hat{U}}(Y)-\psi (Y)=-1$ if $\Vert Y\Vert _{L^2_{{\mathbb {P}}}}\ge 1$ and ${\hat{U}}(Y)-\psi (Y)=-\Vert Y\Vert _{L^2_{{\mathbb {P}}}}\ge -1$ if $\Vert Y\Vert _{L^2_{{\mathbb {P}}}}<1$. In particular, ${\hat{U}}(X)-\psi (X)=-1$, so ${\hat{U}}-\psi$ attains in *V* a minimum at *X*, and $D\psi (X)=X$. Set

$$\begin{aligned}H^{\mathrm {inv}}_G(Y,D\psi (Y))=-d_{{\mathscr {K}}}(Y\sharp {\mathbb {P}})-\sup _{\begin{array}{c} v\in L^2_{{\mathbb {P}}}(\Omega )\\ v(\cdot )\in G(Y\sharp {\mathbb {P}},Y(\cdot )) \end{array}}\int _{\Omega }\langle v(\omega ),D\psi (Y)(\omega )\rangle \,\mathrm{d}{\mathbb {P}}(\omega ),\end{aligned}$$

we obtain (recalling that $\Vert X\Vert _{L^2_{{\mathbb {P}}}}=1$)

$$\begin{aligned}H^{\mathrm {inv}}_G(X,D\psi (X))=-\dfrac{3}{2}\pi \int _{\Omega }\langle X,X\rangle \,\mathrm{d}{\mathbb {P}}(\omega )=-\dfrac{3}{2}\pi .\end{aligned}$$

Thus,

$$\begin{aligned}(L+2)d_{{\mathscr {K}}}(X\sharp {\mathbb {P}})+H^{\mathrm {inv}}_G(X,D\psi (X))=-\dfrac{3}{2}\pi <0,\end{aligned}$$

and therefore ${\hat{U}}(\cdot )$ is not a supersolution of the invariance equation.

On the other hand, set (see Definition 6.1)

$$\begin{aligned}H^{\mathrm {viab}}_G(Y,Q)=-d_{{\mathscr {K}}}(Y\sharp {\mathbb {P}})-\mathop {{{\,\mathrm{inf}\,}}}\limits _{\begin{array}{c} v\in L^2_{{\mathbb {P}}}(\Omega )\\ v(\cdot )\in G(Y\sharp {\mathbb {P}},Y(\cdot )) \end{array}}\int _{\Omega }\langle v(\omega ),Q(\omega )\rangle \,\mathrm{d}{\mathbb {P}}(\omega ).\end{aligned}$$

For every $v\in L^2_{{\mathbb {P}}}(\Omega )$ with $v(\cdot )\in F(Y\sharp {\mathbb {P}},Y(\cdot ))\subseteq G(Y\sharp {\mathbb {P}},Y(\cdot ))$, it holds

$$\begin{aligned}H^{\mathrm {viab}}_G(Y,Q)\ge -d_{{\mathscr {K}}}(Y\sharp {\mathbb {P}})-\int _{\Omega }\langle v(\omega ),Q(\omega )\rangle \,\mathrm{d}{\mathbb {P}}(\omega ),\end{aligned}$$

and by taking the supremum in the right-hand side over the set

$$\begin{aligned}\{v\in L^2_{{\mathbb {P}}}(\Omega )\,:\,v(\cdot )\in F(Y\sharp {\mathbb {P}},Y(\cdot ))\},\end{aligned}$$

we obtain

$$\begin{aligned}H^{\mathrm {viab}}_G(Y,Q)\ge H^{\mathrm {inv}}_F(Y,Q),\end{aligned}$$

and therefore for every $\psi \in C^1(L^2_{{\mathbb {P}}})$ such that ${\hat{U}}-\psi$ attains a local minimum at $X\in L^2_{{\mathbb {P}}}$ it holds

$$\begin{aligned}(L+2)d_{{\mathscr {K}}}(X\sharp {\mathbb {P}})+H^{\mathrm {viab}}_G(X,D\psi (X))\ge (L+2)d_{{\mathscr {K}}}(X\sharp {\mathbb {P}})+H^{\mathrm {inv}}_F(X,D\psi (X))\ge 0.\end{aligned}$$

Thus, ${\mathscr {K}}$ is viable for *G*, as already noticed.

## Appendix A: Essential technical results

Here, we report the proofs of the preliminary results presented in Sect. 3 as well as other technical results which have been significantly used in order to prove the main propositions and theorems of the present paper. In our opinion, these results could also be interesting by themselves.

### A.1: Proof of Proposition 3.4

Let ${\varvec{\mu }}=\{\mu _t\}_{t\in [a,b]}$ be an admissible trajectory defined in [*a*, *b*]. According to the superposition principle (Theorem 8.2.1 in [2] or Theorem 1 in [16]), there exists ${\varvec{\eta }}\in {\mathscr {P}}({\mathbb {R}}^d\times \Gamma _{[a,b]})$ such that $\mu _t=e_t\sharp {\varvec{\eta }}$ for all $t\in [a,b]$ and, for ${\varvec{\eta }}$-a.e. $(x,\gamma )$, ${\dot{\gamma }}(t)\in F(\mu _t,\gamma (t))$, $\gamma (a)=x$.

Set $|F(\mu _s,x)|=\max \{|y|:\,y\in F(\mu _s,x)\}$. For ${\varvec{\eta }}$-a.e. $(x,\gamma )\in {\mathbb {R}}^d\times \Gamma _{[a,b]}$, we have

$$\begin{aligned}&|\gamma (t)-\gamma (s)|\le \int _s^t |{\dot{\gamma }}(\tau )|\,\mathrm{d}\tau \le \int _s^t\Big [|F(\mu _s,\gamma (s))|+LW_2(\mu _\tau ,\mu _s) +L|\gamma (\tau )-\gamma (s)|\Big ]\,\mathrm{d}\tau \\&\le (t-s)(K+L \mathrm {m}^{1/2}_2(\mu _s)+L|\gamma (s)|)+ L\int _s^tW_2(\mu _\tau ,\mu _s)\,\mathrm{d}\tau +L\int _s^t|\gamma (\tau )-\gamma (s)|\,\mathrm{d}\tau . \end{aligned}$$

Grönwall’s inequality yields

$$\begin{aligned}|(e_t-e_s)(x,\gamma )|=|\gamma (t)-\gamma (s)|\le g(t,s,|\gamma (s)|),\end{aligned}$$

where

$$\begin{aligned}g(t,s,r):=e^{L(t-s)}\left[ (t-s)(K+L\mathrm {m}^{1/2}_2(\mu _s)+L r)+L\int _s^tW_2(\mu _\tau ,\mu _s)\,\mathrm{d}\tau \right] .\end{aligned}$$

By taking the $L^2_{{\varvec{\eta }}}$-norm, $\Vert e_t-e_s\Vert _{L^2_{{\varvec{\eta }}}}\le g(t,s,\mathrm {m}^{1/2}_2(\mu _s))$, and so

$$\begin{aligned}\left\| \dfrac{e_t-e_s}{t-s}\right\| _{L^2_{{\varvec{\eta }}}}\le \dfrac{g(t,s,\mathrm {m}^{1/2}_2(\mu _s))}{t-s}.\end{aligned}$$

By continuity, the right-hand side tends to $K+2L \mathrm {m}^{1/2}_2(\mu _s)$ as $t\rightarrow s^+$, and so $t\mapsto \dfrac{e_t-e_s}{t-s}$ is uniformly bounded in $L^2_{{\varvec{\eta }}}$ in a right neighborhood of *s*.

Let $p\in {\mathbb {R}}^d$, $t\in ]s,b]$. For ${\varvec{\eta }}$-a.e. $(x,\gamma )\in {\mathbb {R}}^d\times \Gamma _{[a,b]}$, reasoning as in the first part of the proof of Lemma 6.3, we have

$$\begin{aligned} \begin{aligned}&\langle p,\dfrac{e_t-e_s}{t-s}(x,\gamma )\rangle =\dfrac{1}{t-s}\int _s^t \langle p,{\dot{\gamma }}(\tau )\rangle \,\mathrm {d}\tau \le \dfrac{1}{t-s}\int _s^t \sup _{v\in F(\mu _\tau ,\gamma (\tau ))}\langle p,v\rangle \,\mathrm {d}\tau \\ {}&\le \sup _{v\in F(\mu _s,\gamma (s))}\langle p,v\rangle +|p|\cdot \dfrac{L}{t-s}\int _s^t\left[ W_2(\mu _\tau ,\mu _s)+|\gamma (\tau )-\gamma (s)|\right] \,\mathrm {d}\tau . \end{aligned} \end{aligned}$$

Therefore,

$$\begin{aligned}&\dfrac{e_t-e_s}{t-s}(x,\gamma )\in F(\mu _s,\gamma (s))\\&\quad +\left[ \dfrac{L}{t-s}\int _s^t\left[ W_2(\mu _\tau ,\mu _s)+|(e_\tau -e_s)(x,\gamma )|\right] \,\mathrm{d}\tau \right] \cdot \overline{B(0,1)}. \end{aligned}$$

By Filippov’s theorem (Theorem 8.2.10 in [4]), there exists a Borel map $w:{\mathbb {R}}^d\times \Gamma _{[a,b]}\rightarrow {\mathbb {R}}^d$, satisfying $w(x,\gamma )\in F(\mu _s,\gamma (s))$ for ${\varvec{\eta }}$-a.e. $(x,\gamma )\in {\mathbb {R}}^d\times \Gamma _{[a,b]}$ such that

$$\begin{aligned}\left| \dfrac{e_t-e_s}{t-s}(x,\gamma )-w(x,\gamma )\right| \le \dfrac{L}{t-s}\int _s^t\left[ W_2(\mu _\tau ,\mu _s)+|(e_\tau -e_s)(x,\gamma )|\right] \,\mathrm{d}\tau .\end{aligned}$$

Thus,

$$\begin{aligned}\left\| \dfrac{e_t-e_s}{t-s}-w\right\| _{L^2_{{\varvec{\eta }}}}\le \dfrac{L}{t-s}\int _s^t\left[ W_2(\mu _\tau ,\mu _s)+g(\tau ,s,\mathrm {m}^{1/2}_2(\mu _s))\right] \,\mathrm{d}\tau .\end{aligned}$$

### A.2: Proof of Proposition 3.5

For a proof of the nonemptiness of the set ${\mathcal {A}}_{[a,b]}(\mu )$, we refer the reader to Theorem 1 in [16], where the authors perform a fixed point argument.

Let $\{{\varvec{\mu }}^{(n)}\}_{n\in {\mathbb {N}}}\subseteq {\mathcal {A}}_{[a,b]}(\mu _0)$. By Proposition 3.4(2), for any $n\in {\mathbb {N}}$ there exists $\varvec{\eta }^{(n)}\in {\mathscr {P}}({\mathbb {R}}^d\times \Gamma _{[a,b]})$ such that $e_t\sharp \varvec{\eta }^{(n)}=\mu _t^{(n)}$ for all $t\in [a,b]$. Moreover, for $s\in [a,b]$ with $s<t$, we have

$$\begin{aligned} \begin{aligned} \Vert e_t-e_s\Vert _{L^2_{\varvec{\eta }^{(n)}}}\le&{} e^{L(t-s)}\left[ (t-s)(K+2L \mathrm {m}^{1/2}_2(\mu ^{(n)}_s))+\right. \\ {}&\quad \left. +L\displaystyle \int _s^tW_2(\mu ^{(n)}_\tau ,\mu ^{(n)}_s)\,\mathrm {d}\tau \right] =:h(t,s). \end{aligned} \end{aligned}$$

Notice that $W_2(\mu _t^{(n)},\mu _s^{(n)})\le \Vert e_t-e_s\Vert _{L^2_{\varvec{\eta }^{(n)}}}$. Indeed, it suffices to consider the admissible plan $\sigma :=(e_t,e_s)\sharp \varvec{\eta }^{(n)}\in \Pi (\mu ^{(n)}_t,\mu ^{(n)}_s)$. Thus,

$$\begin{aligned} W_2(\mu _t^{(n)},\mu _s^{(n)})\le h(t,s), \end{aligned}$$

and Grönwall’s inequality yields

A.1 $$\begin{aligned} W_2(\mu _t^{(n)},\mu _s^{(n)})\le e^{L(t-s)(1+e^{L(t-s)})}(t-s) (K+2L \mathrm {m}^{1/2}_2(\mu ^{(n)}_s)). \end{aligned}$$

By taking $s=a$, there exists ${\tilde{C}}>0$ such that

$$\begin{aligned} W_2(\mu _t^{(n)},\mu _0)\le {\tilde{C}}(1+\mathrm {m}_2^{1/2}(\mu _0))\quad \text {for any }t\in [a,b], \end{aligned}$$

and since $\mathrm {m}_2(\mu _0)<+\infty$, then we obtain uniform boundedness of $\{\varvec{\mu }^{(n)}\}_{n\in {\mathbb {N}}}$.

Moreover, by the triangle inequality and by recalling that $\mathrm {m}_2^{1/2}(\mu )=W_2(\mu ,\delta _0)$ by definition, we get for any $s\in [a,b]$

A.2 $$\begin{aligned} \mathrm {m}_2^{1/2}(\mu ^{(n)}_s)\le W_2(\mu ^{(n)}_s,\mu _0)+\mathrm {m}_2^{1/2}(\mu _0)\le (1+{\tilde{C}})(1+\mathrm {m}_2^{1/2}(\mu _0)). \end{aligned}$$

Thus, combining the previous estimate with (A.1), there exists ${\tilde{K}}>0$ such that

$$\begin{aligned}W_2(\mu _t^{(n)},\mu _s^{(n)})\le {\tilde{K}} (t-s),\end{aligned}$$

and hence $\varvec{\mu }^{(n)}$ are continuous for any $n\in {\mathbb {N}}$, with uniformly bounded Lipschitz constants. By the Ascoli–Arzelà Theorem, we conclude that, up to an unrelabeled subsequence, there exists ${\varvec{\mu }}=\{\mu _t\}_{t\in [a,b]}\in \mathrm {AC}([a,b];{\mathscr {P}}_2({\mathbb {R}}^d))$ such that $\sup _{t\in [a,b]}W_2(\mu ^{(n)}_t,\mu _t)\rightarrow 0$ as $n\rightarrow +\infty$.

We now prove the admissibility of ${\varvec{\mu }}$. Notice that, by (3.1) and (A.2), we have

A.3 $$\begin{aligned} \sup _{n\in {\mathbb {N}}}\int _{{\mathbb {R}}^d\times \Gamma _{[a,b]}}\left[ |x|+|\gamma (a)|+\Vert {\dot{\gamma }}\Vert _{L^{\infty }([a,b])}\right] \,\mathrm{d}{\varvec{\eta }}^{(n)}(x,\gamma )\le C' \mathrm {m}^{1/2}_2(\mu _0)+C'',\nonumber \\ \end{aligned}$$

for some constants $C',C''>0$. Moreover, the map

$$\begin{aligned}(x,\gamma )\mapsto {\left\{ \begin{array}{ll}|x|+|\gamma (a)|+\Vert {\dot{\gamma }}\Vert _{L^{\infty }([a,b])}, \text { if }\gamma \in \mathrm {Lip}([a,b]),\\ +\infty ,\text { otherwise},\end{array}\right. }\end{aligned}$$

has compact sublevels in ${\mathbb {R}}^d\times \Gamma _{[a,b]}$. Thus, by Remark 5.1.5 in [2], there exists ${\varvec{\eta }}\in {\mathscr {P}}({\mathbb {R}}^d\times \Gamma _{[a,b]})$ such that ${\varvec{\eta }}^{(n)}$ narrowly converges to ${\varvec{\eta }}$, up to (unrelabeled) subsequences. By Proposition 5.1.8 in [2], for any $(x,\gamma )\in \mathrm {supp}\,{\varvec{\eta }}$ there exists $\{(x_n,\gamma _n)\}_{n\in {\mathbb {N}}}\subseteq \mathrm {supp}\,{\varvec{\eta }}^{(n)}$ s.t. $x_n\rightarrow x$, $\gamma _n\rightrightarrows \gamma \in C([a,b];{\mathbb {R}}^d)$.

*Claim:* if $(x,\gamma )\in \mathrm {supp}\,{\varvec{\eta }}$, then $\gamma$ is a Lipschitz continuous solution of

A.4 $$\begin{aligned} {\left\{ \begin{array}{ll} {\dot{\gamma }}(t)\in F(\mu _t,\gamma (t)),&{}\text {for a.e. }t\in [a,b],\\ \gamma (a)=x. \end{array}\right. } \end{aligned}$$

Indeed, let ${\mathcal {N}}:=\bigcup _{n\in {\mathbb {N}}}{\mathcal {N}}^{(n)}\subseteq [a,b]$, where

$$\begin{aligned}{\mathcal {N}}^{(n)}:=\{s\in [a,b]\,:\,\not \exists \dot{\gamma }_n(s)\text { or }{\dot{\gamma }}_n(s)\not \in F(\mu _s^{(n)},\gamma _n(s))\}.\end{aligned}$$

Notice that for any $n\in {\mathbb {N}}$, ${\mathcal {N}}^{(n)}$ is a negligible set w.r.t. the Lebesgue’s measure, hence so is ${\mathcal {N}}$. Take $t\in [a,b]\setminus {\mathcal {N}}$. By Proposition 3.4, we have that ${\dot{\gamma }}_n(t)\in F(\mu _t^{(n)},\gamma _n(t))$, and by assumption $\varvec{(F_1)}$, for any $\varepsilon >0$ there exists ${\bar{n}}$ s.t. for any $n\ge {\bar{n}}$

$$\begin{aligned}{\dot{\gamma }}_n(t)\in F(\mu _t^{(n)},\gamma _n(t))\subseteq F(\mu _t,\gamma (t))+\varepsilon \overline{B(0,R)}.\end{aligned}$$

In particular, by continuity of $t\mapsto F(\mu _t,\gamma (t))$ in [*a*, *b*] we have that $\{{\dot{\gamma }}_n(t)\}_{n\in {\mathbb {N}}}$ is uniformly bounded for a.e. $t\in [a,b]$. Hence, $\gamma _n$ are continuous for any $n\in {\mathbb {N}}$, with uniformly bounded Lipschitz constants, and $\{\gamma _n\}_{n\in {\mathbb {N}}}$ is uniformly bounded. By the Ascoli–Arzelà Theorem, we get that $\gamma$ is a Lipschitz curve.

We now prove that $\gamma$ solves (A.4). Take any $v\in {\mathbb {R}}^d$, and denote by $\sigma _A(v):=\sup _{z\in A}\langle v,z\rangle$ the support function of $A\subseteq {\mathbb {R}}^d$ at *v*. For any $a\le s < t\le b$, we have

$$\begin{aligned} \langle v,\frac{\gamma _n(t)-\gamma _n(s)}{t-s}\rangle&=\frac{1}{t-s}\int _s^t \langle v,{\dot{\gamma }}_n(\tau )\rangle \,\mathrm{d}\tau \le \frac{1}{t-s}\int _s^t \sigma _{F(\mu ^{(n)}_\tau ,\gamma _n(\tau ))}(v)\,\mathrm{d}\tau \\&\le \frac{1}{t-s}\int _s^t\left( \sigma _{F(\mu _\tau ,\gamma (\tau ))}(v) +L |v|\,\left[ W_2(\mu ^{(n)}_\tau ,\mu _\tau )+|\gamma _n(\tau )-\gamma (\tau )|\right] \right) \,\mathrm{d}\tau , \end{aligned}$$

where we used the Lipschitz continuity of *F* coming from $\varvec{(F_2)}$. By uniform in time convergence, passing to the limit as $n\rightarrow +\infty$, we have

$$\begin{aligned} \langle v,\frac{\gamma (t)-\gamma (s)}{t-s}\rangle&\le \frac{1}{t-s}\int _s^t\sigma _{F(\mu _\tau ,\gamma (\tau ))}(v)\,\mathrm{d}\tau \\&\le \frac{1}{t-s}\int _s^t\left( \sigma _{F(\mu _s,\gamma (s))}(v) +L |v|\,\left[ W_2(\mu _\tau ,\mu _s)+|\gamma (\tau )-\gamma (s)|\right] \right) \,\mathrm{d}\tau . \end{aligned}$$

Thus, for a.e. *s*, passing to the limit as $t\rightarrow s$, we get $\langle v,{\dot{\gamma }}(s)\rangle \le \sigma _{F(\mu _s,\gamma (s))}(v)$ for any $v\in {\mathbb {R}}^d$, whence ${\dot{\gamma }}(s)\in F(\mu _s,\gamma (s))$ as claimed.

Observe that, by continuity of $e_t$ and uniqueness of the narrow limit, we have that $\mu ^{(n)}_t=e_t\sharp {\varvec{\eta }}^{(n)}$ narrowly converges to $\mu _t=e_t\sharp {\varvec{\eta }}$ for any $t\in [a,b]$, up to subsequences (see Lemma 5.2.1 in [2]). The rest of the proof is an adaptation of the proof of Theorem 1 in [13]. In order to conclude the admissibility of ${\varvec{\mu }}$, we notice that $t\mapsto \mu _t$ is a Lipschitz continuous map, indeed

$$\begin{aligned} W_2^2(\mu _t,\mu _s)&\le \int _{{\mathbb {R}}^d\times {\mathbb {R}}^d}|x-y|^2\,\mathrm{d}(e_t,e_s)\sharp {\varvec{\eta }}\\&= \int _{\Gamma _{[a,b]}}|\gamma (t)-\gamma (s)|^2\,\mathrm{d}{\varvec{\eta }}(x,\gamma ) \le {\tilde{C}} |t-s|^2, \end{aligned}$$

by Lipschitz continuity of $\gamma$ in the support of ${\varvec{\eta }}$. According to Theorem 3.5 in [3], the map $t\mapsto \mu _t$ is differentiable almost everywhere in [*a*, *b*], and for all $\varphi \in C^1_c({\mathbb {R}}^d)$

$$\begin{aligned} \frac{d}{dt}\int _{{\mathbb {R}}^d}\varphi (x)\,\mathrm{d}\mu _t(x)&=\frac{d}{dt}\int _{{\mathbb {R}}^d}\varphi (\gamma (t))\,\mathrm{d}{\varvec{\eta }}(x,\gamma ) = \\ \iint _{{\mathbb {R}}^d\times \Gamma _{[a,b]}}\nabla \varphi (\gamma (t))\cdot {\dot{\gamma }}(t)\,\mathrm{d}{\varvec{\eta }}(x,\gamma )&=\int _{{\mathbb {R}}^d}\nabla \varphi (y)\int _{e_t^{-1}(y)}{\dot{\gamma }}(t)\,\mathrm{d}\eta _{t,y}(x,\gamma )\,\mathrm{d}\mu _t(y), \end{aligned}$$

where $\{\eta _{t,y}\}_{y\in {\mathbb {R}}^d}\subseteq {\mathscr {P}}({\mathbb {R}}^d\times \Gamma _{[a,b]})$ is the disintegration of ${\varvec{\eta }}$ w.r.t. the evaluation operator $e_t$, i.e., ${\varvec{\eta }}=\mu _t\otimes \eta _{t,y}$. Finally, notice that the vector field

$$\begin{aligned}v_t(y):=\int _{e_t^{-1}(y)}{\dot{\gamma }}(t)\,\mathrm{d}\eta _{t,y}(x,\gamma )\end{aligned}$$

is well-defined for a.e. $t\in [a,b]$ and $\mu _t$-a.e. $y\in {\mathbb {R}}^d$, moreover, by convexity of $F(\mu _t,y)$, we can use Jensen’s inequality to get that $v_t(y)\in F(\mu _t,y)$ for a.e. *t* and $\mu _t$-a.e. *y*. Hence the conclusion.

### A.3: Technical results

#### Corollary A.1

Assume $\varvec{(F_1)-(F_2)}$. Let ${\varvec{\mu }}=\{\mu _t\}_{t\in [a,b]}$ be an admissible trajectory, with $0\le a< b<+\infty$. Then, there exists a family of random variables $\{X_t\}_{t\in [a,b]}\subseteq L^2_{{\mathbb {P}}}(\Omega )$ such that $X_t\sharp {\mathbb {P}}=\mu _t$ for all $t\in [a,b]$, and

$$\begin{aligned}&\dfrac{X_t-X_s}{t-s}(\omega )\in F(X_s\sharp {\mathbb {P}},X_s(\omega )) +\left[ \dfrac{L}{t-s}\int _s^t\left[ W_2(\mu _\tau ,\mu _s)+|X_\tau (\omega )-X_s(\omega )|\right] \,\mathrm{d}\tau \right] \cdot \overline{B(0,1)},\\&\Vert X_t-X_s\Vert _{L^2_{{\mathbb {P}}}}\le e^{L(t-s)}\left[ (t-s)(K+2L \mathrm {m}^{1/2}_2(\mu _s))+L\int _s^tW_2(\mu _\tau ,\mu _s)\,\mathrm{d}\tau \right] , \end{aligned}$$

for all $t,s\in [a,b]$, with $s<t$.

In particular, for every $p(\cdot )\in L^2_{{\mathbb {P}}}(\Omega )$ we have

A.5 $$\begin{aligned}&\liminf _{t\rightarrow s^+}\langle p,\dfrac{X_t-X_s}{t-s}\rangle _{L^2_{{\mathbb {P}}}}\ge \int _{\Omega }\mathop {{{\,\mathrm{inf}\,}}}\limits _{v\in F(X_s\sharp {\mathbb {P}},X_s(\omega ))} \langle p(\omega ),v\rangle \,\mathrm{d}{\mathbb {P}}(\omega ),\end{aligned}$$

A.6 $$\begin{aligned}&\limsup _{t\rightarrow s^+}\langle p,\dfrac{X_t-X_s}{t-s}\rangle _{L^2_{{\mathbb {P}}}}\le \int _{\Omega }\sup _{v\in F(X_s\sharp {\mathbb {P}},X_s(\omega ))} \langle p(\omega ),v\rangle \,\mathrm{d}{\mathbb {P}}(\omega ). \end{aligned}$$

#### Proof

Let ${\varvec{\mu }}=\{\mu _t\}_{t\in [a,b]}$ be an admissible trajectory defined in [*a*, *b*], and ${\varvec{\eta }}\in {\mathscr {P}}({\mathbb {R}}^d\times \Gamma _{[a,b]})$ be as in Proposition 3.4, with $\mu _t=e_t\sharp {\varvec{\eta }}$ for $t\in [a,b]$. In particular, see, e.g., Lemma 5.29 in [11], there exists a Borel map ${\mathscr {V}}:\Omega \rightarrow {\mathbb {R}}^d\times \Gamma _{[a,b]}$ such that ${\varvec{\eta }}={\mathscr {V}}\sharp {\mathbb {P}}$, and thus $\mu _t=X_t\sharp {\mathbb {P}}$ for all $t\in [a,b]$, where $X_t=e_t\circ {\mathscr {V}}$. Evaluating the estimates obtained in Proposition 3.4 for $(x,\gamma )={\mathscr {V}}(\omega )$, and recalling that $X_t=e_t\circ {\mathscr {V}}$, $X_s=e_s\circ {\mathscr {V}}$, we obtain

$$\begin{aligned} \begin{aligned}&\dfrac{X_t-X_s}{t-s}(\omega )\in F(X_s\sharp {\mathbb {P}},X_s(\omega ))+\\ {}&\qquad +\left[ \dfrac{L}{t-s}\int _s^t\left[ W_2(\mu _\tau ,\mu _s)+|X_\tau (\omega )-X_s(\omega )|\right] \,\mathrm {d}\tau \right] \cdot \overline{B(0,1)},\\ {}&\Vert X_t-X_s\Vert _{L^2_{{\mathbb {P}}}}\le e^{L(t-s)}\left[ (t-s)(K+2L \mathrm {m}^{1/2}_2(\mu _s))+L\int _s^tW_2(\mu _\tau ,\mu _s)\,\mathrm {d}\tau \right] . \end{aligned} \end{aligned}$$

Thus, for every $p(\cdot )\in L^2_{{\mathbb {P}}}(\Omega )$, we have

A.7 $$\begin{aligned} \begin{aligned}&\langle p,\dfrac{X_t-X_s}{t-s}\rangle _{L^2_{{\mathbb {P}}}}=\int _{\Omega }\langle p(\omega ),\dfrac{X_t-X_s}{t-s}(\omega )\rangle \,\mathrm{d}{\mathbb {P}}(\omega )\\&\quad \ge \int _{\Omega }\mathop {{{\,\mathrm{inf}\,}}}\limits _{v\in F(X_s\sharp {\mathbb {P}},X_s(\omega ))} \langle p(\omega ),v\rangle \,\mathrm{d}{\mathbb {P}}(\omega )+\\&\qquad -\dfrac{L}{t-s}\int _{\Omega }|p(\omega )|\int _s^t \left[ W_2(\mu _\tau ,\mu _s)+|X_\tau (\omega )-X_s(\omega )|\right] \,\mathrm{d}\tau \,\mathrm{d}{\mathbb {P}}(\omega ) \\&\quad \ge \int _{\Omega }\mathop {{{\,\mathrm{inf}\,}}}\limits _{v\in F(X_s\sharp {\mathbb {P}},X_s(\omega ))} \langle p(\omega ),v\rangle \,\mathrm{d}{\mathbb {P}}(\omega )+\\&\qquad -\Vert p\Vert _{L^2_{{\mathbb {P}}}}\cdot \dfrac{L}{t-s}\int _s^t\left[ W_2(\mu _\tau ,\mu _s)+\Vert X_\tau -X_s\Vert _{L^2_{{\mathbb {P}}}}\right] \,\mathrm{d}\tau . \end{aligned} \end{aligned}$$

By taking the liminf as $t\rightarrow s^+$, and using the estimate on $\Vert X_\tau -X_s\Vert _{L^2_{{\mathbb {P}}}}$, we obtain

$$\begin{aligned}\liminf _{t\rightarrow s^+}\langle p,\dfrac{X_t-X_s}{t-s}\rangle _{L^2_{{\mathbb {P}}}}\ge \int _{\Omega }\mathop {{{\,\mathrm{inf}\,}}}\limits _{v\in F(X_s\sharp {\mathbb {P}},X_s(\omega ))} \langle p(\omega ),v\rangle \,\mathrm{d}{\mathbb {P}}(\omega ).\end{aligned}$$

In the same way, we prove the inequality for the limsup. $\square$

We recall the following well-known result, used to prove Corollary A.3.

#### Lemma A.2

(Lemma 5.23 p. 379 in [11]) Let ${\mathbb {P}}$ be an atomless Borel probability measure on $\Omega$, $X,Y\in L^2_{{\mathbb {P}}}(\Omega ;{\mathbb {R}}^d)$ two random variables with the same law, i.e., $X\sharp {\mathbb {P}}=Y\sharp {\mathbb {P}}$. Then for any $\varepsilon >0$, there exist two Borel measurable maps $r,r^{-1}:\Omega \rightarrow \Omega$ such that

- *r* and $r^{-1}$ are measure-preserving, i.e., $r\sharp {\mathbb {P}}=r^{-1}\sharp {\mathbb {P}}={\mathbb {P}}$;
- $$\mathbb P(\{\omega \in \Omega :\,r\circ r^{-1}(\omega )=r^{-1}\circ r(\omega )=\omega \})=1$$;
- $$\mathbb P(\{\omega \in \Omega :\,|X(\omega )-Y\circ r(\omega )|\le \varepsilon \})=1$$.

In particular, we have $\Vert X-Y\circ r\Vert _{L^2_{{\mathbb {P}}}}\le \varepsilon$.

#### Corollary A.3

Assume $\varvec{(F_1)-(F_2)}$. Let ${\varvec{\mu }}=\{\mu _t\}_{t\in [a,b]}$ be an admissible trajectory, and $X\in L^2_{{\mathbb {P}}}(\Omega )$ such that $X\sharp {\mathbb {P}}=\mu _a$. Let $\varvec{\eta }\in {\mathscr {P}}({\mathbb {R}}^d\times \Gamma _{[a,b]})$ such that $\mu _t=e_t\sharp \varvec{\eta }$ for any $t\in [a,b]$. Then, for every $\varepsilon >0$ there exists a family of random variables $\{Y_t\}_{t\in [a,b]}\subseteq L^2_{{\mathbb {P}}}(\Omega )$ such that

1. $$Y_t\sharp {\mathbb {P}}=\mu _t$$ for all $$t\in [a,b]$$;
2. $\Vert Y_a-X\Vert _{L^2_{{\mathbb {P}}}}\le \varepsilon$, and so it holds $\Vert Y_t-X\Vert _{L^2_{{\mathbb {P}}}}\le \Vert Y_t-Y_a\Vert _{L^2_{\mathbb P}}+\varepsilon$;
3. for every $t\in [a,b]$ and for every $p(\cdot )\in L^2_{\mathbb P}(\Omega )$ we have
  A.8 $$\begin{aligned} \begin{aligned} \langle p,\dfrac{Y_t-Y_a}{t-a}\rangle _{L^2_{{\mathbb {P}}}}&\ge \int _{\Omega }\mathop {{{\,\mathrm{inf}\,}}}\limits _{v\in F(X\sharp {\mathbb {P}},X(\omega ))} \langle p(\omega ),v\rangle \,\mathrm{d}\mathbb P(\omega )-(\widehat{\varpi }(t)+L\varepsilon )\Vert p\Vert _{L^2_{{\mathbb {P}}}},\\ \langle p,\dfrac{Y_t-Y_a}{t-a}\rangle _{L^2_{{\mathbb {P}}}}&\le \int _{\Omega }\sup _{v\in F(X\sharp {\mathbb {P}},X(\omega ))} \langle p(\omega ),v\rangle \,\mathrm{d}\mathbb P(\omega )+(\widehat{\varpi }(t)+L\varepsilon )\Vert p\Vert _{L^2_{{\mathbb {P}}}}, \end{aligned} \end{aligned}$$
  where
  $$\begin{aligned}{\widehat{\varpi }}(t):=\dfrac{L}{t-a}\int _a^t\left[ W_2(\mu _\tau ,\mu _a)+\Vert e_\tau -e_a\Vert _{L^2_{{\varvec{\eta }}}}\right] \,\mathrm{d}\tau .\end{aligned}$$

#### Proof

Fix $\varepsilon >0$. Let $\varvec{\eta }\in {\mathscr {P}}({\mathbb {R}}^d\times \Gamma _{[a,b]})$ represent $\varvec{\mu }$, i.e., $\mu _t=e_t\sharp \varvec{\eta }$. Since ${\mathbb {P}}$ is an atomless Borel probability measure on a Polish space, as already noticed, there exists a Borel map ${\mathscr {V}}:\Omega \rightarrow \mathbb R^d\times \Gamma _{[a,b]}$ such that $\varvec{\eta }={\mathscr {V}}\sharp {\mathbb {P}}$. Set $X_t=e_t\circ {\mathscr {V}}$ for all $t\in [a,b]$. Notice that for every measure-preserving map $r:\Omega \rightarrow \Omega$, we have $\varvec{\eta }=({\mathscr {V}}\circ r)\sharp {\mathbb {P}}$, since $r\sharp {\mathbb {P}}={\mathbb {P}}$. Moreover, $(X_t\circ r)\sharp {\mathbb {P}}=X_t\sharp {\mathbb {P}}=\mu _t$ for all $t\in [a,b]$. By Proposition 3.4, for $\varvec{\eta }$-a.e. $(x,\gamma )\in {\mathbb {R}}^d\times \Gamma _{[a,b]}$ it holds

$$\begin{aligned}\dfrac{e_t-e_a}{t-a}(x,\gamma )\in F(\mu _a,e_a(x,\gamma ))+\varpi (t,x,\gamma )\cdot \overline{B(0,1)}.\end{aligned}$$

where

$$\begin{aligned} \varpi (t,x,\gamma ):=\dfrac{L}{t-a}\int _a^t\left[ W_2(\mu _\tau ,\mu _a)+|(e_\tau -e_a)(x,\gamma )|\right] \,\mathrm{d}\tau , \end{aligned}$$

and so $\varpi (t,x,\gamma )\rightarrow 0^+$ as $t\rightarrow a^+$.

Evaluating at $(x,\gamma )={\mathscr {V}}\circ r(\omega )$, and recalling that $X_t=e_t\circ {\mathscr {V}}$, we obtain

$$\begin{aligned}&\left( \dfrac{X_t-X_a}{t-a}\right) \circ r(\omega )\in F(\mu _a,X_a\circ r(\omega ))+\varpi (t,{\mathscr {V}}\circ r(\omega ))\overline{B(0,1)}\\&\quad \subseteq F(\mu _a,X(\omega ))+\left[ \varpi (t,\mathscr {V}\circ r(\omega ))\right. \\&\qquad \left. +L|X(\omega )-X_a\circ r(\omega )|\right] \cdot \overline{B(0,1)}. \end{aligned}$$

Since $X\sharp {\mathbb {P}}=X_a\sharp {\mathbb {P}}=\mu _a$, by Lemma A.2 for any $\varepsilon >0$ we can choose a measure-preserving map $r=r_\varepsilon$ such that $|X(\omega )-X_a\circ r(\omega )|\le \varepsilon$ for ${\mathbb {P}}$-a.e. $\omega \in \Omega$. So we have

$$\begin{aligned}\left( \dfrac{X_t-X_a}{t-a}\right) \circ r_\varepsilon (\omega )\in F(X\sharp {\mathbb {P}},X(\omega ))+(\varpi (t,\mathscr {V}\circ r_\varepsilon (\omega ))+L\varepsilon )\cdot \overline{B(0,1)}.\end{aligned}$$

Let $Y_t=X_t\circ r_\varepsilon$ for all $t\in [a,b]$. Then, as seen in the proof of Corollary A.1, we have

$$\begin{aligned} \langle p, \dfrac{Y_t-Y_a}{t-a}\rangle _{L^2_{\mathbb P}}&\ge \int _{\Omega } \mathop {{{\,\mathrm{inf}\,}}}\limits _{v\in F(X\sharp {\mathbb {P}},X(\omega ))}\langle v,p(\omega )\rangle \,\mathrm{d}{\mathbb {P}}(\omega ) -(\Vert \varpi (t,\mathscr {V}\circ r_\varepsilon (\cdot ))\Vert _{L^2_{{\mathbb {P}}}}+L\varepsilon )\Vert p\Vert _{L^2_{\mathbb P}},\\ \langle p, \dfrac{Y_t-Y_a}{t-a}\rangle _{L^2_{\mathbb P}}&\le \int _{\Omega } \sup _{v\in F(X\sharp {\mathbb {P}},X(\omega ))}\langle v,p(\omega )\rangle \,\mathrm{d}{\mathbb {P}}(\omega ) +(\Vert \varpi (t,\mathscr {V}\circ r_\varepsilon (\cdot ))\Vert _{L^2_{{\mathbb {P}}}}+L\varepsilon )\Vert p\Vert _{L^2_{\mathbb P}}, \end{aligned}$$

for every $p\in L^2_{{\mathbb {P}}}(\Omega )$. Notice that

1. $$Y_t\sharp {\mathbb {P}}=X_t\circ r_\varepsilon \sharp \mathbb P=X_t\sharp {\mathbb {P}}=\mu _t$$ since $$r_\varepsilon \sharp \mathbb P={\mathbb {P}}$$;
2. we have
  $$\begin{aligned}\Vert Y_t-X\Vert _{L^2_{{\mathbb {P}}}}\le \Vert Y_t-Y_a\Vert _{L^2_{{\mathbb {P}}}}+\Vert Y_a-X\Vert _{L^2_{{\mathbb {P}}}}\le \Vert Y_t-Y_a\Vert _{L^2_{{\mathbb {P}}}}+\varepsilon ;\end{aligned}$$
3. it holds
  $$\begin{aligned} \Vert \varpi (t,{\mathscr {V}}\circ r(\cdot ))\Vert _{L^2_{\mathbb P}}\le \widehat{\varpi }(t)=\dfrac{L}{t-a}\int _a^t\left[ W_2(\mu _\tau ,\mu _a)+\Vert e_\tau -e_a\Vert _{L^2_{{\varvec{\eta }}}}\right] \,\mathrm{d}\tau . \end{aligned}$$

From here follows the conclusion. $\square$

#### Lemma A.4

Assume $\varvec{(F_1)-(F_2)}$. Let $\mu \in {\mathscr {P}}_2({\mathbb {R}}^d)$, $a\ge 0$ be fixed, and consider a continuous selection $v(\cdot )$ of $F(\mu ,\cdot )$. Then, there exist $T>a$ and $\varvec{{\hat{\eta }}}\in {\mathscr {P}}({\mathbb {R}}^d\times \Gamma _{[a,T]})$ such that, if we set ${\hat{\theta }}_t=e_t\sharp \varvec{{\hat{\eta }}}$ for all $t\in [a,T]$ and $\varvec{{\hat{\theta }}}=\{{\hat{\theta }}_t\}_{t\in [a,T]}$,

- $\varvec{{\hat{\theta }}}$ is an admissible trajectory with ${\hat{\theta }}_a=\mu$ defined on [*a*, *T*];
- for $\varvec{{\hat{\eta }}}$-a.e. $(x,\gamma )\in {\mathbb {R}}^d\times \Gamma _{[a,T]}$ we have $\gamma \in C^1([a,T])$ with ${\dot{\gamma }}(a)=v(x)$, and
  $$\begin{aligned} {\left\{ \begin{array}{ll} {\dot{\gamma }}(t)\in F({\hat{\theta }}_t,\gamma (t)), &{}\text {for a.e. }t\in [a,T],\\ \gamma (a)=x; \end{array}\right. } \end{aligned}$$
- $$\dfrac{e_t-e_a}{t-a}\rightarrow v\circ e_a$$ in $$L^2_{\varvec{{\hat{\eta }}}}$$ as $$t\rightarrow a^+$$.

#### Proof

Without loss of generality, we set $a=0$. According to Theorem 9.7.2 in [4], there exists a continuous map $f:{\mathscr {P}}_2({\mathbb {R}}^d)\times {\mathbb {R}}^d\times \overline{B(0,1)}\rightarrow {\mathbb {R}}^d$ and a constant *c* independent on *F* such that for all $(\theta ,x)\in {\mathscr {P}}_2({\mathbb {R}}^d)\times {\mathbb {R}}^d$ it holds

- $$F(\theta ,x)=\{f(\theta ,x,u):\, u\in \overline{B(0,1)}\}$$.
- for every $u\in \overline{B(0,1)}$, the map $(\theta ,x)\mapsto f(\theta ,x,u)$ is Lipschitz continuous with Lipschitz constant less than $c\cdot \mathrm {Lip}(F)$,
- $$|f(\theta ,x,u)-f(\theta ,x,v)|\le c\cdot |F(\theta ,x)|\cdot |u-v|$$.

In particular, for all $(\theta ,x)\in {\mathscr {P}}_2({\mathbb {R}}^d)\times {\mathbb {R}}^d$ we have

$$\begin{aligned}|f(\theta ,x,u)-f(\theta ,x,v)|\le c\cdot \left[ K+\mathrm {Lip}(F)\cdot \left( \mathrm {m}^{1/2}_2(\theta )+|x|\right) \right] \cdot |u-v|.\end{aligned}$$

Let

$$\begin{aligned} L=\mathrm {Lip}(F),&K=|F(\delta _0,0)|,&L'>2\left[ 2cL(1+cLTe^{cLT})\mathrm {m}^{1/2}_2(\mu )+cLT K e^{cLT}+K\right] , \end{aligned}$$

and let $T>0$ that will be fixed later. Given $(x,u)\in {\mathbb {R}}^d\times \overline{B(0,1)}$ and a Lipschitz curve ${\varvec{\theta }}=\{\theta _t\}_{t\in [0,T]}\subseteq {\mathscr {P}}_2({\mathbb {R}}^d)$ with $\theta _0=\mu$ and $\mathrm {Lip}({\varvec{\theta }})< L'$, denote by $\gamma _{{\varvec{\theta }},x,u}(\cdot )$ the unique solution of

$$\begin{aligned}{\dot{\gamma }}(t)=f(\theta _t,\gamma (t),u),&\gamma (0)=x.\end{aligned}$$

For any $({\hat{\mu }},q,r)\in {\mathscr {P}}_2({\mathbb {R}}^d)\times {\mathbb {R}}\times {\mathbb {R}}$ define

$$\begin{aligned} C_1({\hat{\mu }},q,r):=&\dfrac{cLL'}{2}r^2+cL r\mathrm {m}^{1/2}_2({\hat{\mu }})+cL rq+rK,\\ C_2({\hat{\mu }},q,r):=&K+L\left( L'r+\mathrm {m}^{1/2}_2({\hat{\mu }})+q+e^{cL r}C_1({\hat{\mu }},q,r)\right) ,\\ C_3({\hat{\mu }},q,r):=&e^{cLr}\left( 1+crC_2({\hat{\mu }},q,r)\right) . \end{aligned}$$

For all $0\le s\le t\le T$, $y\in {\mathbb {R}}^d$, we have

$$\begin{aligned} \begin{aligned}&\int _s^t|f(\theta _\tau ,y,u)|\,\mathrm {d}\tau \le \\\le&\int _s^t| f(\theta _\tau ,y,u)-f(\theta _s,y,u)|\,\mathrm {d}\tau +\int _s^t|f(\theta _s,y,u)-f(\delta _0,0,u)|\,\mathrm {d}\tau +(t-s)|f(\delta _0,0,u)|\,\mathrm {d}\tau \\\le&cL\int _s^t W_2(\theta _\tau ,\theta _s)\,\mathrm {d}\tau +cL (t-s)\mathrm {m}^{1/2}_2(\theta _s)+cL(t-s)|y|+(t-s)K \le C_1(\theta _s,|y|,t-s), \end{aligned} \end{aligned}$$

where we used Lipschitz-in-time continuity of ${\varvec{\theta }}$. Since

$$\begin{aligned}&|\gamma _{{\varvec{\theta }},x,u}(t)-\gamma _{{\varvec{\theta }},x,u}(s)|\le \int _s^t |f(\theta _\tau ,\gamma _{{\varvec{\theta }},x,u}(\tau ),u) -f(\theta _\tau ,\gamma _{{\varvec{\theta }},x,u}(s),u)|\,\mathrm{d}\tau +\\&\qquad +\int _s^t|f(\theta _\tau ,\gamma _{{\varvec{\theta }},x,u}(s),u)|\,\mathrm{d}\tau \le cL\int _s^t |\gamma _{{\varvec{\theta }},x,u}(\tau )-\gamma _{{\varvec{\theta }},x,u}(s)|\,\mathrm{d}\tau +C_1(\theta _s,|\gamma _{{\varvec{\theta }},x,u}(s)|,t-s), \end{aligned}$$

by Grönwall’s inequality,

$$\begin{aligned} |\gamma _{{\varvec{\theta }},x,u}(t)-\gamma _{{\varvec{\theta }},x,u}(s)|\le e^{cL(t-s)}C_1(\theta _s,|\gamma _{{\varvec{\theta }},x,u}(s)|,t-s). \end{aligned}$$

Choosing $s=0$, for all $t\in [0,T]$, we have

A.9 $$\begin{aligned} |\gamma _{{\varvec{\theta }},x,u}(t)|\le |x|+ e^{cL T}C_1(\mu ,|x|,T). \end{aligned}$$

This implies also

$$\begin{aligned} |F(\theta _t,\gamma _{{\varvec{\theta }},x,u}(t))|&\le K+L\left( W_2(\theta _t,\delta _0)+|\gamma _{{\varvec{\theta }},x,u}(t)|\right) \\&\le K+L(W_2(\theta _t,\mu )+\mathrm {m}_2^{1/2}(\mu )+|\gamma _{{\varvec{\theta }},x,u}(t)|) \le C_2(\mu ,|x|,T). \end{aligned}$$

Notice that the map $(x,u)\mapsto (x,\gamma _{{\varvec{\theta }},x,u})$ is locally Lipschitz continuous. Indeed,

$$\begin{aligned} \begin{aligned}&|\gamma _{{\varvec{\theta }},x_1,u}(t)-\gamma _{{\varvec{\theta }},x_2,v}(t)|\le \\\le&|x_1-x_2|+\int _0^t |f(\theta _\tau ,\gamma _{{\varvec{\theta }},x_1,u}(\tau ),u) -f(\theta _\tau ,\gamma _{{\varvec{\theta }},x_2,v}(\tau ),v)|\,\mathrm {d}\tau \\\le&|x_1-x_2|+\int _0^t |f(\theta _\tau ,\gamma _{{\varvec{\theta }},x_1,u}(\tau ),u)-f(\theta _\tau ,\gamma _{{\varvec{\theta }},x_1,u}(\tau ),v)|\,\mathrm {d}\tau +\\ {}&\qquad +\int _0^t|f(\theta _\tau ,\gamma _{{\varvec{\theta }},x_1,u}(\tau ),v)-f(\theta _\tau ,\gamma _{{\varvec{\theta }},x_2,v}(\tau ),v)|\,\mathrm {d}\tau \\\le&|x_1-x_2|+c\int _0^t|F(\theta _\tau ,\gamma _{{\varvec{\theta }},x_1,u}(\tau ))|\,\mathrm {d}\tau \cdot |u-v|+\\ {}&\qquad +cL\int _0^t |\gamma _{{\varvec{\theta }},x_1,u}(\tau )-\gamma _{{\varvec{\theta }},x_2,v}(\tau )|\,\mathrm {d}\tau \\\le&|x_1-x_2|+ctC_2(\mu ,|x_1|,T)\cdot |u-v|+cL\int _0^t |\gamma _{{\varvec{\theta }},x_1,u}(\tau )-\gamma _{{\varvec{\theta }},x_2,v}(\tau )|\,\mathrm {d}\tau . \end{aligned} \end{aligned}$$

By Grönwall’s inequality, for all $t\in [0,T]$ we have

$$\begin{aligned} |\gamma _{{\varvec{\theta }},x_1,u}(t)-\gamma _{{\varvec{\theta }},x_2,v}(t)|\le&e^{cLT}\left( 1+cTC_2(\mu ,|x_1|,T)\right) \left( |x_1-x_2|+|u-v|\right) \\ =&C_3(\mu ,|x_1|,T)\left( |x_1-x_2|+|u-v|\right) . \end{aligned}$$

This provides the Lipschitz continuity on all bounded subsets of ${\mathbb {R}}^d\times \overline{B(0,1)}$ by the continuity of $C_3(\mu ,\cdot ,T)$.

By Filippov’s Theorem (see Theorem 8.2.10 in [4]), there exists a Borel map $u:{\mathbb {R}}^d\rightarrow \overline{B(0,1)}$ such that $v(x)=f(\mu ,x,u(x))$ for all $x\in {\mathbb {R}}^d$. The map $x\mapsto \gamma _{{\varvec{\theta }},x,u(x)}$ is a composition of Borel maps, so it is Borel, and we define ${\varvec{\eta }}^{{\varvec{\theta }}}=\mu \otimes \delta _{\gamma _{{\varvec{\theta }},x,u(x)}}$. We have by construction that

- $$e_0\sharp {\varvec{\eta }}^{{\varvec{\theta }}}=\mu $$;
- for ${\varvec{\eta }}^{{\varvec{\theta }}}$-a.e. $(x,\gamma )\in {\mathbb {R}}^d\times \Gamma _T$ we have $\gamma \in C^1([0,T])$ with $\gamma (0)=x$ and ${\dot{\gamma }}(0)=v(x)$.

We want to show now that $t\mapsto e_t\sharp {\varvec{\eta }}^{{\varvec{\theta }}}$ is Lipschitz continuous with constant less than $L'$. Indeed, given $0\le s\le t\le T$ we have

$$\begin{aligned}&W_2(e_t\sharp {\varvec{\eta }}^{{\varvec{\theta }}},e_s\sharp {\varvec{\eta }}^{{\varvec{\theta }}})\le \Vert e_t-e_s\Vert _{L^2_{{\varvec{\eta }}^{{\varvec{\theta }}}}} =\left( \int _{{\mathbb {R}}^d\times \Gamma _T} |\gamma (t)-\gamma (s)|^2\,\mathrm{d}{\varvec{\eta }}^{{\varvec{\theta }}}(x,\gamma )\right) ^{1/2}\\&\quad \le e^{cLT}\left( \int _{{\mathbb {R}}^d\times \Gamma _T}|C_1(\theta _s,|\gamma (s)|,t-s)|^2\,\mathrm{d}{\varvec{\eta }}^{{\varvec{\theta }}}(x,\gamma )\right) ^{1/2}\\&\quad \le \dfrac{cLL'}{2}(t-s)^2+cL (t-s)\mathrm {m}^{1/2}_2(\theta _s)+cL (t-s)\left( \int _{{\mathbb {R}}^d\times \Gamma _T}|\gamma (s)|^2\,\mathrm{d}{\varvec{\eta }}^{{\varvec{\theta }}}(x,\gamma )\right) ^{1/2} +(t-s)K. \end{aligned}$$

Notice that

$$\begin{aligned}\mathrm {m}^{1/2}_2(\theta _s)=W_2(\theta _s,\delta _0)\le W_2(\theta _s,\theta _0)+W_2(\theta _0,\delta _0)\le L'T+\mathrm {m}^{1/2}_2(\mu ).\end{aligned}$$

Moreover, by (A.9), we have

$$\begin{aligned}&\left( \int _{{\mathbb {R}}^d\times \Gamma _T}|\gamma (s)|^2\,\mathrm{d}{\varvec{\eta }}^{{\varvec{\theta }}}(x,\gamma )\right) ^{1/2}\le \mathrm {m}_2^{1/2}(\mu ) +e^{cLT}\left( \int _{{\mathbb {R}}^d\times \Gamma _T}|C_1(\mu ,|x|,T)|^2\,\mathrm{d}{\varvec{\eta }}^{{\varvec{\theta }}}(x,\gamma )\right) ^{1/2}\\&\quad \le \mathrm {m}_2^{1/2}(\mu )+e^{cLT}\left[ \frac{cLL'}{2}T^2+2cLT\mathrm {m}_2^{1/2}(\mu )+TK\right] \\&\quad \le \frac{cLL'}{2}T^2 e^{cLT}+(1+2cLT e^{cLT})\,\mathrm {m}_2^{1/2}(\mu )+TK e^{cLT} =:\frac{cLL'}{2}T^2 e^{cLT}+ B(\mu ,T). \end{aligned}$$

Thus,

A.10 $$\begin{aligned} \begin{aligned}&W_2(e_t\sharp {\varvec{\eta }}^{{\varvec{\theta }}},e_s\sharp {\varvec{\eta }}^{{\varvec{\theta }}})\\&\qquad \le |t-s|\left( \dfrac{cLL'}{2}T+cL (L'T+\mathrm {m}^{1/2}_2(\mu ))+cL (\frac{cLL'}{2}T^2 e^{cLT}+ B(\mu ,T))+K\right) \\&\qquad \le |t-s|\left[ L'(\frac{3}{2}cLT+\frac{c^2L^2}{2}T^2e^{cLT}) +2cL(1+cLT e^{cLT})\mathrm {m}_2^{1/2}(\mu )+cLTK e^{cLT}+K\right] \\&\qquad =: S(\mu ,L',T)\,|t-s|. \end{aligned}\nonumber \\ \end{aligned}$$

In particular, since we choose

$$\begin{aligned}\frac{L'}{2}>2cL(1+cLTe^{cLT})\mathrm {m}^{1/2}_2(\mu )+cLT K e^{cLT}+K,\end{aligned}$$

there is $T>0$ such that $S(\mu ,L',T)<L'$, and so $t\mapsto e_t\sharp {\varvec{\eta }}^{{\varvec{\theta }}}$ is Lipschitz continuous with constant less than $L'$.

Define by recurrence a sequence of curves $\{{\varvec{\theta }}^{(n)}=\{\theta ^{(n)}_t\}_{t\in [0,T]}\}_{n\in {\mathbb {N}}}$ and of measures $\{{\varvec{\eta }}^{(n)}\}_{n\in {\mathbb {N}}}$ by setting $\theta ^{(0)}_t=\mu$ for all $t\in [0,T]$. Supposing that we have defined ${\varvec{\theta }}^{(n)}$, then we define ${\varvec{\eta }}^{(n)}=\mu \otimes \delta _{\gamma _{{\varvec{\theta }}^{(n)},x,u(x)}}$ and ${\varvec{\theta }}^{(n+1)}$ by setting $\theta ^{(n+1)}_t=e_t\sharp {\varvec{\eta }}^{(n)}$. Notice that, by construction, for all $n\in {\mathbb {N}}$, $\theta _0^{(n)}=\mu$ and ${\varvec{\theta }}^{(n)}$ is a Lipschitz continuous curve with $\mathrm {Lip}({\varvec{\theta }}^{(n)})<L'$, by (A.10).

Since we have the same estimate as in (A.3), then there exists ${\varvec{\eta }}\in {\mathscr {P}}({\mathbb {R}}^d\times \Gamma _T)$ and a subsequence ${\varvec{\eta }}^{(n_k)}$ such that ${\varvec{\eta }}^{(n_k)}$ narrowly converges toward ${\varvec{\eta }}$. As already observed, we also have that ${\varvec{\theta }}^{(n_k)}$ is a family of uniformly bounded and continuous curves, with uniformly bounded Lipschitz constants. Thus, it has a subsequence which is uniformly convergent to a Lipschitz curve ${\varvec{\theta }}=\{\theta _t\}_{t\in [0,T]}$. We now follow the same reasoning as in the last part of the proof of Proposition 3.5 with ${\varvec{\mu }}$ and ${\varvec{\mu }}^{(n)}$ replaced, respectively, by ${\varvec{\theta }}$ and ${\varvec{\theta }}^{(n_k)}$. For ${\varvec{\eta }}$-a.e. $(x,\gamma )$, we get that ${\dot{\gamma }}(t)\in F(\theta _t,\gamma (t))$ for a.e. $t\in [0,T]$, $\gamma (0)=x$, $\gamma \in C^1([0,T])$ and ${\dot{\gamma }}(0)=v(x)$. Thus, ${\varvec{\theta }}$ is an admissible trajectory and $\dfrac{e_t-e_0}{t}(x,\gamma )\rightarrow v(x)$, as $t\rightarrow 0^+$, for ${\varvec{\eta }}$-a.e. $(x,\gamma )\in {\mathbb {R}}^d\times \Gamma _T$. We also notice that $v(x)=v\circ e_0(x,\gamma )$ for ${\varvec{\eta }}$-a.e. $(x,\gamma )\in {\mathbb {R}}^d\times \Gamma _T$. Finally, recalling the estimates on the admissible trajectories provided in Proposition 3.4, by the Dominated Convergence Theorem we have that the convergence is actually in $L^2_{{\varvec{\eta }}}$. $\square$
